# Metabolism of Paeoniae Radix Rubra and its 14 constituents in mice

**DOI:** 10.3389/fphar.2022.995641

**Published:** 2022-10-04

**Authors:** Jing Zhang, Yang Lv, Jing Zhang, Wen-Jin Shi, Xu-Yan Guo, Jing-Jing Xu, Peng-Pu Wang, Xue-Tai Chen, Lin-Han Xiang, Feng Xu, Xuan Wang, Shao-Qing Cai

**Affiliations:** State Key Laboratory of Natural and Biomimetic Drugs, School of Pharmaceutical Sciences, Peking University, Beijing, China

**Keywords:** Paeoniae Radix Rubra, paeoniflorin, catechin, ellagic acid, methylgallate, *in vivo* metabolism

## Abstract

**Objective:** Paeoniae Radix Rubra (PRR) is a commonly used traditional Chinese medicine with the effects of clearing away heat, cooling the blood, and relieving blood stasis. To 1) elucidate the metabolites and metabolic pathways of PRR and its 14 main constituents in mice and 2) reveal the possible origins of the known effective forms of PRR and their isomers, the metabolism of PRR in mice was systematically studied for the first time.

**Methods:** PRR and its 14 constituents were administered to mice by gavage once a day for seven consecutive days, respectively. All urine and feces were collected during the 7 days of dosing, and blood was collected at 1 h after the last dose. Metabolites were detected and identified using high performance liquid chromatography with diode array detector and combined with electrospray ionization ion trap time-of-flight multistage mass spectrometry (HPLC-DAD-ESI-IT-TOF-MS^n^).

**Results:** In total, 23, 16, 24, 17, 18, 30, 27, 17, 22, 17, 33, 3, 8, 24, and 31 metabolites of paeoniflorin, albiflorin, oxypaeoniflorin, benzoylpaeoniflorin, hydroxybenzoylpaeoniflorin, benzoyloxypaeoniflorin, galloylpaeoniflorin, lactiflorin, epicatechin gallate, catechin gallate, catechin, ellagic acid, 3,3′-di-*O*-methylellagic acid, methylgallate, and PRR were respectively identified in mice; after eliminating identical metabolites, a total of 195 metabolites remained, including 8, 11, 25, 17, 18, 30, 27, 17, 21, 17, 1, 2, 8, 20, and 20 newly identified metabolites, respectively. The metabolic reactions of PRR and its 14 main constituents in mice were primarily methylation, hydrogenation, hydrolysis, hydroxylation, glucuronidation, and sulfation.

Conclusion: We elucidated the metabolites and metabolic pathways of PRR and its 14 constituents (e.g., paeoniflorin, catechin, ellagic acid, and gallic acid) in mice and revealed the possible origins of the 10 known effective forms of PRR and their isomers. The findings are of great significance to studying the mechanism of action and quality control of PRR.

## 1 Introduction

The effective forms of traditional Chinese medicines can be the original constituents or the active metabolites produced *in vivo* (Xu et al., 2022). The metabolism of traditional Chinese medicines is the key link between their phytochemistry *in vitro* and their pharmacological activity *in vivo*. Therefore, studying the metabolism of traditional Chinese medicines is crucial to understanding the forms of the medicine that exist and are active *in vivo* along with the mechanisms of action of traditional Chinese medicines.

Paeoniae Radix Rubra (PRR), obtained from the dried roots of *Paeonia lactiflora* Pall. or *Paeonia veitchii* Lynch, is a commonly used traditional Chinese medicine with the effects of clearing away heat, cooling the blood, and relieving blood stasis (Zhao et al., 2021). PRR has many pharmacological effects, such as preventing liver fibrosis, curing jaundice, improving cholestasis in rats, relieving inflammation, and improving myocardial infarction, hypertrophy, and fibrosis (Tan et al., 2020).

The constituents of PRR have various structures, with the primary ones being monoterpene glycosides, tannins, flavonoids, and triterpenes (Yan et al., 2018). Monoterpene glycosides include paeoniflorin, albiflorin, oxypaeoniflorin, benzoylpaeoniflorin, hydroxybenzoylpaeoniflorin, benzoyloxypaeoniflorin, galloylpaeoniflorin, and lactiflorin. Tannins include ellagic acid and 3,3′-di*-O-*methylellagic acid. Catechins include catechin, catechin gallate, and epicatechin gallate. Gallic acids include methylgallate.

Among the 14 constituents mentioned above, 11 (all but hydroxybenzoylpaeoniflorin, benzoyloxypaeoniflorin, and lactiflorin) exhibit various biological activities. For example, paeoniflorin (the main active constituent of PRR) shows good anti-inflammatory, immunomodulatory, and anti-tumor effects (Zhao et al., 2021). Albiflorin has the function of soothing the liver and relieving depression (Zhao et al., 2018). Oxypaeoniflorin can prevent acute lung injury caused by lipopolysaccharides in mice (Fan et al., 2021). Benzoylpaeoniflorin exhibits anti-allergic activity, making it a potential candidate drug for the treatment of allergic diseases (Zhong et al., 2021). Galloylpaeoniflorin can relieve osteoporosis following oophorectomy (Liu et al., 2021b). Ellagic acid reduces the toxicity of diclofenac in rat hepatocytes by enhancing the activity of antioxidant enzymes such as catalase (Hatefi-Hesari et al., 2021). 3,3′-Di*-O-*methylellagic acid significantly reduces retinal vasodilation caused by high glucose levels in juvenile zebrafish (Lee et al., 2018). Methylgallate improves potassium oxazinate-induced kidney damage in mice with hyperuricemia nephropathy by inhibiting the NOD-like receptor thermal protein domain associated protein 3 (NLRP3) pathway, thereby producing a renal protective effect (Liu et al., 2021a). Catechin protects rat cardiomyocytes against hypoxic damage (Fang et al., 2018) and also exerts an anti-inflammatory effect (Syed Hussein et al., 2015). Both catechin gallate and epicatechin gallate have significant anti-inflammatory and anti-proliferative activities (Kurbitz et al., 2011). Catechin gallate inhibits catechol methylation in rat hepatocyte cytoplasm and hepatocyte cultures by inhibiting the activity of catechol oxymethyltransferase (Kadowaki et al., 2005).

In a previous study, we found that only four of the 21 forms of PRR that can effectively treat toxic heat and blood stasis were the original constituents; the other 17 were metabolites (Xu et al., 2022). The four original constituents were paeoniflorin (C1), oxypaeoniflorin (C2), desbenzoylpaeoniflorin isomer (C3), and 3,7/8-dimethylellagic acid (C4), and the 17 metabolites were 3′*-O-*methyl (epi) catechin 5*-O-*glucuronide (C5), 3-hydroxy phenylpropionic acid sulfate (C6), 3-hydroxy-4-methoxy-phenylpropionic acid sulfate (C7), 3/4-hydroxy benzoic acid sulfate (C8), C_10_H_18_O_2_ glucuronide (C9-C15), C_10_H_18_O_4_ glucuronide (C16), C_10_H_14_O_3_ glucuronide (C17), 3-methoxy-4-hydroxy-phenylpropionic acid sulfate (C18), C_8_H_8_O_3_ glucuronide (C19, C20), and benzoyl glucuronide (C21). Of them, C9–C17, C19, C20, and M23 are derived from paeoniflorin; C5–C7, C18, and C21 are possibly derived from catechins; and C8 is possibly derived from catechins or gallic acids (Liang et al., 2013).

In this study, we systematically explored the metabolism of PRR and its 14 constituents with four structure types (paeoniflorins, catechins, gallic acids, and ellagic acids) in mice. The objectives of the study were to 1) elucidate the metabolites and metabolic pathways of PRR and its 14 main constituents in mice, 2) determine the possible origins of the metabolites of PRR, and 3) clarify whether these compounds can be converted into the recognized effective forms *in vivo*. The findings are helpful for further elucidating the forms of PRR that exist *in vivo* and identifying the effective forms of PRR. The findings are also of great significance for studying the mechanisms of action and quality control of PRR.

## 2 Materials and methods

### 2.1 Medicinal materials and reagents

PRR was purchased from Baohua, a dealer of Chinese herbal medicines in Xunke County, Heilongjiang Province (Sample No. 6524). The medicine was identified as the dried roots of *Paeonia lactiflora* Pall. by Professor Shao-Qing Cai from Peking University School of Pharmaceutical Sciences. A voucher sample (No. 6524) was stored in the Herbarium of Peking University School of Pharmaceutical Sciences.

Paeoniflorin (Lot No. PS010957), albiflorin (Lot No. PS011455), oxypaeoniflorin (Lot No. PS010199), benzoylpaeoniflorin (Lot No. PS000157), hydroxyben zoylpaeoniflorin (Lot No. PS000693), benzoyloxypaeoniflorin (Lot No. PS012411), galloylpaeoniflorin (Lot No. PS010194), epicatechin gallate (Lot No. PS000163), catechin gallate (Lot No. PS010638) and 3,3′-di*-O-*methylellagic acid (Lot No. PS 22960025) were purchased from Chengdu Push Biotechnology Co., Ltd. (Chengdu, China). Catechin (Lot No. P21J11F 118,380) and methylgallate (Lot No. L19D5Y1) were obtained from Shanghai Yuanye Biotechnology Co., Ltd. (Shanghai, China). Lactiflorin (Lot No. MUST-21041202) and ellagic acid (Lot No. KN 960133) were purchased from Chengdu Must Biotechnology Co., Ltd. (Chengdu, China). The purity of all reference substances was greater than 95.0%, as indicated by high-performance liquid chromatography (HPLC; 254 nm). Formic acid (Lot No. 212271), acetonitrile (Lot No. F21LAL 202), and methanol (Lot No. 206409), all HPLC grade, were purchased from Thermo Fisher Scientific, Inc. (Waltham, MA, United States). HPLC-grade ethanol (Lot No. 32061) was purchased from Fuchen (Tianjin) Chemical Reagents Co., Ltd. (Tianjin, China). Sodium carboxymethyl cellulose (Lot No. A18105) was purchased from Sinopharm Chemical Reagents Co., Ltd. (Shanghai, China). Ultrapure water was prepared using a Milli-Q Integral 3 ultrapure water machine (Millipore, Billerica, MA, United States).

### 2.2 Preparation and qualitative analysis of the lyophilized powder of PRR decoction

The lyophilized powder of PRR decoction was prepared as described previously (Liang et al., 2013). Each Gram of the lyophilized powder was equivalent to 2.63 g of the crude drug. The constituents in the lyophilized powder of the PRR decoction were identified by high performance liquid chromatography with diode array detector and combined with electrospray ionization ion trap time-of-flight multistage mass spectrometry (HPLC-DAD-ESI-IT-TOF-MS^n^) with reference to the mass spectral data of the reference substances or the relevant literature (Supplementary Figure S1).

### 2.3 Animal and metabolic studies

Forty-eight ICR mice (male, 30 ± 2 g) were purchased from the Department of Laboratory Animal Sciences at the Peking University Health Science Center and randomized into one control group and 15 dosing groups (14 compound-dosed groups and one PRR decoction-dosed group) with three mice in each group. All mice were housed in mouse metabolism cages. The experiment lasted for 10 days. After adapting for the first 3 days, the mice were dosed by gavage once per day for the following 7 days. In the 14 compound-dosed groups, the dose was 40 mg/kg mouse weight, while the dose in the decoction-dosed group was 70 mg of lyophilized powder of PRR decoction per kg mouse weight (equivalent to 200 mg/kg of PRR crude drug). All compounds and the lyophilized powder of PRR decoction were suspended in 0.5% sodium carboxymethyl cellulose solution, and the dose volume was approximately 0.2 ml for each mouse. The control group was given the same volume of 0.5% sodium carboxymethyl cellulose solution. The mice were maintained in an environment at 22 ± 2°C (relative humidity 50 ± 5%) and allowed to eat and drink *ad libitum*. All animal experiments were approved by the Animal Ethics Committee of Peking University Health Science Center (approval number: LA2019117).

### 2.4 Collection and preparation of samples

#### 2.4.1 Collection of samples

All urine and feces were collected during the 7 days of dosing. One hour after the last dosing by gavage, the blood was collected into 1.5-ml heparin sodium-containing tubes by excising the eyeballs. All samples were stored in a refrigerator at −20°C prior to use.

#### 2.4.2 Preparation of urine, feces, and plasma samples

All urine, feces, and plasma samples from the same group were pooled, resulting in 16 urine, 16 feces, and 16 plasma samples. Each urine sample was centrifuged at 8,000 rpm at 4°C for 15 min. The supernatant was harvested, concentrated, and dried at 55°C followed by the addition of 10 ml of methanol and ultrasonic extraction for 30 min. The extract was filtered, concentrated, and dried at 55°C. Each feces sample was dried at 50°C for 48 h and smashed followed by the addition of 30 ml of methanol and ultrasonic extraction for 30 min per round (three rounds of extraction). The extract was filtered, and the filtrates obtained from the three rounds of extraction were pooled, concentrated, and dried at 55°C followed by the addition of 10 ml of methanol, ultrasonic extraction for 30 min, and centrifugation at 8,000 rpm and 4°C for 15 min. The supernatant was concentrated and dried at 55°C. The urine and feces samples (0.5 g each) were separately dissolved in 1 ml methanol. Each plasma sample was centrifuged at 5,000 rpm and 4°C for 15 min, and approximately 0.9 ml supernatant was collected for each group. After adding 4.5 ml methanol, the mixture was centrifuged at 5,000 rpm and 4°C for 15 min. The supernatant was blown with nitrogen and dried at 40°C followed by the addition of 0.5 ml methanol for reconstitution. All samples were filtered through a 0.22-μm membrane and stored at −20°C prior to further analysis.

### 2.5 Instruments and Conditions

HPLC-DAD-ESI-IT-TOF-MS^n^ analysis was performed using an HPLC instrument and an IT-TOF mass spectrometer connected to two LC-20AD pumps, an SIL-20AC autosampler, a CTO-20A column heater, an SPD-M20A photo-diode array (PDA) detector, and a CBM-20A system controller (Shimadzu, Kyoto, Japan). Data analysis was performed using LCMS Solution v.3.60, Formula Predictor v.1.2, and Accurate Mass Calculator (Shimadzu, Kyoto, Japan).

The chromatographic conditions were as follows: chromatographic column, Phenomenex Gemini C18 (250 mm × 4.6 mm, 5 µm); guard column, Phenomenex Security Guard (4 mm × 3.0 mm, 5.0 µm; Phenomenex, Torrance, CA, United States); column temperature, 40°C; injection volume, 10 μL; and flow rate, 1 ml/min. The mobile phase was 0.1% aqueous solution of formic acid (A) and acetonitrile (B). The mobile phase gradients were as follows: 0–12 min, 3% B; 12–33 min, 3%–8% B; 33–37.5 min, 8% B; 37.5–52.5 min, 8%–12% B; 52.5–82.5 min, 12%–25% B; 82.5–105 min, 25 %–60% B; 105–120 min, 60%–100% B; 120–130 min, 100% B. PDA detector: 200–700 nm. The mass spectrometry conditions were as follows: electrospray ionization (ESI), positive and negative ion mode; mass scan range, *m/z* 100–1,000 (MS), *m/z* 50–1,000 (MS^2^ and MS^3^); relative collision-induced dissociation (CID) energy, 50%; heater block temperature, 200°C; curved desolvation line (CDL) temperature, 200°C; detection voltage, 1.70 kV; interface voltage (+) 4.5 kV, (−) 3.5 kV; drying gas, nitrogen; and drying gas flow rate, 1.5 L/min.

### 2.6 Identification of the forms of PRR present *in vivo* (original constituents and metabolites)

The *in vivo* existence forms of PRR were identified as previously described (Liang et al., 2013). First, the base peak chromatograms (BPCs) of the samples from the dosing and control groups were compared to find the distinguishing peaks and tentatively determine the *in vivo* existence forms of PRR. Second, the extracted ion chromatograms (EICs) of the compounds in the dosing and control groups were compared to confirm the distinguishing peaks. The chromatographic peaks that appeared in the dosing groups but not in the control group were considered to represent the *in vivo* existence forms of PRR. Finally, the forms of PRR existing *in vivo* were analyzed structurally based on the obtained liquid chromatography-high resolution multi-stage mass spectrometry data combined with 1) the mass spectrometry data of reference substances, 2) the mass spectrometry fragmentation patterns, 3) the mass spectrometry fragmentation information reported in the literature, and 4) information obtained by searching the SciFinder database.

## 3 Results

In this study, 23, 16, 24, 17, 18, 30, 27, 17, 22, 17, 33, 3, 8, 24, and 31 metabolites of paeoniflorin (P), albiflorin (A), oxypaeoniflorin (O), benzoylpaeoniflorin (B), hydroxybenzoylpaeoniflorin (OB), benzoyloxypaeoniflorin (BO), galloylpaeoniflorin (G), lactiflorin (L), epicatechin gallate (ECG), catechin gallate (CG), catechin (C), ellagic acid (EA), 3,3′-di-O-methylellagic acid (DEA), methylgallate (MG), and PRR were respectively identified in mice. After identifying identical metabolites, a total of 195 metabolites remained (Table 1). The LC-MS^n^ data of the metabolites and the distributions of metabolites in urine, feces, and plasma of can be found in Supplementary Tables S1, S2.

**TABLE 1:** Retention times (t_R_) molecular formulae, and identities of 10 absorbed compounds and 195 metabolites of PRR and its 14 constituents in mice based on HPLC-ESI-IT-TOF-MS^n^.

No.	t_R_ (min)	Formula	Meas. (Da)	Err (ppm)	P	A	O	B	OB	BO	G	L	ECG	CG	C	EA	DEA	MG	PRR	Identification
P0^a^	56.58	C_23_H_28_O_11_	525.1590	−4.57	+	−	−	−	−	−	−	−	−	−	−	−	−	−	−	paeoniflorin
A0^a^	51.95	C_23_H_28_O_11_	525.1597	−3.24	−	+	−	−	−	−	−	−	−	−	−	−	−	−	−	albiflorin
O0^a^	42.99	C_23_H_28_O_12_	495.1501	−1.41	−	−	+	−	−	−	−	−	−	−	−	−	−	−	−	oxypaeoniflorin
OB0^a^	84.65	C_30_H_32_O_13_	599.1726	−7.34	−	−	−	−	+	−	−	−	−	−	−	−	−	−	−	hydroxybenzoylpaeoniflorin
BO0^a^	87.12	C_30_H_32_O_13_	599.1755	−2.5	−	−	−	−	−	+	−	−	−	−	−	−	−	−	−	benzoyloxypaeoniflorin
G0^a^	70.17	C_30_H_32_O_15_	631.1633	−5.55	−	−	−	−	−	−	+	−	−	−	−	−	−	−	−	galloylpaeoniflorin
L0^a^	81.32	C_23_H_26_O_10_	507.1508	0.00	−	−	−	−	−	−	−	+	−	−	−	−	−	−	−	lactiflorin
ECG0^a^	67.80	C_22_H_18_O_10_	441.0827	0.00	−	−	−	−	−	−	−	−	+	−	−	−	−	−	−	epicatechin gallate
CG0^a^	70.36	C_22_H_18_O_10_	441.0828	0.23	−	−	−	−	−	−	−	−	−	+	−	−	−	−	−	catechin gallate
DEA0^a^	90.97	C_16_H_10_O_8_	329.0315	3.65	−	−	−	−	−	−	−	−	−	−	−	−	+	−	−	3,3′-di*-O-*methylellagic acid
M1^a^	56.58	C_23_H_28_O_11_	525.1590	−4.57	−	−	+	+	+	−	+	−	−	−	−	−	−	−	−	paeoniflorin
M2^a^	42.53	C_23_H_28_O_12_	495.1502	−1.21	+	−	−	−	+	+	−	−	−	−	−	−	−	−	−	oxypaeoniflorin
M3	77.90	C_10_H_16_O_4_	199.0987	5.52	−	−	−	−	−	+	+	−	−	−	−	−	−	−	−	paeonimetabolin II isomer 1
M4	78.72	C_10_H_16_O_4_	199.0978	1.00	−	−	−	−	−	−	−	−	−	−	−	−	−	−	+	paeonimetabolin II isomer 2
M5	80.91	C_10_H_16_O_4_	199.0980	2.01	−	−	−	−	−	−	−	−	−	−	−	−	−	−	+	paeonimetabolin II isomer 3
M6	80.24	C_10_H_16_O_4_	199.0970	−3.01	−	−	−	−	−	+	+	−	−	−	−	−	−	−	−	paeonimetabolin II isomer 4
M7	23.69	C_10_H_16_O_7_S	279.0532	−4.30	−	+	−	−	−	−	−	−	−	−	−	−	−	−	−	paeonimetabolin II sulfate isomer 1
M8	31.89	C_10_H_16_O_7_S	279.0525	−6.81	−	−	−	−	+	−	−	−	−	−	−	−	−	−	−	paeonimetabolin II sulfate isomer 2
M9	43.84	C_10_H_16_O_7_S	279.0524	−7.17	−	+	−	−	−	−	−	−	−	−	−	−	−	−	−	paeonimetabolin II sulfate isomer 3
M10	49.22	C_10_H_16_O_7_S	279.0523	−7.53	−	+	−	−	−	−	+	−	−	−	−	−	−	−	−	paeonimetabolin II sulfate isomer 4
M11	8.77	C_16_H_24_O_10_	421.1361	2.14	−	+	−	−	−	−	−	−	−	−	−	−	−	−	−	desbenzoyl albiflorin isomer 1
M12	31.28	C_16_H_24_O_10_	375.1266	−8.26	−	+	−	−	−	−	−	−	−	−	−	−	−	−	−	desbenzoyl albiflorin isomer 2
M13	10.08	C_16_H_24_O_10_	421.1332	−4.75	−	+	−	−	−	−	−	−	−	−	−	−	−	−	−	desbenzoyl albiflorin isomer 3
M14	51.34	C_16_H_24_O_10_	421.1333	−4.51	+	+	+	+	+	−	−	−	−	−	−	−	−	−	−	desbenzoylpaeoniflorin isomer 1
M15	52.97	C_16_H_24_O_10_	421.1346	−1.42	+	+	+	+	+	+	+	+	−	−	−	−	−	−	−	desbenzoylpaeoniflorin isomer 2
M16	29.71	C_17_H_26_O_10_	435.1485	−5.29	+	−	−	−	−	−	−	−	−	−	−	−	−	−	−	methyl desbenzoylpaeoniflorin isomer 1
M17	26.15	C_17_H_26_O_10_	435.1477	−7.12	−	−	−	−	−	−	−	−	−	−	−	−	−	−	+	methyl desbenzoylpaeoniflorin isomer 2
M18	32.07	C_16_H_22_O_10_	373.1146	1.61	+	−	+	+	+	+	+	−	−	−	−	−	−	−	−	paeonimetabolin I glucuronide isomer 1
M19	33.01	C_16_H_22_O_10_	373.1145	1.34	−	−	+	+	+	+	+	−	−	−	−	−	−	−	−	paeonimetabolin I glucuronide isomer 2
M20	29.78	C_16_H_22_O_10_	373.1123	−4.56	−	−	−	−	−	−	−	+	−	−	−	−	−	−	−	paeonimetabolin I glucuronide isomer 3
M21	28.88	C_16_H_22_O_10_	373.1135	−1.34	−	−	−	−	−	−	−	+	−	−	−	−	−	−	−	paeonimetabolin I glucuronide isomer 4
M22	55.09	C_16_H_22_O_10_	373.1133	−1.88	−	−	+	−	−	−	−	−	−	−	−	−	−	−	−	paeonimetabolin I glucuronide isomer 5
M23	24.88	C_10_H_14_O_6_S	261.0437	−0.38	+	−	−	+	+	+	+	−	−	−	−	−	−	−	−	C_10_H_14_O_3_ sulfate isomer 1
M24	26.01	C_10_H_14_O_6_S	261.0438	0.00	+	−	−	+	+	+	+	−	−	−	−	−	−	−	−	C_10_H_14_O_3_ sulfate isomer 2
M25	28.75	C_10_H_14_O_6_S	261.0423	−5.75	−	−	+	−	−	−	−	−	−	−	−	−	−	−	−	C_10_H_14_O_3_ sulfate isomer 3
M26	29.29	C_10_H_14_O_6_S	261.0423	−5.75	+	−	+	−	+	−	−	−	−	−	−	−	−	−	−	C_10_H_14_O_3_ sulfate isomer 4
M27	34.56	C_10_H_14_O_6_S	261.0429	−3.45	+	−	−	+	+	−	−	−	−	−	−	−	−	−	−	C_10_H_14_O_3_ sulfate isomer 5
M28	37.82	C_10_H_14_O_6_S	261.0432	−2.3	−	−	+	−	−	−	−	−	−	−	−	−	−	−	−	C_10_H_14_O_3_ sulfate isomer 6
M29	24.33	C_16_H_26_O_10_	377.1475	5.83	−	−	+	−	+	+	−	−	−	−	−	−	−	−	−	C_10_H_18_O_4_ glucuronide isomer 1
M30	24.90	C_16_H_26_O_10_	377.1443	−2.65	−	−	−	−	−	+	+	−	−	−	−	−	−	−	−	C_10_H_18_O_4_ glucuronide isomer 2
M31	26.71	C_16_H_26_O_10_	377.1463	2.65	+	−	+	+	+	+	+	−	−	−	−	−	−	−	−	C_10_H_18_O_4_ glucuronide isomer 3
M32	30.81	C_16_H_26_O_10_	377.1453	0.00	−	−	−	−	−	+	+	−	−	−	−	−	−	−	−	C_10_H_18_O_4_ glucuronide isomer 4
M33	31.97	C_16_H_26_O_10_	377.1437	−4.24	+	−	+	+	−	+	+	−	−	−	−	−	−	−	−	C_10_H_18_O_4_ glucuronide isomer 5
M34	53.31	C_16_H_26_O_10_	377.1443	−2.65	−	−	−	−	−	−	−	+	−	−	−	−	−	−	+	C_10_H_18_O_4_ glucuronide isomer 6
M35	56.90	C_16_H_28_O_10_	379.1614	1.05	−	−	−	−	−	+	+	−	−	−	−	−	−	−	−	C_10_H_20_O_4_ glucuronide
M36	23.65	C_14_H_16_O_9_	327.0731	2.75	−	+	−	−	−	−	−	−	−	−	−	−	−	−	−	C_8_H_8_O_3_ glucuronide isomer 1
M37	31.11	C_14_H_16_O_9_	327.0712	−3.06	−	+	−	−	−	+	+	−	−	−	−	−	−	−	−	C_8_H_8_O_3_ glucuronide isomer 2
M38	32.32	C_14_H_16_O_9_	327.0722	0.00	−	+	−	−	−	−	−	−	−	−	−	−	−	−	−	C_8_H_8_O_3_ glucuronide isomer 3
M39	35.98	C_14_H_16_O_9_	327.0707	−4.59	−	+	−	−	−	−	−	−	−	−	−	−	−	−	−	C_8_H_8_O_3_ glucuronide isomer 4
M40	41.65	C_14_H_16_O_9_	327.0696	−7.95	−	−	−	−	−	+	+	−	−	−	−	−	−	−	−	C_8_H_8_O_3_ glucuronide isomer 5
M41	34.25	C_10_H_18_O_6_S	265.0730	−7.92	−	−	+	−	−	−	−	−	−	−	−	−	−	−	−	2,6-dihydroxycineol sulfate isomer 1
M42	47.90	C_10_H_18_O_6_S	265.0732	−7.17	+	−	+	−	+	−	−	−	−	−	−	−	−	−	−	2,6-dihydroxycineol sulfate isomer 2
M43	51.20	C_10_H_18_O_6_S	265.0736	−5.66	+	−	+	+	+	−	−	−	−	−	−	−	−	−	−	2,6-dihydroxycineol sulfate isomer 3
M44	54.17	C_10_H_18_O_6_S	265.0745	−2.26	+	−	+	+	−	−	−	−	−	−	−	−	−	−	−	2,6-dihydroxycineol sulfate isomer 4
M45	63.27	C_10_H_18_O_6_S	265.0729	−8.30	+	+	+	+	+	+	+	−	−	−	−	−	−	−	−	2,6-dihydroxycineol sulfate
M46	83.48	C_10_H_18_O_6_S	265.0739	−4.53	+	−	+	−	−	−	−	−	−	−	−	−	−	−	−	2,6-dihydroxycineol sulfate isomer 6
M47	85.97	C_10_H_18_O_6_S	265.0733	−6.79	+	+	+	+	−	+	+	−	−	−	−	−	−	−	−	2,6-dihydroxycineol sulfate isomer 7
M48	111.38	C_10_H_18_O_6_S	265.0740	−4.15	−	−	−	−	−	−	−	−	−	−	−	−	−	−	+	2,6-dihydroxycineol sulfate isomer 8
M49	128.78	C_10_H_18_O_6_S	265.0753	0.75	−	−	−	−	−	−	−	−	−	−	−	−	−	−	+	2,6-dihydroxycineol sulfate isomer 9
M50	69.08	C_16_H_26_O_9_	361.1522	4.98	−	−	−	−	−	−	−	−	−	−	−	−	−	−	+	paeonimetabolin II glucoside isomer 1
M51	71.20	C_16_H_26_O_9_	361.1486	−4.98	−	−	−	−	−	−	−	−	−	−	−	−	−	−	+	paeonimetabolin II glucoside isomer 2
M52	70.28	C_16_H_26_O_9_	361.1520	4.43	+	−	−	−	−	−	−	−	−	−	−	−	−	−	−	paeonimetabolin II glucoside isomer 3
M53	72.81	C_16_H_26_O_9_	361.1508	1.11	−	−	−	−	−	−	−	−	−	−	−	−	−	−	+	paeonimetabolin II glucoside isomer 4
M54	66.15	C_16_H_26_O_9_	361.1489	−4.15	−	−	−	−	−	+	+	−	−	−	−	−	−	−	−	paeonimetabolin II glucoside
M55	61.87	C_10_H_20_O_6_S	267.0936	0.00	+	+	+	−	+	+	+	−	−	−	−	−	−	−	−	hydrogenated 2,6-dihydroxycineol sulfate isomer 1
M56	57.88	C_10_H_20_O_6_S	267.0901	−2.62	−	−	−	−	−	−	−	−	−	−	−	−	−	−	+	hydrogenated 2,6-dihydroxycineol sulfate isomer 2
M57	38.30	C_16_H_24_O_9_	359.1318	−8.35	+	−	+	+	−	+	+	−	−	−	−	−	−	−	−	dehydrogenated 2,6-dihydroxycineol glucuronide isomer 1
M58	39.01	C_16_H_24_O_9_	359.1343	−1.39	+	−	+	+	−	+	+	−	−	−	−	−	−	−	−	dehydrogenated 2,6-dihydroxycineol glucuronide isomer 2
M59	39.61	C_16_H_24_O_9_	359.1342	−1.67	−	−	−	−	−	+	−	−	−	−	−	−	−	−	−	dehydrogenated 2,6-dihydroxycineol glucuronide isomer 3
M60	41.87	C_16_H_24_O_9_	359.1322	−7.24	+	−	+	−	−	+	+	−	−	−	−	−	−	−	−	dehydrogenated 2,6-dihydroxycineol glucuronide isomer 4
M61	48.65	C_16_H_24_O_9_	359.1333	−4.18	+	−	+	+	+	+	+	−	−	−	−	−	−	−	−	dehydrogenated 2,6-dihydroxycineol glucuronide
M62	49.63	C_16_H_24_O_9_	359.1353	1.39	−	−	−	−	−	+	−	−	−	−	−	−	−	−	−	dehydrogenated 2,6-dihydroxycineol glucuronide isomer 5
M63	32.99	C_7_H_6_O_8_S	248.9691	5.62	−	−	−	−	−	−	+	−	−	−	−	−	−	−	−	gallic acid sulfate
M64	42.42	C_7_H_6_O_8_S	248.9719	3.21	−	−	−	−	−	−	+	−	−	−	−	−	−	−	−	gallic acid sulfate
M65	37.17	C_9_H_9_NO_3_	178.0516	3.37	−	+	−	−	−	+	+	−	−	−	−	−	−	−	+	hippuric acid
M66	27.64	C_9_H_9_NO_4_	194.0469	5.15	−	−	−	−	−	+	−	−	−	−	−	−	−	−	−	hydroxyhippuric acid
M67	29.07	C_9_H_9_NO_4_	194.0459	0.00	−	−	−	−	−	+	−	−	−	−	−	−	−	−	−	hydroxyhippuric acid
M68	28.31	C_13_H_14_O_9_	313.0547	−5.75	−	−	−	−	−	+	−	−	−	−	−	−	−	−	−	salicylic acid glucuronide
M69	71.03	C_23_H_28_O_10_	509.1654	−2.16	−	−	−	−	−	−	−	+	−	−	−	−	−	−	−	hydrogenated lactiflorin
M70	63.56	C_23_H_28_O_10_	509.1652	−2.55	−	−	−	−	−	−	−	+	−	−	−	−	−	−	+	hydrogenated lactiflorin isomer
M71	71.64	C_23_H_28_O_11_	525.1598	−3.05	−	−	−	−	−	−	−	+	−	−	−	−	−	−	−	hydrogenated hydroxylated lactiflorin
M72	35.33	C_23_H_28_O_11_	525.1635	4.00	−	−	−	−	−	−	−	−	−	−	−	−	−	−	+	hydrogenated hydroxylated lactiflorin isomer 1
M73	33.67	C_23_H_28_O_11_	525.1624	1.90	−	−	−	−	−	−	−	−	−	−	−	−	−	−	+	hydrogenated hydroxylated lactiflorin isomer 2
M74	37.32	C_23_H_28_O_11_	525.1624	1.90	−	−	−	−	−	−	−	−	−	−	−	−	−	−	+	hydrogenated hydroxylated lactiflorin isomer 3
M75	85.18	C_17_H_18_O_8_S	381.0612	−1.05	−	−	−	−	−	−	−	+	−	−	−	−	−	−	−	hydrogenated deglycosylated lactiflorin sulfate isomer 1
M76	66.53	C_17_H_18_O_8_S	381.0625	2.36	−	−	−	−	−	−	−	+	−	−	−	−	−	−	−	hydrogenated deglycosylated lactiflorin sulfate isomer 2
M77	84.31	C_17_H_18_O_8_S	381.0622	1.57	−	−	−	−	−	−	−	+	−	−	−	−	−	−	−	hydrogenated deglycosylated lactiflorin sulfate isomer 3
M78	100.37	C_17_H_18_O_8_S	381.0629	3.41	−	−	−	−	−	−	−	+	−	−	−	−	−	−	−	hydrogenated deglycosylated lactiflorin sulfate isomer 4
M79^a^	40.07	C_15_H_14_O_6_	289.0706	−4.15	−	−	−	−	−	−	−	−	−	+	−	−	−	−	−	catechin
M80	71.09	C_15_H_14_O_9_S	369.0295	2.44	−	−	−	−	−	−	−	−	+	−	−	−	−	−	−	epicatechin sulfate
M81	66.12	C_15_H_14_O_9_S	369.0285	−0.27	−	−	−	−	−	−	−	−	−	+	−	−	−	−	−	catechin 5/7*-O-*sulfate isomer 1
M82	67.48	C_15_H_14_O_9_S	369.0268	−4.88	−	−	−	−	−	−	−	−	−	+	−	−	−	−	−	catechin sulfate isomer 2
M83	72.48	C_15_H_14_O_9_S	369.0280	−1.63	−	−	−	−	−	−	−	−	−	+	−	−	−	−	−	catechin sulfate isomer 3
M84	61.79	C_15_H_14_O_9_S	369.0283	−0.81	−	−	−	−	−	−	−	−	−	−	+	−	−	−	−	catechin 5/7*-O-*sulfate isomer 2
M85	68.11	C_15_H_14_O_9_S	369.0283	−0.81	−	−	−	−	−	−	−	−	−	−	+	−	−	−	−	catechin 3'/4′*-O-*sulfate isomer
M86	37.64	C_21_H_22_O_12_	465.1038	0.00	−	−	−	−	−	−	−	−	−	−	+	−	−	−	−	catechin glucuronide isomer 1
M87	36.14	C_21_H_22_O_12_	465.1008	−6.45	−	−	−	−	−	−	−	−	−	−	+	−	−	−	−	catechin glucuronide isomer 2
M88	32.94	C_21_H_22_O_12_	465.1053	3.23	−	−	−	−	−	−	−	−	−	−	+	−	−	−	−	catechin glucuronide isomer 3
M89	66.43	C_21_H_22_O_15_S	545.0599	−1.47	−	−	−	−	−	−	−	−	−	+	−	−	−	−	−	catechin glucuronide sulfate isomer 1
M90	61.43	C_21_H_22_O_15_S	545.0576	−5.69	−	−	−	−	−	−	−	−	−	−	+	−	−	−	−	catechin glucuronide sulfate isomer 2
M91	54.85	C_21_H_22_O_15_S	545.0621	2.57	−	−	−	−	−	−	−	−	−	−	+	−	−	−	−	catechin glucuronide sulfate isomer 3
M92	83.80	C_15_H_16_O_8_S	355.0485	−2.25	−	−	−	−	−	−	−	−	+	−	−	−	−	−	−	3-HPP-2-ol sulfate isomer 1
M93	85.03	C_15_H_16_O_8_S	355.0475	−5.07	−	−	−	−	−	−	−	−	+	−	−	−	−	−	−	3-HPP-2-ol sulfate isomer 2
M94	79.52	C_15_H_16_O_8_S	355.0473	−5.63	−	−	−	−	−	−	−	−	−	−	+	−	−	−	−	3-HPP-2-ol sulfate isomer 3
M95	40.82	C_12_H_14_O_12_S	381.0138	1.31	−	−	−	−	−	−	−	−	+	+	−	−	−	−	−	pyrogallol-*O*-glucuronide sulfate isomer 1
M96	41.84	C_12_H_14_O_12_S	381.0149	4.20	−	−	−	−	−	−	−	−	−	+	−	−	−	−	−	pyrogallol-*O*-glucuronide sulfate isomer 2
M97	50.13	C_11_H_14_O_4_	209.0812	−3.35	−	−	−	−	−	−	−	−	+	−	−	−	−	−	−	5-(3,4-dihydroxyphenyl)-valeric acid
M98	69.82	C_11_H_14_O_7_S	289.0386	−0.35	−	−	−	−	−	−	−	−	+	−	−	−	−	−	−	5-(3,4-dihydroxyphenyl)-valeric acid sulfate isomer 1
M99	125.97	C_11_H_14_O_7_S	289.0400	4.50	−	−	−	−	−	−	−	−	−	−	+	−	−	−	−	5-(3,4-dihydroxyphenyl)-valeric acid sulfate isomer 2
M100	66.80	C_11_H_14_O_7_S	289.0368	−6.57	−	−	−	−	−	−	−	−	−	−	+	−	−	−	−	5-(3,4-dihydroxyphenyl)-valeric acid sulfate isomer 3
M101	64.15	C_11_H_14_O_8_S	305.0348	3.61	−	−	−	−	−	−	−	−	+	−	−	−	−	−	−	trihydroxy benzenepentanoic acid sulfate isomer 1
M102	60.56	C_11_H_14_O_8_S	305.0316	−6.88	−	−	−	−	−	−	−	−	−	−	+	−	−	−	−	trihydroxy benzenepentanoic acid sulfate isomer 2
M103	79.45	C_11_H_12_O_7_S	287.0251	2.44	−	−	−	−	−	−	−	−	+	+	−	−	−	−	−	5-(3,4-dihydroxyphenyl)-γ-valerolactone sulfate isomer 1
M104	72.89	C_11_H_12_O_7_S	287.0243	4.18	−	−	−	−	−	−	−	−	+	−	−	−	−	−	−	5-(3,4-dihydroxyphenyl)-γ-valerolactone sulfate isomer 2
M105	77.34	C_11_H_12_O_7_S	287.0227	−1.39	−	−	−	−	−	−	−	−	−	+	+	−	−	−	−	5-(3,4-dihydroxyphenyl)-γ-valerolactone sulfate isomer 3
M106	76.08	C_11_H_12_O_7_S	287.0219	−4.18	−	−	−	−	−	−	−	−	−	−	+	−	−	−	−	5-(3,4-dihydroxyphenyl)-γ-valerolactone sulfate isomer 4
M107	46.82	C_17_H_20_O_10_	383.1002	4.70	−	−	−	−	−	−	−	−	+	−	−	−	−	−	−	5-(3,4-dihydroxyphenyl)-γ-valerolactone glucuronide isomer 1
M108	47.38	C_17_H_20_O_10_	383.1005	5.48	−	−	−	−	−	−	−	−	−	+	−	−	−	−	−	5-(3,4-dihydroxyphenyl)-γ-valerolactone glucuronide isomer 2
M109	76.97	C10H10O7S	273.0072	−0.73	−	−	−	−	−	−	−	−	+	+	−	−	−	−	−	ferulic acid sulfate
M110	87.39	C_11_H_12_O_6_S	271.0286	1.48	−	−	−	−	−	−	−	−	+	−	−	−	−	−	−	5-(3-hydroxyphenyl)-γ-valerolactone sulfate isomer 1
M111	82.81	C_11_H_12_O_6_S	271.0284	0.74	−	−	−	−	−	−	−	−	−	−	+	−	−	−	−	5-(3-hydroxyphenyl)-γ-valerolactone sulfate isomer 2
M112	78.53	C_23_H_20_O_10_	455.0985	0.22	−	−	−	−	−	−	−	−	−	+	−	−	−	−	−	methyl catechin gallate
M113	99.94	C_15_H_16_O_6_	291.0891	5.84	−	−	−	−	−	−	−	−	−	−	+	−	−	−	−	3,4-diHPP-2-ol
M114	95.61	C_15_H_16_O_6_	291.0866	−2.75	−	−	−	−	−	−	−	−	−	−	−	−	−	−	+	3,4-diHPP-2-ol isomer 1
M115	98.62	C_15_H_16_O_6_	291.0875	0.34	−	−	−	−	−	−	−	−	−	−	−	−	−	−	+	3,4-diHPP-2-ol isomer 2
M116	102.43	C_15_H_16_O_6_	291.0863	−3.78	−	−	−	−	−	−	−	−	−	−	−	−	−	−	+	3,4-diHPP-2-ol isomer 3
M117	108.16	C_15_H_16_O_6_	291.0885	3.78	−	−	−	−	−	−	−	−	−	−	−	−	−	−	+	3,4-diHPP-2-ol isomer 4
M118	110.64	C_15_H_16_O_6_	291.0880	2.06	−	−	−	−	−	−	−	−	−	−	−	−	−	−	+	3,4-diHPP-2-ol isomer 5
M119	125.68	C_15_H_16_O_6_	291.0882	2.75	−	−	−	−	−	−	−	−	−	−	−	−	−	−	+	3,4-diHPP-2-ol isomer 6
M120	95.25	C_21_H_24_O_15_S	547.1477	3.66	−	−	−	−	−	−	−	−	−	+	−	−	−	−	−	3,4-diHPP-2-ol glucuronide sulfate
M121	67.91	C_21_H_24_O_14_S	531.0796	−3.39	−	−	−	−	−	−	−	−	−	−	+	−	−	−	−	3-HPP-2-ol glucuronide sulfate
M122	85.09	C_16_H_16_O_9_S	383.0449	1.83	−	−	−	−	−	−	−	−	+	−	−	−	−	−	−	methyl catechin sulfate isomer 1
M123	82.21	C_16_H_16_O_9_S	383.0456	3.65	−	−	−	−	−	−	−	−	−	+	−	−	−	−	−	methyl catechin sulfate isomer 2
M124	81.83	C_16_H_16_O_9_S	383.0454	3.13	−	−	−	−	−	−	−	−	−	−	+	−	−	−	−	methyl catechin sulfate isomer 3
M125	75.73	C_16_H_16_O_9_S	383.0458	4.18	−	−	−	−	−	−	−	−	−	−	+	−	−	−	−	methyl catechin sulfate isomer 4
M126	77.29	C_16_H_16_O_9_S	383.0424	−4.70	−	−	−	−	−	−	−	−	−	−	+	−	−	−	−	methyl catechin sulfate isomer 5
M127	42.29	C_22_H_24_O_12_	479.1165	−6.26	−	−	−	−	−	−	−	−	−	−	+	−	−	−	−	methyl catechin glucuronide isomer 1
M128	51.77	C_22_H_24_O_12_	479.1171	−5.01	−	−	−	−	−	−	−	−	−	−	+	−	−	−	−	methyl catechin glucuronide isomer 2
M129	40.51	C_22_H_24_O_12_	479.1198	0.63	−	−	−	−	−	−	−	−	−	−	+	−	−	−	−	methyl catechin glucuronide isomer 3
M130	55.14	C_22_H_24_O_15_S	559.0763	0.00	−	−	−	−	−	−	−	−	−	−	+	−	−	−	−	methyl catechin glucuronide sulfate isomer 1
M131	53.57	C_22_H_24_O_15_S	559.0735	−5.01	−	−	−	−	−	−	−	−	−	−	+	−	−	−	−	methyl catechin glucuronide sulfate isomer 2
M132	65.48	C_22_H_24_O_15_S	559.0752	−1.97	−	−	−	−	−	−	−	−	−	−	+	−	−	−	−	methyl catechin glucuronide sulfate isomer 3
M133	81.08	C_9_H_8_O_6_S	242.9965	−1.65	−	−	−	−	−	−	−	−	+	−	−	−	−	−	−	*m*-coumaric acid sulfate
M134	56.30	C_7_H_6_O_6_S	216.9820	3.69	−	−	−	−	−	−	−	−	+	−	−	−	−	−	−	3/4-hydroxy benzonic acid sulfate isomer 1
M135	40.62	C_7_H_6_O_6_S	216.9809	−1.38	−	−	−	−	−	−	−	−	−	−	−	−	−	−	+	3/4-hydroxy benzonic acid sulfate isomer 2
M136	126.14	C_15_H_8_O_11_S	394.9707	−2.03	−	−	−	−	−	−	−	−	−	−	−	+	−	−	−	methyl ellagic acid sulfate isomer 1
M137	127.57	C_15_H_8_O_11_S	394.9710	−1.27	−	−	−	−	−	−	−	−	−	−	−	+	−	−	−	methyl ellagic acid sulfate isomer 2
M138	127.83	C_13_H_8_O_7_S	306.9927	2.93	−	−	−	−	−	−	−	−	−	−	−	−	+	−	−	urolithin A sulfate
M139	125.56	C_13_H_8_O_6_S	290.9960	−3.09	−	−	−	−	−	−	−	−	−	−	−	+	+	−	−	urolithin B sulfate isomer 1
M140	129.13	C_13_H_8_O_6_S	290.9959	−3.44	−	−	−	−	−	−	−	−	−	−	−	−	+	−	+	urolithin B sulfate isomer 2
M141	116.13	C_13_H_8_O_6_S	290.9947	−7.65	−	−	−	−	−	−	−	−	−	−	−	−	−	−	+	urolithin B sulfate isomer 3
M142^a^	126.77	C_16_H_10_O_8_	329.0300	−0.91	−	−	−	−	−	−	−	−	−	−	−	−	+	−	−	3,3′-di*-O-*methylellagic acid isomer
M143	128.12	C_16_H_10_O_11_S	408.9866	−1.22	−	−	−	−	−	−	−	−	−	−	−	−	+	−	−	3,3′-di*-O-*methylellagic acid sulfate isomer 1
M144	126.52	C_16_H_10_O_11_S	408.9867	−0.98	−	−	−	−	−	−	−	−	−	−	−	−	+	−	−	3,3′-di*-O-*methylellagic acid sulfate isomer 2
M145	73.88	C_22_H_18_O_14_	505.0627	−2.18	−	−	−	−	−	−	−	−	−	−	−	−	+	−	−	3,3′-di*-O-*methylellagic acid glucuronide isomer 1
M146	74.73	C_22_H_18_O_14_	505.0634	1.98	−	−	−	−	−	−	−	−	−	−	−	−	+	−	−	3,3′-di*-O-*methylellagic acid glucuronide isomer 2
M147	43.79	C_8_H_8_O_8_S	262.9867	0.00	−	−	−	−	−	−	−	−	−	−	−	−	−	+	−	methyl gallate sulfate
M148	45.96	C_8_H_8_O_8_S	262.9872	1.90	−	−	−	−	−	−	−	−	−	−	−	−	−	+	−	methyl gallate sulfate isomer 1
M149	59.29	C_8_H_8_O_8_S	262.9850	−6.46	−	−	−	−	−	−	−	−	−	−	−	−	−	+	−	methyl gallate sulfate isomer 2
M150	61.06	C_8_H_8_O_8_S	262.9867	0.00	−	−	−	−	−	−	−	−	−	−	−	−	−	+	−	methyl gallate sulfate isomer 3
M151	64.01	C_8_H_8_O_8_S	262.9852	−5.70	−	−	−	−	−	−	−	−	−	−	−	−	−	+	−	methyl gallate sulfate
M152	84.88	C_8_H_8_O_8_S	262.9862	−1.90	−	−	−	−	−	−	−	−	−	−	−	−	−	+	−	methyl gallate sulfate isomer 4
M153	88.43	C_8_H_8_O_8_S	262.9854	−4.94	−	−	−	−	−	−	−	−	−	−	−	−	−	+	−	methyl gallate sulfate isomer 5
M154	125.33	C_8_H_8_O_8_S	262.9864	−1.14	−	−	−	−	−	−	−	−	−	−	−	−	−	+	−	methyl gallate sulfate isomer 6
M155	127.18	C_8_H_8_O_8_S	262.9887	7.60	−	−	−	−	−	−	−	−	−	−	−	−	−	+	−	methyl gallate sulfate isomer 7
M156	36.25	C_14_H_16_O_11_	359.0622	0.56	−	−	−	−	−	−	−	−	−	−	−	−	−	+	−	methyl gallate glucuronide isomer 1
M157	37.21	C_14_H_16_O_11_	359.0620	0.00	−	−	−	−	−	−	−	−	−	−	−	−	−	+	−	methyl gallate glucuronide
M158	50.09	C_14_H_16_O_11_	359.0597	−6.41	−	−	−	−	−	−	−	−	−	−	−	−	−	+	−	methyl gallate glucuronide isomer 2
M159	26.75	C_14_H_16_O_11_	359.0618	−0.56	−	−	−	−	−	−	−	−	−	−	−	−	−	+	−	methyl gallate glucuronide isomer 3
M160	40.24	C_20_H_24_O_17_	535.0949	1.50	−	−	−	−	−	−	−	−	−	−	−	−	−	+	−	methyl gallate diglucuronide isomer 1
M161	38.77	C_20_H_24_O_17_	535.0943	0.37	−	−	−	−	−	−	−	−	−	−	−	−	−	+	−	methyl gallate diglucuronide isomer 2
M162	58.58	C_14_H_16_O_14_S	439.0199	2.51	−	−	−	−	−	−	−	−	−	−	−	−	−	+	−	methyl gallate sulfate glucuronide
M163	42.90	C_15_H_18_O_11_	373.0765	−2.95	−	−	−	−	−	−	−	−	−	−	−	−	−	+	−	methylated methyl gallate glucuronide isomer 1
M164	44.57	C_15_H_18_O_11_	373.0773	−0.80	−	−	−	−	−	−	−	−	−	−	−	−	−	+	−	methylated methyl gallate glucuronide isomer 2
M165	43.71	C_15_H_18_O_11_	373.0763	−3.48	−	−	−	−	−	−	−	−	−	−	−	−	−	+	−	methylated methyl gallate glucuronide isomer 3
M166	76.78	C_9_H_10_O_8_S	277.0014	−3.61	−	−	−	−	−	−	−	−	−	−	−	−	−	+	−	methylated methyl gallate sulfate isomer 1
M167	96.53	C_9_H_10_O_8_S	277.0015	−3.25	−	−	−	−	−	−	−	−	−	−	−	−	−	+	−	methylated methyl gallate sulfate isomer 2
M168	126.77	C_9_H_10_O_8_S	277.0018	−2.17	−	−	−	−	−	−	−	−	−	−	−	−	−	+	−	methylated methyl gallate sulfate isomer 3
M169	126.98	C_10_H_12_O_8_S	291.0165	−5.15	−	−	−	−	−	−	−	−	−	−	−	−	−	+	−	dimethylated methyl gallate sulfate
M170	81.24	C_7_H_8_O_4_S	187.0064	−3.74	−	−	−	−	−	−	−	−	−	−	−	−	−	−	+	benzyl alcohol sulfate
M171	50.12	C_8_H_8_O_6_S	230.9969	0.00	−	−	−	−	−	−	−	+	−	−	+	−	−	−	−	3/4-hydroxy phenylacetic acid sulfate isomer 1
M172	45.80	C_8_H_8_O_6_S	230.9952	−5.19	−	−	−	−	−	−	−	+	−	−	−	−	−	−	−	3/4-hydroxy phenylacetic acid sulfate isomer 2
M173	46.27	C_8_H_8_O_6_S	230.9953	−6.93	−	−	−	−	−	−	−	−	−	−	+	−	−	−	−	3/4-hydroxy phenylacetic acid sulfate isomer 3
M174	53.30	C_8_H_8_O_6_S	230.9952	−7.36	−	−	−	−	−	−	−	−	+	+	−	−	−	−	−	3/4-hydroxy phenylacetic acid sulfate isomer 4
M175	48.15	C_8_H_8_O_6_S	230.9962	−3.03	−	−	−	−	−	−	−	−	−	−	−	−	−	−	+	3/4-hydroxy phenylacetic acid sulfate isomer 5
M176	43.92	C_8_H_8_O_6_S	230.9962	−3.03	−	−	−	−	−	−	−	−	−	−	−	−	−	−	+	3/4-hydroxy phenylacetic acid sulfate isomer 6
M177	127.45	C_9_H_10_O_6_S	245.0129	1.63	−	−	−	−	−	−	−	+	−	−	−	−	−	−	−	3/4-hydroxy phenylpropionic acid sulfate isomer 1
M178	74.60	C_9_H_10_O_6_S	245.0126	0.41	−	−	−	−	−	−	−	−	+	+	−	−	−	−	−	3/4-hydroxy phenylpropionic acid sulfate isomer 2
M179	70.97	C_9_H_10_O_6_S	245.0117	−3.27	−	−	−	−	−	−	−	−	−	−	+	−	−	−	−	3/4-hydroxy phenylpropionic acid sulfate isomer 3
M180	69.45	C_9_H_10_O_6_S	245.0109	−6.53	−	−	−	−	−	−	−	−	−	−	+	−	−	−	−	3/4-hydroxy phenylpropionic acid sulfate isomer 4
M181	55.54	C_9_H_10_O_7_S	261.0059	−5.75	−	−	−	−	−	−	−	+	−	−	−	−	−	−	−	3,4-dihydroxy phenylpropionic acid sulfate isomer 1
M182	58.99	C_9_H_10_O_7_S	261.0083	3.45	−	−	−	−	−	−	−	−	+	−	−	−	−	−	−	3,4-dihydroxy phenylpropionic acid sulfate isomer 2
M183	67.81	C_9_H_10_O_7_S	261.0079	1.92	−	−	−	−	−	−	−	−	+	−	−	−	−	−	−	3,4-dihydroxy phenylpropionic acid sulfate isomer 3
M184	64.31	C_9_H_10_O_7_S	261.0088	−5.36	−	−	−	−	−	−	−	−	−	−	+	−	−	−	−	3,4-dihydroxy phenylpropionic acid sulfate isomer 4
M185	40.17	C_9_H_10_O_7_S	261.0082	3.07	−	−	−	−	−	−	−	−	−	−	−	−	−	−	+	3,4-dihydroxy phenylpropionic acid sulfate isomer 5
M186	53.09	C_9_H_10_O_7_S	261.0062	−4.60	−	−	−	−	−	−	−	−	−	−	−	−	−	−	+	3,4-dihydroxy phenylpropionic acid sulfate isomer 6
M187	44.94	C_8_H_8_O_7_S	246.9912	−2.43	−	−	−	−	−	−	−	+	−	−	−	−	−	−	−	3,4-dihydroxy phenylacetic acid sulfate isomer 1
M188	49.91	C_8_H_8_O_7_S	246.9918	0.00	−	−	−	−	−	−	−	−	+	+	−	−	−	−	−	3,4-dihydroxy phenylacetic acid sulfate isomer 2
M189	44.24	C_8_H_8_O_7_S	246.9924	2.43	−	−	−	−	−	−	−	−	−	−	+	−	−	+	−	3,4-dihydroxy phenylacetic acid sulfate isomer 3
M190	51.43	C_8_H_8_O_7_S	246.9925	2.83	−	−	−	−	−	−	−	−	+	−	−	−	−	−	−	3,4-dihydroxy phenylacetic acid sulfate isomer 4
M191	41.72	C_8_H_8_O_7_S	246.9910	−3.24	−	−	−	−	−	−	−	−	−	−	−	−	−	−	+	3,4-dihydroxy phenylacetic acid sulfate isomer 5
M192	125.83	C_12_H_16_O_8_S	319.0488	−1.57	−	−	−	−	−	−	−	+	−	−	+	−	−	−	−	dihydroxylated methoxylated benzenepentanoic acid sulfate isomer 1
M193	90.36	C_12_H_16_O_8_S	319.0507	4.39	−	−	−	−	−	−	−	−	+	−	−	−	−	−	−	dihydroxylated methoxylated benzenepentanoic acid sulfate isomer 2
M194	126.80	C_12_H_16_O_8_S	319.0490	−0.94	−	−	−	−	−	−	−	−	−	−	+	−	−	−	−	dihydroxylated methoxylated benzenepentanoic acid sulfate isomer 3
M195	124.59	C_12_H_16_O_8_S	319.0495	0.63	−	−	−	−	−	−	−	−	−	−	−	−	−	−	+	dihydroxylated methoxylated benzenepentanoic acid sulfate isomer 4
SUM					23	16	24	17	18	30	27	17	22	17	33	3	8	24	31	

Note: P, paeoniflorin; A, albiflorin; O, oxypaeoniflorin; B, benzoylpaeoniflorin; OB, hydroxybenzoylpaeoniflorin; BO, benzoyloxypaeoniflorin; G, galloylpaeoniflorin; L, lactiflorin; ECG, epicatechin gallate; CG, catechin gallate; C, catechin; EA, ellagic acid; DEA, 3,3′-di-*O*-methylellagic acid; MG, methyl gallate; PRR, Paeoniae Radix Rubra; t_R_, retention time; Meas., measured; Err., error.; +, detected; −, undetected; ^a^, identified by comparison with reference compounds.

In this study, the usual neutral losses in mass spectrometry were 30.01 Da (CH_2_O), 14.01 Da (CH_2_), 176.03 Da (C_6_H_8_O_6_), 79.95 Da (SO_3_), 81.97 Da (H_2_SO_3_), 43.99 Da (CO_2_), 15.02 Da (CH_3_•), 27.99 Da (CO), 42.01 Da (C_2_H_2_O), 46.01 Da (CH_2_O_2_), 104.01 Da (C_7_H_4_O), 122.04 Da (C_7_H_6_O_2_), and 162.05 Da (C_6_H_10_O_5_), indicating that a molecule which shows these neutral losses contains formaldehyde or methanyl, methyl, glucuronosyl, sulfonyl, sulfonyl, carboxy or lactone, methyl, carbonyl, acetyl, carboxy or lactone group, benzoyl, benzoyloxy, and hexosyl (more likely glucosyl) groups, respectively. In addition, the fragment ions at *m/z* 175.02 (C_6_H_7_O_6_) and *m/z* 96.96 (SO_4_H) indicate that the molecule contains glucuronosyl and sulfate groups.

### 3.1 Mass spectral features of the 14 reference substances

The mass spectral fragments and fragmentation pathways of the 14 reference substances are presented in Supplementary Table S3 and Supplementary Figure S2.

#### 3.1.1 Mass spectral features of eight paeoniflorins

The isomers paeoniflorin and albiflorin showed [M+HCOOH−H]^−^ at *m/z* 525.16 with a molecular formula of C_23_H_28_O_11_. The two isomers showed different relative abundances of the characteristic ions at *m/z* 449.14 and 479.15; for paeoniflorin, the relative abundance of the characteristic ion at *m/z* 449.14 was greater than that of the ion at *m/z* 479.15, whereas the opposite was observed for albiflorin. Oxypaeoniflorin showed [M−H]^−^ at *m/z* 495.15, and its molecular formula was predicted to be C_23_H_28_O_12_. The fragment ion at *m/z* 165.05 (C_9_H_10_O_3_) was formed by the neutral loss of C_14_H_18_O_9_ [150.04 Da (C_8_H_6_O_3_) and 180.06 Da (C_6_H_12_O_6_)].

Benzoylpaeoniflorin showed [M+HCOOH−H]^−^ at *m/z* 629.18, and its molecular formula was C_23_H_28_O_12_. The fragment ion at *m/z* 309.10 was formed by the neutral loss of 122.04 Da (C_7_H_6_O_2_, benzoic acid) from the ion at *m/z* 431.13.

The isomers benzoyloxypaeoniflorin and hydroxybenzoylpaeoniflorin showed [M−H]^−^ at *m/z* 599.17 with a molecular formula of C_30_H_32_O_13_. The two isomers showed differences in the relative abundances of the characteristic ions at *m/z* 477.14 and 447.13. For benzoyloxypaeoniflorin, the relative abundance of the characteristic ion at *m/z* 477.14 was higher than that at *m/z* 447.13, whereas the opposite was true for hydroxybenzoylpaeoniflorin.

Galloylpaeoniflorin showed [M−H]^−^ at *m/z* 631.16 with a molecular formula of C_30_H_32_O_15_. The fragment ion at *m/z* 509.12 was formed by a neutral loss of 122.04 Da (C_7_H_6_O_2_).

Lactiflorin showed [M−H]^−^ at *m/z* 461.13 with a molecular formula of C_23_H_26_O_10_. The fragment ions at *m/z* 371.11 and 339.11 were formed by neutral losses of 90.03 Da (C_3_H_6_O_3_) and 122.04 Da (C_7_H_6_O_2_), respectively.

#### 3.1.2 Mass spectral features of three catechins

Epicatechin gallate, catechin gallate, and catechin respectively showed [M−H]^−^ at *m/z* 441.08, 441.08, and 289.06 with molecular formulae of C_22_H_18_O_10_, C_22_H_18_O_10_, and C_15_H_14_O_6_. The cleavage pathways for these three catechins are the same as those described previously (Liu et al., 2009).

#### 3.1.3 Mass spectral features of two ellagic acids

Ellagic acid showed [M−H]^−^ at *m/z* 300.99 with a molecular formula of C_14_H_6_O_8_. The fragment ion at *m/z* 284.00 was formed by a neutral loss of 17.00 Da (OH•).

3,3′-Di*-O-*methylellagic acid showed [M−H]^−^ at *m/z* 329.03 with a molecular formula of C_16_H_10_O_8_. The fragment ions at *m/z* 314.01 and 298.98 were formed by two sequential losses of 15.02 Da (CH_3_•).

#### 3.1.4 Mass spectral features of one gallate

Methyl gallate showed [M−H]^−^ at *m/z* 183.03 with a molecular formula of C_8_H_8_O_5_. The fragment ions at *m/z* 168.01 and 124.02 were formed by neutral losses of 15.02 Da (CH_3_•) and 43.99 Da (CO_2_), respectively.

### 3.2 Identification of the metabolites of eight paeoniflorins (P, A, O, B, OB, BO, G, L).

**FIGURE 1:**
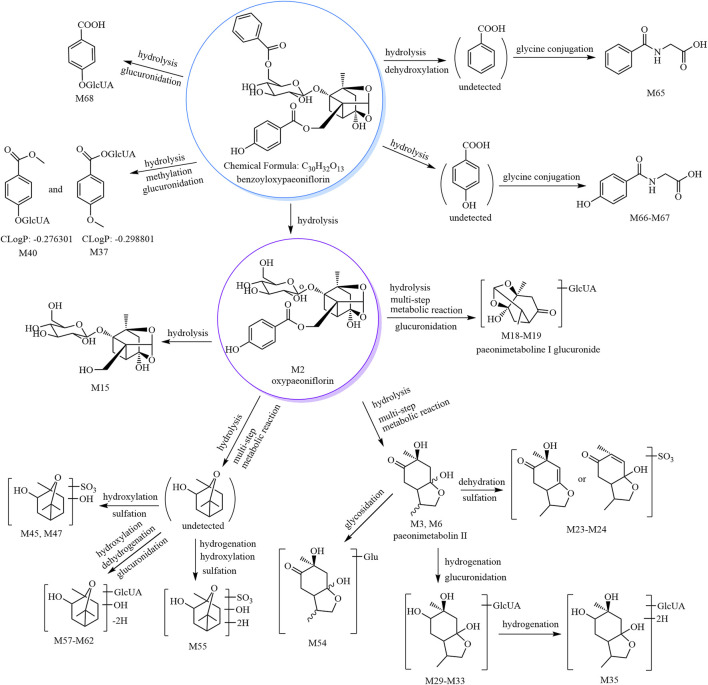
The 30 metabolites (all new) and proposed metabolic pathways of benzoyloxypaeoniflorin in mice.

The metabolic pathways of benzoyloxypaeoniflorin are shown in Figure 1, and the metabolic pathways of other paeoniflorins are shown in Supplementary Figure S3.

#### 3.2.1 Paeonimetabolin II (C_10_H_16_O_4_) and its phase II metabolites (M3–M10 and M50–M54)

According to our previous study, paeonimetabolin II showed [M−H]^−^ at *m/z* 199.09 with a molecular formula of C_10_H_16_O_4_. M3–M6 showed [M−H]^−^ at *m/z* 199.09 with a predicted molecular formula of C_10_H_16_O_4_; thus, M3-M6 were tentatively identified as paeonimetabolin II and its isomers (Liang et al., 2013). M7–M10 showed [M−H]^−^ at *m/z* 279.05 with a predicted molecular formula of C_10_H_16_O_7_S. The fragment ion at *m/z* 96.96 (HSO_4_) was observed in the MS^2^ spectra of M7, M9, and M10. Therefore, based on the mass spectral features and the literature (Shu et al., 1987; Liang et al., 2013), M7–M10 were tentatively identified as paeonimetabolin II sulfate isomers. M50–M54 showed [M−H]^−^ at *m/z* 361.15 with a predicted molecular formula of C_16_H_26_O_9_. The characteristic fragment at *m/z* 199.10 (C_10_H_16_O_4_, paeonimetabolin II) was formed by a neutral loss of 162.05 Da in the MS^2^ spectra of M54; therefore, M54 was tentatively identified as paeonimetabolin II glucoside. The other compounds, M50–M53, are M54 isomers and were tentatively identified as paeonimetabolin II glucoside isomers.

#### 3.2.2 Phase II metabolites of 2,6-dihydroxycineol (_C10H18O3_, epomediol or isomer) (M41–M49)

According to our previous identification of 2-hydroxy-1,8-cineol (C_10_H_18_O_2_) and our search of the SciFinder database, C_10_H_18_O_3_ was tentatively identified as 2,6-dihydroxycineol or epomediol. According to the literature (Jose Cuevas et al., 2002; Xu et al., 2022), epomediol reduces cholestasis. M41–M49 showed [M−H]^−^ at *m/z* 265.07, and the molecular formula was predicted as C_10_H_18_O_6_S. The characteristic fragment ion at *m/z* 183.09 (C_10_H_16_O_3_) was formed by a neutral loss of 81.97 Da (H_2_SO_3_) in the MS^2^ spectra of M45. Therefore, M45 was tentatively identified as 2,6-dihydroxycineol sulfate; M41–M44 and M46–M49 are isomers of M45 and were tentatively identified as 2,6-dihydroxycineol sulfate isomers.

#### 3.2.3 Derivatives of phase II metabolites of 2,6-dihydroxycineol (M55–M62)

M55 and M56 showed [M−H]^−^ at *m/z* 267.09, and their molecular formula was predicted as C_10_H_20_O_6_S. The characteristic fragment ion at *m/z* 169.09 (C_10_H_17_O_2_) was formed by a neutral loss of 97.99 Da (H_2_SO_4_) in the MS^2^ spectra of M56. Compared to M41–M49, M55 and M66 have two additional H atoms in their molecular formulae; thus, they were tentatively identified as hydrogenated 2,6-dihydroxycineol sulfate isomers.

M57–M62 showed [M−H]^−^ at *m/z* 359.13, and their molecular formula was predicted as C_16_H_24_O_9_. The characteristic ion at *m/z* 183.10 (C_10_H_16_O_3_) was formed by a neutral loss of 176.03 Da (C_6_H_8_O_6_) in the MS^2^ spectra of M61; thus, M61 was tentatively identified as dehydrogenated 2,6-dihydroxycineol glucuronide. M57–M60 and M62 are M61 isomers and were thus identified as dehydrogenated 2,6-dihydroxycineol glucuronide isomers.

#### 3.2.4 Lactiflorin-related metabolites (M69–M78)

M69 and M70 showed [M+HCOOH−H]^−^ at *m/z* 509.16, and their molecular formula was predicted as C_23_H_28_O_10_. The characteristic fragment ions at *m/z* 463.16 and 359.15 (C_16_H_24_O_9_) were formed by sequential losses of 46.01 Da (CH_2_O_2_) and 104.01 Da (C_7_H_4_O) in the MS^2^ spectra of M69. Based on the mass spectral features and origin of M69 (derived from lactiflorin), M69 was predicted as hydrogenated lactiflorin. M70 is an isomer of M69 and was predicted to be a hydrogenated lactiflorin isomer.

M71–M74 showed [M+HCOOH−H]^−^ at *m/z* 525.15, and their molecular formula was predicted as C_23_H_28_O_11_. The fragment ions at *m/z* 479.13 (C_23_H_27_O_11_) and 461.13 (C_23_H_25_O_10_) were formed by sequential losses of 46.01 Da (CH_2_O_2_) and 18.01 Da (H_2_O) in the MS^2^ spectra of M71. The ions at *m/z* 357.10 (C_16_H_21_O_9_) and *m/z* 339.09 (C_16_H_19_O_8_) were formed by sequential losses of 122.04 Da (C_7_H_6_O_2_, benzoic acid) and 18.01 Da (H_2_O) from the fragment at *m/z* 479.13 (C_23_H_27_O_11_). Based on the mass spectral features of M71 and its origin (derived from lactiflorin), M71 was predicted as a metabolite formed by the hydrogenation and hydroxylation of lactiflorin (i.e., M71 is a hydrogenated hydroxylated lactiflorin). M72–M74 are isomers of M71 and were thus predicted to be hydrogenated hydroxylated lactiflorin isomers.

M75–M78 showed [M−H]^−^ at *m/z* 381.06 with a predicted molecular formula of C_17_H_18_O_8_S. The fragment ions at *m/z* 259.01 (C_10_H_11_O_6_S) and *m/z* 195.07 (C_10_H_11_O_4_) were formed by neutral losses of 122.04 Da (C_7_H_6_O_2_) and 186.00 Da [106.04 Da (C_7_H_6_O) and 79.96 Da (SO_3_)] in the MS^2^ spectra. The fragment ions at *m/z* 177.03 (C_10_H_9_O_3_), *m/z* 165.04 (C_9_H_9_O_3_), and *m/z* 147.05 (C_9_H_7_O_2_) were formed by neutral losses of 18.01 Da (H_2_O), 30.01 Da (CH_2_O), and 48.02 (H_2_O and CH_2_O), respectively, from the ion at *m/z* 195.07 (C_10_H_11_O_4_). M75–M78 were all derived from the metabolism of lactiflorin and were thus predicted to be metabolites formed by the hydrogenation, deglycosylation, and sulfation of lactiflorin (i.e., M75–M78 are hydrogenated deglycosylated lactiflorin sulfate isomers).

#### 3.2.5 Other metabolites of paeoniflorins (M1–M2, M11–M40, M63–M68)

M1 and M2 showed [M+HCOOH−H]^−^ and [M−H]^−^ at *m/z* 525.16 and 495.15, respectively, and their molecular formulae were predicted as C_23_H_28_O_11_ and C_23_H_28_O_12_, respectively. Their retention time and mass spectral features were consistent with those of the reference substances paeoniflorin and oxypaeoniflorin (see Table 1 and Supplementary Table S1). Thus, M1 and M2 were respectively identified as paeoniflorin and oxypaeoniflorin.

As reported previously (Liang et al., 2013), M11–M13 are isomers of desbenzoylpaeoniflorin (C_16_H_24_O_10_) derived from albiflorin. Therefore, we identified M11–M13 as desbenzoyl albiflorin isomers.

According to our previous study (Liang et al., 2013), M14–M15, M16–M17, M23–M28, M35, M36–M40, M63–M64, M65, and M66–M67 were tentatively identified as desbenzoylpaeoniflorin isomers, methyl desbenzoylpaeoniflorin isomers, C_10_H_14_O_3_ sulfate isomers, C_10_H_20_O_4_ glucuronide, C_8_H_8_O_3_ glucuronide isomers, gallic acid sulfates, hippuric acid, and hydroxyhipuric acid, respectively.

According to the literature, M18–M22 were tentatively identified as paeonimetabolin I glucuronide isomers (Wang et al., 2017b; Sun et al., 2018); M29–M34 were tentatively identified as C_10_H_18_O_4_ glucuronide isomers (Liang et al., 2013; Sun et al., 2018), and M68 was tentatively identified as salicylic acid glucuronide (Shen et al., 2015).

### 3.3 Identification of the metabolites of three catechins (CG, ECG, C)

**FIGURE 2:**
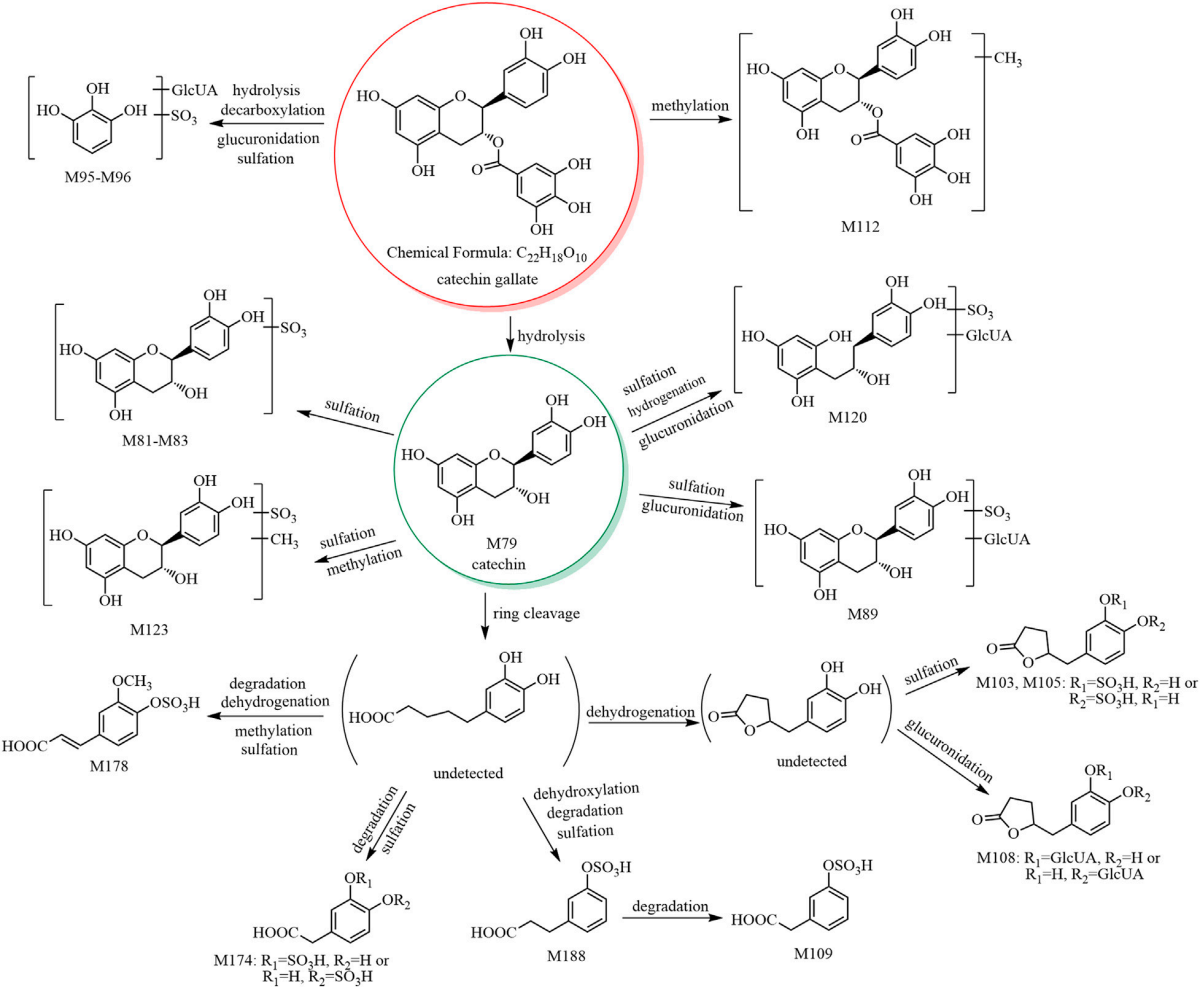
The 17 metabolites (all new) and proposed metabolic pathways of catechin gallate in mice.

The metabolic pathway of catechin gallate is shown in Figure 2, and the metabolic pathways of other catechins are shown in Supplementary Figure S4.

#### 3.3.1 Catechin (C_15_H_14_O_6_) and its phase II metabolites (M79–M91)

M79 showed [M−H]^−^ at *m/z* 289.07, and its molecular formula was predicted as C_15_H_14_O_6_. The retention time and mass spectral data of M79 are consistent with those of catechin’s reference substance (see Table 1 and Supplementary Table S1). Therefore, M79 was identified as catechin.

M80–M85 showed [M−H]^−^ at *m/z* 369.02, and their molecular formula was predicted as C_15_H_14_O_9_S. The fragment ion at *m/z* 289.07 (C_15_H_13_O_6_) was formed by a neutral loss of 79.96 Da in the MS^2^ spectra of M80–M85. Therefore, based on the mass spectral features and the literature (Gonzalez-Manzano et al., 2009; Rodriguez-Mateos et al., 2014), M80–M85 were predicted to be (epi)catechin sulfate isomers. In addition, in the MS^2^ spectra of M81 and M84, the fragment ion at *m/z* 216.97 (C_7_H_5_O_6_S) of the sulfate conjugate of the characteristic fragment ion at *m/z* 137.02 (C_7_H_5_O_3_) formed after the RDA cleavage of the C-ring of catechin. Therefore, we presumed that the sulfate group bound to the hydroxyl group at C-5 or C-7. In the MS^2^ spectra of M85, the ions at *m/z* 289.06 (C_15_H_13_O_6_) and *m/z* 179.03 (C_9_H_7_O_4_) were formed by the sequential losses of 79.96 Da (SO_3_) and 110.04 Da (C_6_H_6_O_2_) from the ion at *m/z* 369.02; however, no neutral loss of 82 Da (H_2_SO_3_) was observed. We concluded that the sulfate group did not bind to the hydroxyl group at C-3 due to the energy barrier required for bond breakage. No fragment ion was observed at *m/z* 216.97 (C_7_H_6_O_6_S), and we concluded that the sulfate group did not bind to the hydroxyl group at C-5 or C-7. Hence, we assumed that the sulfate group bound to the hydroxyl group at C-3′ or C-4'.

M86–M88 showed [M−H]^−^ at *m/z* 465.10, and their molecular formula was predicted as C_21_H_22_O_12_. The ion at *m/z* 289.06 (C_15_H_13_O_6_) was formed by a neutral loss of 176.03 Da (C_6_H_8_O_6_) in the MS^2^ spectra. According to the literature (Gonzalez-Manzano et al., 2009; Liang et al., 2013; Rodriguez-Mateos et al., 2014), M86–M88 were predicted to be catechin glucuronide isomers.

M89–M91 showed [M−H]^−^ at *m/z* 545.06, and their molecular formula was predicted as C_21_H_22_O_15_S. The fragment ions at *m/z* 369.02 (C_15_H_13_O_9_S) and 289.05 (C_15_H_13_O_6_) were formed by the sequential losses of 176.03 Da (C_6_H_8_O_6_) and 79.96 Da (SO_3_) in the MS^2^ spectra. Therefore, M89–M91 were predicted to be catechin glucuronide sulfate isomers.

#### 3.3.2 5-(3,4-Dihydroxyphenyl)-valeric acid (C_11_H_14_O_4_) and its phase II metabolites (M97–M100)

M97 showed [M−H]^−^ at *m/z* 209.08, and its molecular formula was predicted as C_11_H_14_O_4_. The fragment ion at *m/z* 147.07 (C_10_H_11_O) was formed by a neutral loss of 62.01 Da (CO_2_+H_2_O) in the MS^2^ spectrum. According to the literature (Scheline, 1970), M97 was predicted to be 5-(3,4-dihydroxyphenyl)-valeric acid.

M98–M100 showed [M−H]^−^ at *m/z* 289.03, and their molecular formula was predicted as C_11_H_14_O_7_S. The fragment ion at *m/z* 209.07 (C_11_H_13_O_4_) was formed by a neutral loss of 79.96 Da (SO_3_) in the MS^2^ spectra. Therefore, M98–M100 were predicted to be 5-(3,4-dihydroxyphenyl)-valeric acid sulfate isomers.

#### 3.3.3 Phase II metabolites of 5-(3,4-dihydroxyphenyl)-γ-valerolactone (C_11_H_12_O_4_) (M103–M108)

M103–M106 and M107–M108 showed [M−H]^−^ at *m/z* 287.02 and 383.09, respectively, and their molecular formulae were predicted as C_11_H_12_O_7_S and C_17_H_20_O_10_, respectively. The fragment ion at *m/z* 207.05 (C_11_H_11_O_4_) was formed by a neutral loss of 79.96 Da (SO_3_) from the ion at *m/z* 287.02 and a neutral loss of 176.03 Da (C_6_H_8_O_6_) from the ion at *m/z* 383.09 in the MS^2^ spectra. Based on our previous report (Liang et al., 2013) and a report of 5-(3,4-dihydroxyphenyl)-γ-valerolactone (Kohri et al., 2003), we presumed that M103–M106 were 5-(3,4-dihydroxyphenyl)-γ-valerolactone sulfate isomers, while M107–M108 were 5-(3,4-dihydroxyphenyl)-γ-valerolactone glucuronide isomers.

#### 3.3.4 Identification of methyl catechin gallate (M112)

M112 showed [M−H]^−^ at *m/z* 455.09, and its molecular formula was predicted as C_23_H_20_O_10_. The fragment ion at *m/z* 303.08 (C_16_H_15_O_6_) was formed by a neutral loss of 152.01 Da (C_7_H_4_O_4_, galloyl) in the MS^2^ spectrum, and the signal at *m/z* 303.08 indicated an additional methyl group compared to catechin (C_15_H_13_O_6_). Thus, M112 was predicted to having a methylcatechin skeleton. In addition, the fragment ion at *m/z* 169.01 (C_7_H_5_O_5_, gallic acid group) was observed; thus, its structure was presumed to contain a gallic acid group. Comparison of the molecular formula of M112 (C_23_H_20_O_10_) with that of the original constituent (catechin gallate, C_22_H_18_O_10_) suggested that M112 was a methylated product of catechin gallate. According to a previous report, M112 was predicted to be methyl catechin gallate (Kohri et al., 2003).

#### 3.3.5 3,4-DiHPP-2-ol (C_15_H_16_O_6_) and its phase II metabolites (M113–M119, M120)

M113–M119 showed [M−H]^−^ at *m/z* 291.08, and their molecular formula was predicted as C_15_H_16_O_6_. Based on the literature (Hara-Terawaki et al., 2017) and the origins of the metabolites (M113 was derived from catechin, while M114–M119 were derived from PRR decoction), M113 was tentatively identified as 3,4-diHPP-2-ol, while M114–M119 are isomers of M113 and were thus predicted to be 3,4-diHPP-2-ol isomers.

M120 showed [M−H]^−^ at *m/z* 547.14, and its molecular formula was predicted as C_21_H_24_O_15_S. The characteristic ion at *m/z* 371.11 (C_15_H_15_O_9_S) was formed by a neutral loss of 176.03 Da (C_6_H_8_O_6_) in the MS^2^ spectrum. Therefore, M120 was tentatively identified as 3,4-diHPP-2-ol glucuronide sulfate.

#### 3.3.6 Phase II metabolites of methyl catechin (C_16_H_16_O_6_) (M122–M126, M127–M129, and M130–M132)

M122–M126 showed [M−H]^−^ at *m/z* 383.04, and their molecular formula was predicted as C_16_H_16_O_9_S. The ion at *m/z* 303.08 (C_16_H_15_O_6_) of aglycone methylcatechin was formed by a neutral loss of 79.96 Da (SO_3_) in the MS^2^ spectra. In addition, the fragment ions at *m/z* 216.97 (C_7_H_5_O_6_S) and 137.02 (C_7_H_5_O_3_) were observed in all these metabolites except M124; hence, we concluded that the sulfate groups of M122, M123, M125, and M126 bind to the hydroxyl group at C-5 or C-7, and the methylation reaction occurs at the hydroxyl group at C-3, C-3′, or C-4′. According to our previous study (Liang et al., 2013), M122–M126 were predicted to be methyl catechin sulfate isomers.

M127–M129 showed [M−H]^−^ at *m/z* 479.11, and their molecular formula was predicted as C_22_H_24_O_12_. The fragment ion at *m/z* 303.08 ([aglycone−H]^−^, C_16_H_15_O_6_) was formed by a neutral loss of 176.03 Da (C_6_H_8_O_6_) in the MS^2^ spectra. The fragment ion at *m/z* 313.0573 (C_13_H_13_O_9_) was observed in the MS^2^ spectrum of M129, indicating that the glucuronide group binds to the hydroxyl group at C-5 or C-7, and the methylation reaction occurs at the hydroxyl group at C-3, C-3′, or C-4′. According to our previous study (Liang et al., 2013), M127–M129 were predicted to be methyl catechin glucuronide isomers.

M130–M132 showed [M−H]^−^ at *m/z* 559.07, and its molecular formula was predicted as C_22_H_24_O_15_S. The fragment ions at *m/z* 383.04 (C_16_H_15_O_9_S) and 303.08 ([aglycone−H]^−^, C_16_H_15_O_6_) were formed by sequential losses of 176.03 Da (C_6_H_8_O_6_) and 79.96 Da (SO_3_) in the MS^2^ spectra. Therefore, M130–M132 were predicted to be methyl catechin glucuronide sulfate isomers.

#### 3.3.7 Other metabolites of catechins

M92–M94, M95–M96, M101–M102, M109, M110–M111, M121, M133, and M134–M135 showed [M−H]^−^ at *m/z* 355.04, 381.01, 305.03, 273.00, 271.02, 531.08, 242.99, and 216.98, respectively, and their molecular formulae were predicted as C_15_H_16_O_8_S, C_12_H_14_O_12_S, C_11_H_14_O_8_S, C_10_H_10_O_7_S, C_11_H_12_O_6_S, C_21_H_24_O_14_S, C_9_H_8_O_6_S, and C_7_H_6_O_6_, respectively. According to our previous study (Liang et al., 2013), M92–M94, M95–M96, M101–M102, M109, M110–M111, M121, M133, and M134–M135 were predicted to be 3-HPP-2-ol sulfate isomers, pyrogallol-*O*-glucuronide sulfates, trihydroxy benzenepentanoic acid sulfate isomers, ferulic acid sulfate, 5-(3-hydroxyphenyl)-γ-valerolactone sulfate isomers, 3-HPP-2-ol glucuronide sulfate, *m*-coumaric acid sulfate, and 3/4-hydroxy benzoic acid sulfate isomers, respectively.

### 3.4 Metabolites of two ellagic acid compounds (EA and DEA)

**FIGURE 3:**
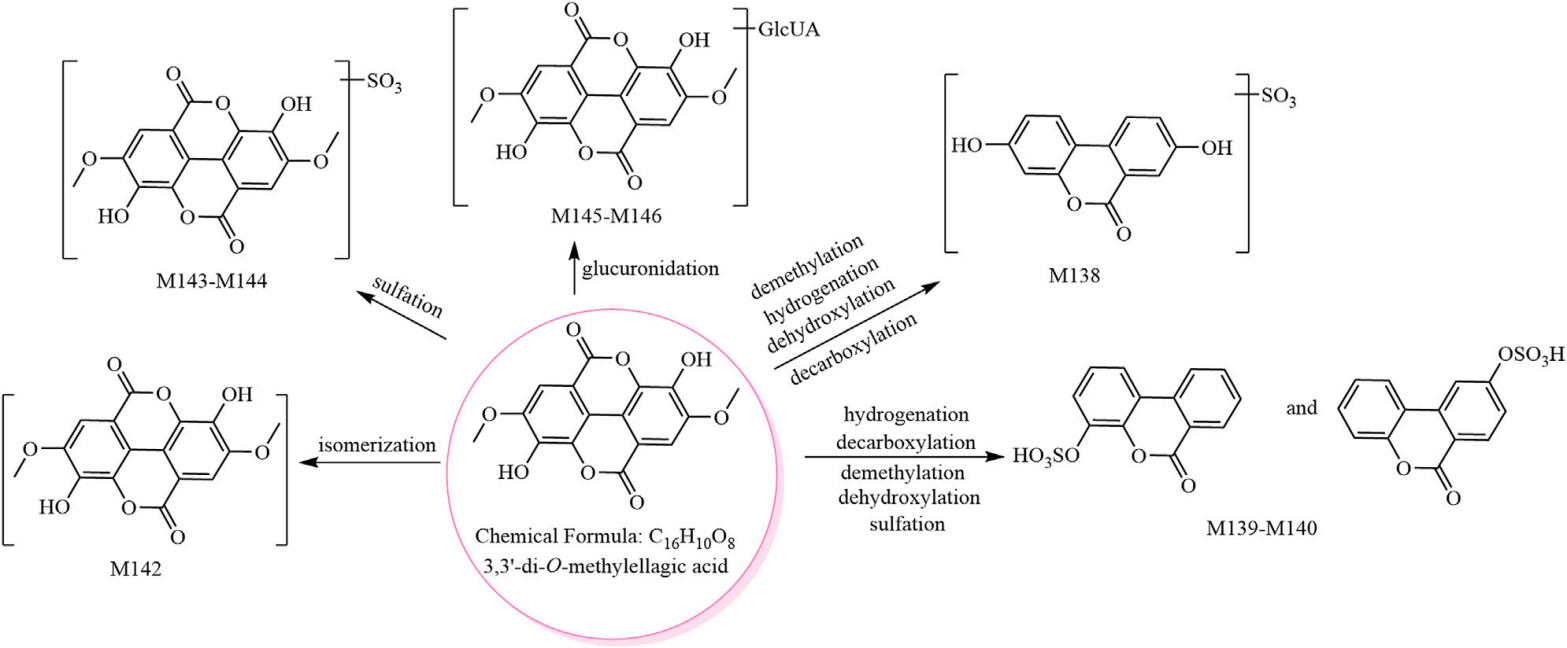
The eight metabolites (all new) and proposed metabolic pathways of 3,3′-di*-O-*methylellagic acid in mice.

The metabolic pathway of 3,3′-di-*O*-methylellagic acid is shown in Figure 3, and the metabolic pathway of ellagic acid is shown in Supplementary Figure S5.

M136 and M137 showed [M−H]^−^ at *m/z* 394.97, and their molecular formula was predicted as C_15_H_8_O_11_S. The fragment ions at *m/z* 315.01 (C_15_H_7_O_8_) and *m/z* 299.98 (C_14_H_4_O_8_, ellagic acid) were formed by sequential losses of 79.96 Da (SO_3_) and 15.02 Da (CH_3_•) in the MS^2^ spectra. Therefore, M136 and M137 were predicted to be methyl ellagic acid sulfate and isomers.

M138 and M139–M141 showed [M−H]^−^ at *m/z* 306.99 and *m/z* 290.99, respectively, and their molecular formulae were predicted as C_13_H_8_O_7_S and C_13_H_8_O_6_S, respectively. M138 was predicted to be urolithin A sulfate (Espin et al., 2013), while M139–M141 were predicted as urolithin B sulfate and isomers (Wang et al., 2017a).

Isomer (C_16_H_10_O_8_, M142) and phase II metabolites (M143–M144, M145–M146) of 3,3′-di*-O-*methylellagic acid were tentatively identified as follows. M142 showed [M−H]^−^ at *m/z* 329.03, and its molecular formula was predicted as C_16_H_10_O_8_. The fragment ions in its MS^2^ spectrum were consistent with those of the 3,3′-di*-O-*methylellagic acid reference compound; however, the retention times were different. M142 was therefore predicted to be an isomer of 3,3′-di*-O-*methylellagic acid. M143–M144 showed [M−H]^−^ at *m/z* 408.98, and their molecular formula was predicted as C_16_H_10_O_11_S. The fragment ion at *m/z* 329.02 ([aglycone−H]^−^, C_16_H_9_O_8_) was formed by a neutral loss of 79.96 Da (SO_3_) in the MS^2^ spectra. The ions at *m/z* 314.00 (C_15_H_6_O_8_) and *m/z* 298.98 (C_14_H_3_O_8_) were consistent with the 3,3′-di*-O-*methylellagic acid reference compound. Thus, M143–M144 were predicted to be 3,3′-di*-O-*methylellagic acid sulfates. M145–M146 showed [M−H]^−^ at *m/z* 505.06, and their molecular formula was predicted as C_22_H_18_O_14_. The fragment ion at *m/z* 329.03 ([aglycone−H]^−^, C_16_H_9_O_8_) was formed by a neutral loss of 176.03 Da (C_6_H_8_O_6_) in the MS^2^ spectra; hence, M145–M146 were predicted to be 3,3′-di*-O-*methylellagic acid glucuronides.

### 3.5 Identification of the metabolites of methyl gallate

**FIGURE 4:**
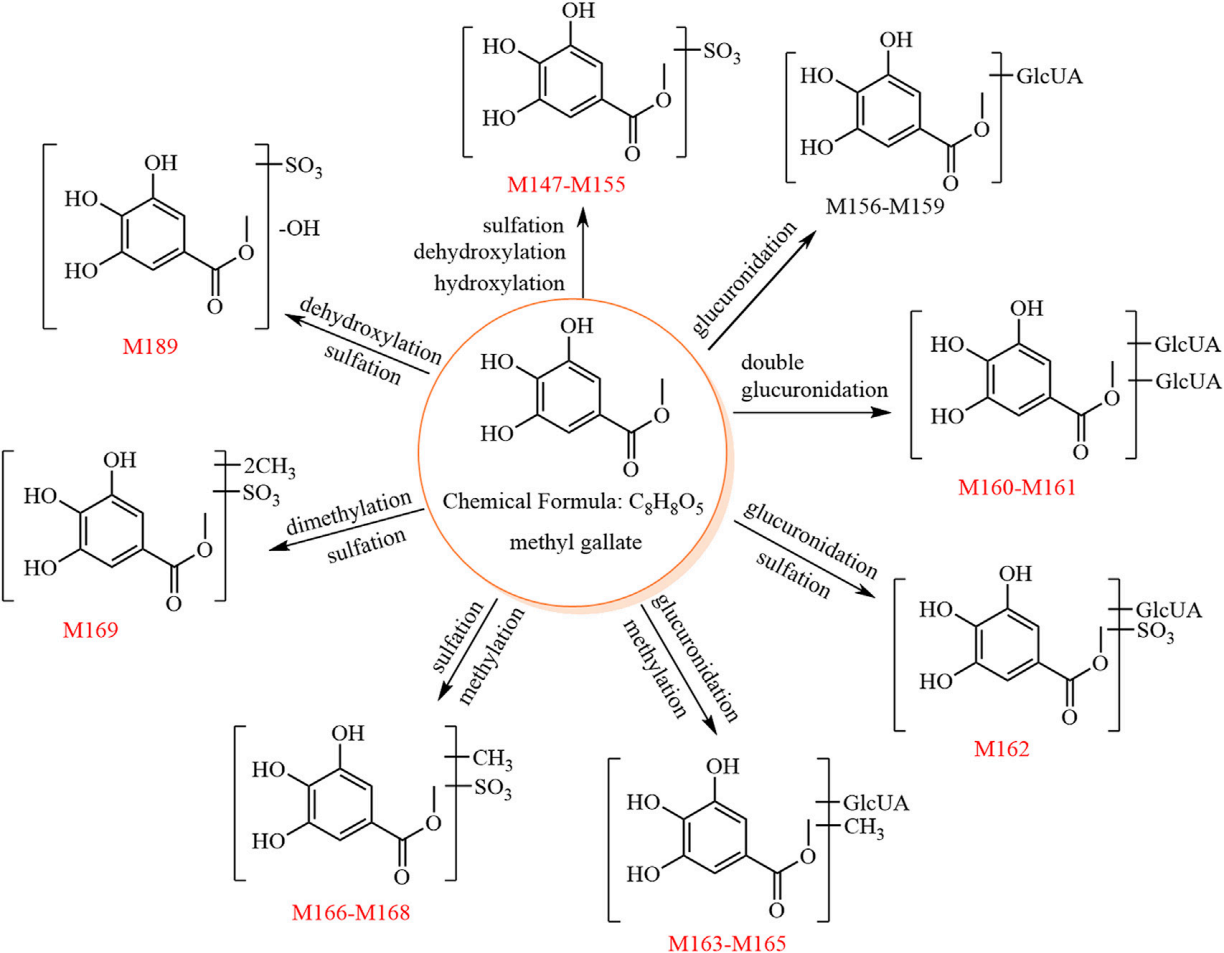
The 24 metabolites and proposed metabolic pathways of methyl gallate in mice. The red numbers denote new metabolites.

The metabolic pathway of methyl gallate is shown in Figure 4.

#### 3.5.1 Phase II metabolites of methyl gallate (C_8_H_8_O_5_) (M147–M155, M156–M159, M160–M161, M162)

M147–M155 showed [M−H]^−^ at *m/z* 262.98, and their molecular formula was predicted as C_8_H_8_O_8_S. The fragment ion at *m/z* 183.02 ([aglycone−H]^−^, C_8_H_7_O_5_) was formed by a neutral loss of 79.96 Da (SO_3_) in the MS^2^ spectra. Due to its chemical structure, methyl gallate is unlikely to produce the metabolites of these nine sulfate conjugates. Thus, we speculated that the aglycones of M147–M155 may be methyl gallate and methylgallic acid, which are isomers. These two isomers show different relative abundances of the characteristic ions at *m/z* 168.00 and *m/z* 124.01; for methyl gallate, the relative abundance of the characteristic ion at *m/z* 124.01 is higher than that at *m/z* 168.00, whereas the opposite is true for methylgallic acid. The MS^2^ spectra of both M147 and M151 showed higher relative abundances of the characteristic ion at *m/z* 124.01 compared to that at *m/z* 168.00; thus, they were predicted to be methyl gallate sulfates. The other metabolites (M148–M150 and M152–M155) were predicted to be methyl gallate sulfate isomers and potentially originated from the metabolic reactions of methyl gallate (e.g., hydroxylation, dehydroxylation, hydrolysis, methylation, and sulfation).

M156–M159 showed [M−H]^−^ at *m/z* 359.06, and their molecular formula was predicted as C_14_H_16_O_11_. The fragment ion at *m/z* 183.02 ([aglycone−H]^−^, C_8_H_7_O_5_) was formed by a neutral loss of 176.03 Da (C_6_H_8_O_6_) in the MS^2^ spectra of M156–M158. Thus, M156–M158 were predicted to be methyl gallate glucuronides. M159 was predicted as a methyl gallate glucuronide isomer since it is an isomer of M156–M158. Because of its chemical structure, methyl gallate is unlikely to produce the four glucuronate conjugates. Thus, we speculated that the aglycones of M156–M159 may be methyl gallate and methylgallic acid. In the MS^2^ spectrum of M157, the relative abundance of the characteristic ion at *m/z* 124.01 was higher than that at *m/z* 168.00; hence, M157 was tentatively identified as methyl gallate glucuronide, whereas M156, M158, and M159 had insufficient characteristic ions and could only be identified as methyl gallate glucuronide isomers.

M160 and M161 showed [M−H]^−^ at *m/z* 535.09, and their molecular formula was predicted as C_20_H_24_O_17_. The fragment ions at *m/z* 359.06 (C_14_H_15_O_11_) and *m/z* 183.02 ([aglycone−H]^−^, C_8_H_7_O_5_) were formed by sequential losses of two 176.03 Da (C_6_H_8_O_6_) in the MS^2^ spectra. Hence, M160–M161 were predicted to be methyl gallate diglucuronides.

M162 showed [M−H]^−^ at *m/z* 439.01, and its molecular formula was predicted as C_14_H_16_O_14_S. The fragment ions at *m/z* 262.97 (C_8_H_7_O_8_S) and *m/z* 183.01 ([aglycone−H]^−^, C_8_H_7_O_5_) were formed by sequential losses of 176.03 Da (C_6_H_8_O_6_) and 79.96 Da (SO_3_) in the MS^2^ spectra. Therefore, M162 was predicted as methyl gallate sulfate glucuronide.

#### 3.5.2 Phase II metabolites of methylated methyl gallate (C_9_H_10_O_5_) (M163–M165, M166–M168)

M163–M165 showed [M−H]^−^ at *m/z* 373.07, and their molecular formula was predicted as C_15_H_18_O_11_. The fragment ion at *m/z* 175.02 (C_6_H_7_O_6_) was observed in the MS^2^ spectra of M163–M165, suggesting the aglycone to be C_9_H_10_O_5_. The fragment ion at *m/z* 183.01 (C_8_H_7_O_5_, methyl gallate) was also observed, suggesting the aglycone to be methylated methyl gallate (C_9_H_10_O_5_).

M166–M168 showed [M−H]^−^ at *m/z* 277.00, and their molecular formula was predicted as C_9_H_10_O_8_S. The fragment ion at *m/z* 197.04 ([aglycone−H]^−^, C_9_H_9_O_5_) was formed by a neutral loss of 79.96 Da (SO_3_) in the MS^2^ spectra.

Therefore, M163–M165 and M166–M168 were predicted as methylated methyl gallate glucuronide isomers and methylated methyl gallate sulfate isomers, respectively.

#### 3.5.3 Phase II metabolite of dimethylated methyl gallate (M169)

M169 showed [M−H]^−^ at *m/z* 291.01, and its molecular formula was predicted as C_10_H_12_O_8_S. The fragment ions at *m/z* 211.06 (C_10_H_11_O_5_) and 196.03 (C_9_H_8_O_5_) were formed by sequential losses of 79.96 Da (SO_3_) and 15.02 Da (CH_3_•) in the MS^2^ spectrum. Therefore, M169 was predicted to be dimethylated methyl gallate sulfate.

### 3.6 Identification of metabolites derived from compounds with different structure types

M171–M176, M177–M180, M181–M186, M187–M191, and M192–M195 showed [M−H]^−^ at *m/z* 230.99, 245.01, 261.00, 246.99, and 319.04, respectively, and their molecular formulae were predicted as C_8_H_8_O_6_S, C_9_H_10_O_6_S, C_9_H_10_O_7_S, C_8_H_8_O_7_S, and C_12_H_16_O_8_S, respectively.

Based on our previous study (Liang et al., 2013), M171–M176, M177–M180, M181–M186, M187–M191, and M192–M195 were predicted as 3/4-hydroxy phenylacetic acid sulfate and its isomers, 3/4-hydroxy phenylpropionic acid sulfate and its isomers, 3,4-dihydroxy phenylpropionic acid sulfate and its isomers, 3,4-dihydroxy phenylacetic acid sulfate and its isomers, and dihydroxylated methoxylated benzenepentanoic acid sulfate and its isomers, respectively.

M171–M176, M177–M180, M181–M186, and M192–M195 are all derived from paeoniflorins and catechins, while M187–M191 are derived from paeoniflorins, catechins, and gallic acids.

### 3.7 Identification of the metabolites of PRR

A total of 31 PRR metabolites were identified (Supplementary Figure S6), the analyses of 30 metabolites were consistent with the above (except M170).

M170 showed [M−H]^−^ at *m/z* 187.00, and its molecular formula was predicted as C_7_H_8_O_4_S. The characteristic fragment ion at *m/z* 107.05 (C_7_H_7_O) was formed by a neutral loss of 79.96 Da (SO_3_) in its MS^2^ spectrum. Based on a previous report (Liu et al., 2020), M170 was tentatively identified as benzyl alcohol sulfate.

## 4 Discussion

### 4.1 Origins of PRR metabolites in mice

A total of 31 metabolites of PRR were identified in mice in this study. The metabolites of 14 PRR constituents were compared, and the relevant literature was analyzed to determine their possible origins. Finally, we identified 13 metabolites derived from catechins (M114–M119, M135, M175–M176, M185–M186, M191, and M195), 14 derived from paeoniflorins (M4–M5, M17, M34, M48–M51, M53, M56, M70, and M72–M74), two derived from ellagic acids (M140–M141), and two derived from other constituents (M65 and M170).

In rats, our previous research (Liang et al., 2013) identified 27, 27, six, 25, and five PRR metabolites derived from catechins, gallic acids, catechins and gallic acids, paeoniflorins, and other constituents, respectively. In comparison, we identified 20 new metabolites formed by hydroxylation, hydrogenation, glucuronidation, and sulfation in mice: seven catechin-related metabolites (M114–M119, 3,4-diHPP-2-ol isomers; and M195, dihydroxylated methoxylated benzenepentanoic acid sulfate); 10 paeoniflorin-related metabolites (M48–M49, 2,6-dihydroxycineol sulfate isomers; M50, M51, and M53, paeonimetabolin II glucoside isomers; M56, hydrogenated 2,6-dihydroxycineol sulfate; M70, hydrogenated lactiflorin isomer; and M72–M74, hydrogenated hydroxylated lactiflorin isomers); two ellagic acid-related metabolites (M140–M141, urolithin B sulfate isomers); and one other metabolite (M170, benzyl alcohol sulfate).

In our previous research (Liang et al., 2013), the administered dosage in male Sprague–Dawley rats was 9.96 g PRR crude drug/kg rat body weight (equivalent to 18.99 g PRR crude drug/kg of mouse body weight). Pre-experiments revealed that the administration of a high dosage of PRR to ICR mice may lead to diarrhea. Therefore, the administered dosage of PRR in this study was 200 mg crude drug/kg mouse body weight, a much lower dosage than that administered in rats. Due to the lower PRR dose used in this study, we did not expect to discover more metabolites of PRR than that previously reported in rats. In addition, significant species differences have been reported in phase I and phase II metabolism (Qin et al., 2021); hence, the 20 new metabolites of PRR identified in mice might be explained by species differences. PRR produces 11 identical metabolites in rats and mice: paeonimetabolin II and its isomers (M4 and M5); C_10_H_18_O_4_ glucuronide (M34); methyl dibenzoylpaeoniflorin isomer (M17); 3/4-hydroxy phenylacetic acid sulfate isomers (M175–M176); 3,4-dihydroxy phenylpropionic acid sulfate isomers (M185–M186); 3,4-dihydroxy phenylacetic acid sulfate (M191); 3/4-hydroxy benzoic acid sulfate isomer (M135); and hippuric acid (M65).

### 4.2 New metabolites found in this study

#### 4.2.1 New metabolites of PRR and its 14 constituents in mice

This was the first study on the *in vivo* metabolism of PRR and its 14 major constituents in mice. Thus, all the metabolites identified were newly found in mice.

#### 4.2.2 New metabolites of PRR and each of its 14 constituents in the whole animal kingdom and microorganism

For 14 constituents of PRR, based on comparison with the literature, the following new metabolites were found in this study: eight new metabolites of paeoniflorin (M42–M47, M52, and M55) (Doyle and Griffiths, 1980; Hattori et al., 1985; Shu et al., 1987; Liang et al., 2013; Cao et al., 2015; Zhu et al., 2018); 11 new metabolites of albiflorin (M7, M9–M10, M36–M39, M45, M47, M55, and M65) (Cao et al., 2015; Wang et al., 2017b); 24 new metabolites of oxypaeoniflorin (Ml, M14–M15, M18–M19, M22, M25–M26, M28–M29, M31, M33, M41–M47, M55, M57–M58, and M60–M61) (Hattori et al., 1985); and 17 new metabolites of benzoylpaeoniflorin (Ml, M14–M15, M18–M19, M23–M24, M27, M31, M33, M43–M45, M47, M57–M58, and M61) (Hattori et al., 1985). No metabolites of hydroxybenzoylpaeoniflorin, benzoyloxypaeoniflorin, galloylpaeoniflorin, or lactiflorin had been reported *in vivo* or *in vitro*. Thus, we identified 18, 30, 27, and 17 new metabolites of hydroxybenzoylpaeoniflorin, benzoyloxypaeoniflorin, galloylpaeoniflorin, or lactiflorin, respectively, in this study.

Considering the literature (Kohri et al., 2003), we found 21 new metabolites of epicatechin gallate: M80, M92–M93, M95, M98, M109, M101, M103–M104, M107, M110, M122, M133, M134, M174, M178, M182–M183, M188, M190, and M193. No metabolites of catechin gallate had yet been reported *in vivo* or *in vitro*. Hence, we identified 17 new metabolites of catechin gallate and one new metabolite of catechin (M121) (Liang et al., 2013).

Considering the literature (Doyle and Griffiths, 1980), we found two new metabolites of ellagic acid (M136 and M137). No *in vivo* or *in vitro* metabolites of 3,3′-di*-O-*methylellagic acid had been reported; hence, we identified eight new metabolites of 3,3′-di*-O-*methylellagic acid in this study.

Considering the literature (Jiamboonsri et al., 2016), we found 20 new metabolites of methyl gallate in this study (M147–M155, M160–M169, and M189).

Considering the literature (Liang et al., 2013), 20 new metabolites (M48–M49, M51–M52, M54, M56, M70, M72–M74, M114–M119, M140–M141, and M170) of PRR decoction were found in this study.

#### 4.2.3 New metabolites of paeoniflorins, catechins, ellagic acids, and gallic acids in the whole animal kingdom and microorganism

A total of 26 new metabolites of paeoniflorin-type compounds were found in this study. Specifically, we found eight new metabolites of paeoniflorin, six new metabolites of albiflorin, 13 new metabolites of oxypaeoniflorin, seven new metabolites of benzoylpaeoniflorin, six new metabolites of hydroxybenzoylpaeoniflorin, seven new metabolites of benzoyloxypaeoniflorin, nine new metabolites of galloylpaeoniflorin, and 12 new metabolites of lactiflorin. The 26 new metabolites were hydrogenated 2,6-dihydroxycineol sulfate isomer (M55), 2,6-dihydroxycineol sulfate isomers (M41–M47), paeonimetabolin II sulfate isomers (M7–M10), paeonimetabolin II glucoside isomers (M52 and M54), hydrogenated lactiflorin and its isomer (M69 and M70), hydrogenated hydroxylated lactiflorin isomer (M71), hydrogenated deglycosylated lactiflorin sulfates (M75–M78), 3/4-hydroxy phenylacetic acid sulfate isomers (M171 and M172), 3/4-hydroxy phenylpropionic acid sulfate (M177), 3,4-dihydroxy phenylpropionic acid sulfate (M181), and 3,4-dihydroxy phenylacetic acid sulfate (M187).

One new metabolite of each of catechin gallate and catechin was found, namely, 3,4-diHPP-2-ol glucuronide sulfate (M120) and 3-HPP-2-ol glucuronide sulfate (M121), respectively.

Eight new metabolites of ellagic acids were found in this study. Specifically, we found two new metabolites of ellagic acid [methylellagic acid sulfates (M136 and M137)] and six new metabolites of 3,3′-di*-O-*methylellagic acid [3,3′-di*-O-*methylellagic acid isomer (M142), 3,3′-di*-O-*methylellagic acid sulfates (M143 and M144), and 3,3′-di*-O-*methylellagic acid glucuronides (M145, and M146)].

Eight new metabolites of gallic acids were found in this study: methyl gallate diglucuronides (M160 and M161); methyl gallate sulfate glucuronide (M162); methylated methyl gallate glucuronides (M163–M165); dimethylated methyl gallate sulfate (M169); and 3, 4-dihydroxy phenylacetic acid sulfate isomer (M189).

### 4.3 Isomer metabolites produced by multiple original constituents of PRR

#### 4.3.1 Isomer metabolites produced by compounds with the same structure type

The eight paeoniflorin constituents all produced desbenzoylpaeoniflorin isomers (M11–M15). Seven paeoniflorin constituents (all except lactiflorin) produced 2,6-dihydroxycineol sulfate isomers (M41–M47). Seven paeoniflorin constituents (all except albiflorin) produced paeonimetabolin I glucuronide isomers (M18–M22) and C_10_H_18_O_4_ glucuronide isomers (M29–M34). Six paeoniflorin constituents (all except albiflorin and lactiflorin) produced C_10_H_14_O_3_ sulfate isomers (M23–M28), hydrogenated 2,6-dihydroxycineol sulfate isomer (M55), and dehydrogenated 2,6-dihydroxycineol glucuronide isomers (M57–M62). Albiflorin, benzoyloxypaeoniflorin, and galloylpaeoniflorin produced C_8_H_8_O_3_ glucuronide isomers (M36–M40) and hippuric acid (M65). Paeoniflorin, benzoyloxypaeoniflorin, and galloylpaeoniflorin produced paeonimetabolin II glucoside isomers (M50–M54). Albiflorin, hydroxybenzoylpaeoniflorin, and galloylpaeoniflorin produced paeonimetabolin II sulfate isomers (M7–M10). Albiflorin, benzoylpaeoniflorin, hydroxybenzoylpaeoniflorin, and galloylpaeoniflorin all produced paeoniflorin (M1). Paeoniflorin, hydroxybenzoylpaeoniflorin, and benzoyloxypaeoniflorin all produced oxypaeoniflorin (M2). Both benzoyloxypaeoniflorin and galloylpaeoniflorin produced paeonimetabolin II isomers (M3 and M6).

All three catechin constituents produced (epi)catechin sulfate isomers (M80–M85), 5-(3,4-dihydroxyphenyl)-γ-valerolactone sulfate isomers (M103–M106), and methyl-catechin sulfates (M122–M126). Both catechin gallate and catechin produced catechin glucuronide sulfates (M89–M91). Both epicatechin gallate and catechin produced 3-HPP-2-ol sulfate isomers (M92–M94), 5-(3,4-dihydroxyphenyl)-valeric acid sulfate isomers (M98–M100), trihydroxy-benzenepentanoic acid sulfate isomers (M101–M102), and 5-(3-hydroxyphenyl)-γ-valerolactone sulfate isomers (M110–M111). Both epicatechin gallate and catechin gallate produced pyrogallol-*O*-glucuronide sulfate isomers (M95–M96) and 5-(3,4-dihydroxyphenyl)-γ-valerolactone glucuronide isomers (M107–M108).

Both ellagic acid constituents produced urolithin B sulfate isomers (M139–M141).

#### 4.3.2 Isomer metabolites produced by compounds with different structure types

Lactiflorin and the three catechin constituents produced 3-hydroxy phenylacetic acid sulfate isomers (M171–M176) and 3/4-hydroxy phenylpropionic acid sulfate isomers (M177–M180).

Lactiflorin, epicatechin gallate, and catechin produced 3,4-dihydroxy phenylpropionic acid sulfate isomers (M181–M186) and dihydroxy-methoxyl-benzenepentanoic acid sulfate isomers (M192–M195).

Lactiflorin, epicatechin gallate, catechin gallate, catechin, methyl gallate, and PRR produced 3,4-dihydroxy phenylacetic acid sulfate isomers (M187–M191).

### 4.4 Origins of the effective forms of PRR

In a previous study, we found 21 effective forms of PRR that account for its effects of clearing away heat, cooling the blood, and dissipating blood stasis (Xu et al., 2022). In this study, we elucidated some possible origins of the 10 known effective forms of PRR and their isomers:

- Desbenzoylpaeoniflorin isomer (C3) can be derived from eight original constituents: paeoniflorin, albiflorin, oxypaeoniflorin, benzoylpaeoniflorin, hydroxybenz oylpaeoniflorin, benzoyloxypaeoniflorin, galloylpae oniflorin, and lactiflorin.
- Paeoniflorin (C1) can be derived from benzoylpaeoniflorin, hydroxybenzoylpaeoniflorin, and galloylpaeoniflorin.
- Oxypaeoniflorin (C2) can be derived from paeoniflorin, hydroxybenzoylpaeoniflorin, and benzoyloxypaeoniflorin.
- 3/4-Hydroxy benzoic acid sulfate (C8) isomer can be derived from epicatechin gallate.
- C_10_H_18_O_4_ glucuronide (C16) isomer can be derived from seven prototype paeoniflorin constituents (all except albiflorin).
- C_8_H_8_O_3_ glucuronide isomers (C19 and C20) can be derived from albiflorin, benzoyloxypaeoniflorin, and galloylpa eoniflorin.
- 3′*-O-*methyl (epi)catechin 5*-O-*glucuronide (C5) isomer can be derived from catechin.
- 3-Hydroxy phenylpropionic acid sulfate (C6) isomer can be derived from lactiflorin, epicatechin gallate, catechin gallate, and catechin.
- 3,3′-Di*-O-*methylellagic acid isomer can be isomerized from 3,3′-di*-O-*methylellagic acid.

According to our previous study (Liang et al., 2013), C_10_H_18_O_2_ glucuronides (C9–C15) and C_10_H_14_O_3_ glucuronide (C17), two effective forms of PRR, can be derived from paeoniflorin in rats; however, their origins could not be determined in this study. Other effective forms of PRR including 3-hydroxy-4-methoxy-phenylpropionic acid sulfate (C7), 3-methoxy-4-hydroxy-phenylpropionic acid sulfate (C18), and benzoyl glucuronide (C21) can be derived from catechins in rats (Liang et al., 2013), but their specific origins need further investigation.

### 4.5 Insights for the quality control of PRR

Seventeen of the effective forms of PRR are metabolites that are not present in PRR and cannot be used for quality control. This study identified some of the precursors of the 10 effective forms of PRR, which can be used as indicators for the quality control of PRR.

## 5 Conclusion

This was the first study on the *in vivo* metabolism of PRR and its 14 constituents in mice. The metabolites were identified by the HPLC-DAD-ESI-IT-TOF-MS^n^. In total, we identified 23, 16, 24, 17, 18, 30, 27, 17, 22, 17, 33, 3, 8, 24, and 31 metabolites of paeoniflorin, albiflorin, oxypaeoniflorin, benzoylpaeoniflorin, hydroxybenzoylpaeoniflorin, benzoyloxypaeoniflorin, galloylpaeoniflorin, lactiflorin, epicatechin gallate, catechin gallate, catechin, ellagic acid, 3,3′-di*-O-*methylellagic acid, methylgallate, and PRR, respectively, in mice. The main metabolic reactions included methylation, hydrogenation, hydrolysis, hydroxylation, glucuronidation, and sulfation. We elucidated the metabolites and metabolic pathways of the 14 constituents of PRR, including paeoniflorins, catechins, ellagic acids, and gallic acids, in mice, and clarified the possible origins of the 10 known effective forms of PRR and their isomers. The findings will facilitate further studies on the effective forms of PRR *in vivo* and are of great significance for exploring the mechanisms of action and quality control of PRR.

## Data Availability

The original contributions presented in the study are included in the article/Supplementary Material, further inquiries can be directed to the corresponding authors.

## Abbreviations

A, albiflorin; B, benzoylpaeoniflorin; BO, benzoyloxypaeoniflorin; BPCs, base peak chromatograms; C, catechin; CDL, curved desolvation line; CG, catechin gallate; CID, relative collision-induced dissociation; d, day; DEA, 3,3′-di-*O*-methylellagic acid; EA, ellagic acid; ECG, epicatechin gallate; EICs, extracted ion chromatograms; Err., error.; ESI, electrospray ionization; G, galloylpaeoniflorin; HPLC, high-performance liquid chromatography; HPLC-ESI-IT-TOF-MS^n^, high performance liquid chromatography with diode array detector and combined with electrospray ionization ion trap time-of-flight multistage mass spectrometry; L, lactiflorin; Meas., measured; MG, methyl gallate; NLRP3, NOD-like receptor thermal protein domain associated protein 3; O, oxypaeoniflorin; OB, hydroxybenzoylpaeoniflorin; P, paeoniflorin; PRR. Paeoniae Radix Rubra; t_R_, retention time.
