# Supplementary material for: Metabolism of Paeoniae Radix Rubra and its 14 constituents in mice
Source: Front Pharmacol. 2022 Oct 4;13:995641. doi: 10.3389/fphar.2022.995641 (PMC9577399; doi:10.3389/fphar.2022.995641)
Supplement: Supplementary file 1 [file Table1.DOCX]

**Supplementary material**

**Table S1.** The LC/MS data for identification of 10 absorbed compounds and 195 metabolites in mice. P, paeoniflorin; A, albiflorin; O, oxypaeoniflorin; B, benzoylpaeoniflorin; OB, hydroxybenzoylpaeoniflorin; BO, benzoyloxypaeoniflorin; G, galloylpaeoniflorin; L, lactiflorin; ECG, epicatechin gallate; CG, catechin gallate; C, catechin; EA, ellagic acid; DEA, 3,3'-di-*O*-methylellagic acid; MG, methyl gallate; PRR, Paeoniae Radix Rubra. t_R_, retention time; Meas., measured; Err., error.; ^a^, identified by comparison with reference compounds.

| No. | t_R_(min) | Formula | Meas. (Da) | Err.  (ppm) | Identification | Major fragment ions (Negative Ion) |
| --- | --- | --- | --- | --- | --- | --- |
| P0^a^ | 56.58 | C_23_H_28_O_11_ | 525.1590 | −4.57 | paeoniflorin | 479.1582, 449.1472, 431.1344, 327.1092, 309.0955, 297.1030, 283.1022 |
| A0^a^ | 51.95 | C_23_H_28_O_11_ | 525.1597 | −3.24 | albiflorin | 479.1493, 435.1675, 357.1133, 327.1014, 283.0730, 195.0598 |
| O0^a^ | 42.99 | C_23_H_28_O_12_ | 495.1501 | −1.41 | oxypaeoniflorin | 465.1351, 345.1175, 333.0923, 327.1014, 315.0791, 309.0940, 299.0745, 281.0528 |
| OB0^a^ | 84.65 | C_30_H_32_O_13_ | 599.1726 | −7.34 | hydroxybenzoylpaeoniflorin | 551.1474, 477.1316, 459.1221, 429.1115, 385.0878, 343.0689, 315.0749, 281.0600, 239.0495 |
| BO0^a^ | 87.12 | C_30_H_32_O_13_ | 599.1755 | −2.5 | benzoyloxypaeoniflorin | 569.1600, 551.1550, 477.1338, 447.1241, 403.1007, 281.0686 |
| G0^a^ | 70.17 | C_30_H_32_O_15_ | 631.1633 | −5.55 | galloylpaeoniflorin | 613.1485, 583.1420, 509.1336, 493.1222, 491.1143, 463.1217, 399.0893, 331.0673, 313.0504, 271.0402 |
| L0^a^ | 81.32 | C_23_H_26_O_10_ | 507.1508 | 0.00 | lactiflorin | 461.1390, 339.1030, 283.0732, 281.0733, 231.0481 |
| ECG0^a^ | 67.80 | C_22_H_18_O_10_ | 441.0827 | 0.00 | epicatechin gallate | 441.0827, 331.0378, 289.0686, 245.0802 |
| CG0^a^ | 70.36 | C_22_H_18_O_10_ | 441.0828 | 0.23 | catechin gallate | 289.0680, 271.0576, 245.0793, 227.0684, 193.0157, 169.0128 |
| DEA0^a^ | 90.97 | C_16_H_10_O_8_ | 329.0315 | 3.65 | 3,3'-di*-O-*methylellagic acid | 314.0019, 298.9760, 285.0071, 270.9877 |
| M1^a^ | 56.58 | C_23_H_28_O_11_ | 525.1590 | −4.57 | paeoniflorin | 479.1582, 449.1472, 431.1344, 327.1092, 309.0955, 297.1030, 283.1022 |
| M2^a^ | 42.53 | C_23_H_28_O_12_ | 495.1502 | −1.21 | oxypaeoniflorin | 465.1351, 345.1175, 333.0923, 327.1014, 315.0791, 309.0940, 299.0745, 281.0528 |
| M3 | 77.90 | C_10_H_16_O_4_ | 199.0987 | 5.52 | paeonimetabolin II isomer 1 | 155.1020 |
| M4 | 78.72 | C_10_H_16_O_4_ | 199.0978 | 1.00 | paeonimetabolin II isomer 2 | 155.1048 |
| M5 | 80.91 | C_10_H_16_O_4_ | 199.0980 | 2.01 | paeonimetabolin II isomer 3 | 154.9869 |
| M6 | 80.24 | C_10_H_16_O_4_ | 199.0970 | −3.01 | paeonimetabolin II isomer 4 | 155.1086 |
| M7 | 23.69 | C_10_H_16_O_7_S | 279.0532 | −4.30 | paeonimetabolin II sulfate isomer 1 | 96.9627 |
| M8 | 31.89 | C_10_H_16_O_7_S | 279.0525 | −6.81 | paeonimetabolin II sulfate isomer 2 | 159.0194 |
| M9 | 43.84 | C_10_H_16_O_7_S | 279.0524 | −7.17 | paeonimetabolin II sulfate isomer 3 | 96.9657 |
| M10 | 49.22 | C_10_H_16_O_7_S | 279.0523 | −7.53 | paeonimetabolin II sulfate isomer 4 | 159.0075, 121.1478, 96.9684 |
| M11 | 8.77 | C_16_H_24_O_10_ | 421.1361 | 2.14 | desbenzoyl albiflorin isomer 1 | 391.0284, 375.1311, 345.1077 |
| M12 | 31.28 | C_16_H_24_O_10_ | 375.1266 | −8.26 | desbenzoyl albiflorin isomer 2 | 315.1149 |
| M13 | 10.08 | C_16_ H_24_O_10_ | 421.1332 | −4.75 | desbenzoyl albiflorin isomer 3 | 375.1303, 345.1075, 213.0806, 165.0643 |
| M14 | 51.34 | C_16_H_24_O_10_ | 421.1333 | −4.51 | desbenzoylpaeoniflorin isomer 1 | 375.1218, 255.0840, 240.0585 |
| M15 | 52.97 | C_16_H_24_O_10_ | 421.1346 | −1.42 | desbenzoylpaeoniflorin isomer 2 | 375.1275 |
| M16 | 29.71 | C_17_H_26_O_10_ | 435.1485 | −5.29 | methyl debenzoylpaeoniflorin isomer 1 | 389.1529, 227.0840 |
| M17 | 26.15 | C_17_H_26_O_10_ | 435.1477 | −7.12 | methyl debenzoylpaeoniflorin isomer 2 | 389.1326 |
| M18 | 32.07 | C_16_H_22_O_10_ | 373.1146 | 1.61 | paeonimetabolin I glucuronide isomer 1 | 175.0286, 157.0191 |
| M19 | 33.01 | C_16_H_22_O_10_ | 373.1145 | 1.34 | paeonimetabolin I glucuronide isomer 2 | 193.0324, 175.0341 |
| M20 | 29.78 | C_16_H_22_O_10_ | 373.1133 | −1.88 | paeonimetabolin I glucuronide isomer 3 | 165.0598 |
| M21 | 28.88 | C_16_H_22_O_10_ | 373.1123 | −4.56 | paeonimetabolin I glucuronide isomer 4 | 313.0743, 167.0755 |
| M22 | 55.09 | C_16_H_22_O_10_ | 373.1135 | −1.34 | paeonimetabolin I glucuronide isomer 5 | 203.0336 |
| M23 | 24.88 | C_10_H_14_O_6_S | 261.0437 | −0.38 | C_10_H_14_O_3_ sulfate isomer 1 | 179.0623, 137.0637 |
| M24 | 26.01 | C_10_H_14_O_6_S | 261.0438 | 0.00 | C_10_H_14_O_3_ sulfate isomer 2 | 137.0698 |
| M25 | 28.75 | C_10_H_14_O_6_S | 261.0423 | −5.75 | C_10_H_14_O_3_ sulfate isomer 3 | 179.0844, 137.0575 |
| M26 | 29.29 | C_10_H_14_O_6_S | 261.0423 | −5.75 | C_10_H_14_O_3_ sulfate isomer 4 | 179.0591, 137.0583 |
| M27 | 34.56 | C_10_H_14_O_6_S | 261.0429 | −3.45 | C_10_H_14_O_3_ sulfate isomer 5 | 137.0600, 122.0378 |
| M28 | 37.82 | C_10_H_14_O_6_S | 261.0432 | −2.3 | C_10_H_14_O_3_ sulfate isomer 6 | 137.0575 |
| M29 | 24.33 | C_16_H_26_O_10_ | 377.1475 | 5.83 | C_10_H_18_O_4_ glucuronide isomer 1 | 359.1332, 317.0639, 299.1054 |
| M30 | 24.90 | C_16_H_26_O_10_ | 377.1443 | −2.65 | C_10_H_18_O_4_ glucuronide isomer 2 | 359.1332, 139.0398 |
| M31 | 26.71 | C_16_H_26_O_10_ | 377.1463 | 2.65 | C_10_H_18_O_4_ glucuronide isomer 3 | 359.1316, 341.1278 |
| M32 | 30.81 | C_16_H_26_O_10_ | 377.1453 | 0.00 | C_10_H_18_O_4_ glucuronide isomer 4 | 359.1691, 312.9569 |
| M33 | 31.97 | C_16_H_26_O_10_ | 377.1437 | −4.24 | C_10_H_18_O_4_ glucuronide isomer 5 | 359.1332 |
| M34 | 53.31 | C_16_H_26_O_10_ | 377.1443 | −2.65 | C_10_H_18_O_4_ glucuronide isomer 6 | 359.1355, 345.0423 |
| M35 | 56.90 | C_16_H_28_O_10_ | 379.1614 | 1.05 | C_10_H_20_O_4_ glucuronide | 171.0906, 153.0985 |
| M36 | 23.65 | C_14_H_16_O_9_ | 327.0731 | 2.75 | C_8_H_8_O_3_ glucuronide isomer 1 | 309.0523, 213.0862 |
| M37 | 31.11 | C_14_H_16_O_9_ | 327.0712 | −3.06 | C_8_H_8_O_3_ glucuronide isomer 2 | 309.0475, 213.0735 |
| M38 | 32.32 | C_14_H_16_O_9_ | 327.0722 | 0.00 | C_8_H_8_O_3_ glucuronide isomer 3 | 213.0708, 135.0468, 125.0889 |
| M39 | 35.98 | C_14_H_16_O_9_ | 327.0722 | 0.00 | C_8_H_8_O_3_ glucuronide isomer 4 | 309.0594, 213.0806, 195.0743, 151.0626 |
| M40 | 41.65 | C_14_H_16_O_9_ | 327.0696 | −7.95 | C_8_H_8_O_3_ glucuronide isomer 5 | 309.0850, 195.0686 |
| M41 | 34.25 | C_10_H_18_O_6_S | 265.0730 | −7.92 | 2,6-dihydroxycineol sulfate isomer 1 | 183.0272, 137.0278 |
| M42 | 47.90 | C_10_H_18_O_6_S | 265.0732 | −7.17 | 2,6-dihydroxycineol sulfate isomer 2 | 251.0690 |
| M43 | 51.20 | C_10_H_18_O_6_S | 265.0736 | −5.66 | 2,6-dihydroxycineol sulfate isomer 3 | 251.0611, 182.0755, 165.0563, |
| M44 | 54.17 | C_10_H_18_O_6_S | 265.0745 | −2.26 | 2,6-dihydroxycineol sulfate isomer 4 | 191.1320 |
| M45 | 63.27 | C_10_H_18_O_6_S | 265.0729 | −8.30 | 2,6-dihydroxycineol sulfate | 183.0973 |
| M46 | 83.48 | C_10_H_18_O_6_S | 265.0739 | −4.53 | 2,6-dihydroxycineol sulfate isomer 6 | 247.0596 |
| M47 | 85.97 | C_10_H_18_O_6_S | 265.0733 | −6.79 | 2,6-dihydroxycineol sulfate isomer 7 | 251.0507 |
| M48 | 111.38 | C_10_H_18_O_6_S | 265.0740 | −4.15 | 2,6-dihydroxycineol sulfate isomer 8 | 237.1073 |
| M49 | 128.78 | C_10_H_18_O_6_S | 265.0753 | 0.75 | 2,6-dihydroxycineol sulfate isomer 9 | 237.0228 |
| M50 | 69.08 | C_16_H_26_O_9_ | 361.1522 | 4.98 | paeonimetabolin II glucoside isomer 1 | 333.0955 |
| M51 | 71.20 | C_16_H_26_O_9_ | 361.1520 | 4.43 | paeonimetabolin II glucoside isomer 2 | 331.1307 |
| M52 | 70.28 | C_16_H_26_O_9_ | 361.1486 | −4.98 | paeonimetabolin II glucoside isomer 3 | 345.1655, 325.0543 |
| M53 | 72.81 | C_16_H_26_O_9_ | 361.1508 | 1.11 | paeonimetabolin II glucoside isomer 4 | 345.122, 333.0955 |
| M54 | 66.15 | C_16_H_26_O_9_ | 361.1489 | −4.15 | paeonimetabolin II glucoside | 199.1054, 155.1072 |
| M55 | 61.87 | C_10_H_20_O_6_S | 267.0936 | 0.00 | hydrogenated 2,6-dihydroxycineol sulfate isomer 1 | 172.9901 |
| M56 | 57.88 | C_10_H_20_O_6_S | 267.0901 | −2.62 | hydrogenated 2,6-dihydroxycineol sulfate isomer 2 | 169.0994 |
| M57 | 38.30 | C_16_H_24_O_9_ | 359.1318 | −8.35 | dehydrogenated 2,6-dihydroxycineol glucuronide isomer 1 | 261.1426, 259.0684, 157.0158 |
| M58 | 39.01 | C_16_H_24_O_9_ | 359.1343 | −1.39 | dehydrogenated 2,6-dihydroxycineol glucuronide isomer 2 | 261.1238 |
| M59 | 39.61 | C_16_H_24_O_9_ | 359.1342 | −1.67 | dehydrogenated 2,6-dihydroxycineol glucuronide isomer 3 | 344.0731, 331.1148 |
| M60 | 41.87 | C_16_H_24_O_9_ | 359.1322 | −7.24 | dehydrogenated 2,6-dihydroxycineol glucuronide isomer 4 | 344.0769, 343.1861 |
| M61 | 48.65 | C_16_H_24_O_9_ | 359.1333 | −4.18 | dehydrogenated 2,6-dihydroxycineol glucuronide | 267.1735, 257.0693, 243.0237, 195.0989, 183.1045, 161.0530 |
| M62 | 49.63 | C_16_H_24_O_9_ | 359.1353 | 1.39 | dehydrogenated 2,6-dihydroxycineol glucuronide isomer 5 | 329.0777 |
| M63 | 32.99 | C_7_H_6_O_8_S | 248.9691 | 5.62 | gallic acid sulfate | 204.9659, 169.0142, 125.0252 |
| M64 | 42.42 | C_7_H_6_O_8_S | 248.9719 | 3.21 | gallic acid sulfate | 169.0101, 125.0252 |
| M65 | 37.17 | C_9_H_9_NO_3_ | 178.0516 | 3.37 | hippuric acid | 134.0607 |
| M66 | 27.64 | C_9_H_9_NO_4_ | 194.0469 | 5.15 | hydroxyhippuric acid | 132.0817, 100.0031 |
| M67 | 29.07 | C_9_H_9_NO_4_ | 194.0459 | 0.00 | hydroxyhippuric acid | 124.0119, 106.9818 |
| M68 | 28.31 | C_13_H_14_O_9_ | 313.0547 | −5.75 | salicylic acid glucuronide | 175.0280, 137.0256 |
| M69 | 71.03 | C_23_H_28_O_10_ | 509.1654 | −2.16 | hydrogenated lactiflorin | 463.1602, 359.1513 |
| M70 | 63.56 | C_23_H_28_O_10_ | 509.1652 | −2.55 | hydrogenated lactiflorin isomer | 463.1447 |
| M71 | 71.64 | C_23_H_28_O_11_ | 525.1598 | −3.05 | hydrogenated hydroxylated lactiflorin | 479.1370, 461.1306, 357.1042, 339.0981 |
| M72 | 35.33 | C_23_H_28_O_11_ | 525.1635 | 4.00 | hydrogenated hydroxylated lactiflorin isomer 1 | 359.1356 |
| M73 | 33.67 | C_23_H_28_O_11_ | 525.1624 | 1.90 | hydrogenated hydroxylated lactiflorin isomer 2 | 447.1619, 359.1270, 329.1163, 311.0956 |
| M74 | 37.32 | C_23_H_28_O_11_ | 525.1624 | 1.90 | hydrogenated hydroxylated lactiflorin isomer 3 | 377.1293, 233.0844 |
| M75 | 85.18 | C_17_H_18_O_8_S | 381.0612 | −1.05 | hydrogenated deglycosylated lactiflorin sulfate isomer 1 | 147.0425 |
| M76 | 66.53 | C_17_H_18_O_8_S | 381.0625 | 2.36 | hydrogenated deglycosylated lactiflorin sulfate isomer 2 | 259.0176, 177.0574, 165.0503, 147.0482 |
| M77 | 84.31 | C_17_H_18_O_8_S | 381.0622 | 1.57 | hydrogenated deglycosylated lactiflorin sulfate isomer 3 | 259.0175, 195.0624, 177.0369, 165.0476, 147.0418 |
| M78 | 100.37 | C_17_H_18_O_8_S | 381.0629 | 3.41 | hydrogenated deglycosylated lactiflorin sulfate isomer 4 | 259.0112, 195.0558, 177.0436, 165.0502, 147.0375 |
| M79^a^ | 40.07 | C_15_H_14_O_6_ | 289.0706 | −4.15 | catechin | 245.0729, 203.0745 |
| M80 | 71.09 | C_15_H_14_O_9_S | 369.0295 | 2.44 | epicatechin sulfate | 203.0730 |
| M81 | 66.12 | C_15_H_14_O_9_S | 369.0285 | −0.27 | catechin 5/7*-O-*sulfate isomer 1 | 289.0736, 245.0821, 216.9849, 137.0279 |
| M82 | 67.48 | C_15_H_14_O_9_S | 369.0268 | −4.88 | catechin sulfate isomer 2 | 289.0751, 247.0433, 230.9961 203.0745 |
| M83 | 72.48 | C_15_H_14_O_9_S | 369.0280 | −1.63 | catechin sulfate isomer 3 | 289.0727, 271.0477, 245.0753, 230.9991 |
| M84 | 61.79 | C_15_H_14_O_9_S | 369.0283 | −0.81 | catechin 5/7*-O-*sulfate isomer 2 | 216.9772 |
| M85 | 68.11 | C_15_H_14_O_9_S | 369.0283 | −0.81 | catechin 3'/4'*-O-*sulfate isomer | 289.0673, 179.0354 |
| M86 | 37.64 | C_21_H_22_O_12_ | 465.1038 | 0.00 | catechin glucuronide isomer 1 | 289.0695, 245.0781, 203.0712,179.0607 |
| M87 | 36.14 | C_21_H_22_O_12_ | 465.1008 | −6.45 | catechin glucuronide isomer 2 | 289.0639, 245.0727 |
| M88 | 32.94 | C_21_H_22_O_12_ | 465.1053 | 3.23 | catechin glucuronide isomer 3 | 289.0688, 245.0665, 205.0493 |
| M89 | 66.43 | C_21_H_22_O_15_S | 545.0599 | −1.47 | catechin glucuronide sulfate isomer 1 | 465.1044, 369.0276, 289.0764, 230.9906 |
| M90 | 61.43 | C_21_H_22_O_15_S | 545.0576 | −5.69 | catechin glucuronide sulfate isomer 2 | 369.0220, 289.0512, 230.9902 |
| M91 | 54.85 | C_21_H_22_O_15_S | 545.0621 | 2.57 | catechin glucuronide sulfate isomer 3 | 369.0244, 289.0658 |
| M92 | 83.80 | C_15_H_16_O_8_S | 355.0485 | −2.25 | 3-HPP-2-ol sulfate isomer 1 | 275.0826 |
| M93 | 85.03 | C_15_H_16_O_8_S | 355.0475 | −5.07 | 3-HPP-2-ol sulfate isomer 2 | 275.0884 |
| M94 | 79.52 | C_15_H_16_O_8_S | 355.0473 | −5.63 | 3-HPP-2-ol sulfate isomer 3 | 275.0840 |
| M95 | 40.82 | C_12_H_14_O_12_S | 381.0138 | 1.31 | pyrogallol-*O*-glucuronide sulfate isomer 1 | 301.0526, 204.9833 |
| M96 | 41.84 | C_12_H_14_O_12_S | 381.0149 | 4.20 | pyrogallol-*O*-glucuronide sulfate isomer 2 | 301.0539, 204.9821, 125.0273 |
| M97 | 50.13 | C_11_H_14_O_4_ | 209.0812 | −3.35 | 5-(3,4-dihydroxyphenyl)-valeric acid | 147.0705 |
| M98 | 69.82 | C_11_H_14_O_7_S | 289.0386 | −0.35 | 5-(3,4-dihydroxyphenyl)-valeric acid sulfate isomer 1 | 289.0386, 209.0813, 147.0796 |
| M99 | 125.97 | C_11_H_14_O_7_S | 289.0400 | 4.50 | 5-(3,4-dihydroxyphenyl)-valeric acid sulfate isomer 2 | 209.0763 |
| M100 | 66.80 | C_11_H_14_O_7_S | 289.0368 | −6.57 | 5-(3,4-dihydroxyphenyl)-valeric acid sulfate isomer 3 | 209.0759, 147.0941 |
| M101 | 64.15 | C_11_H_14_O_8_S | 305.0348 | 3.61 | trihydroxy benzenepentanoic acid sulfate isomer 1 | 225.0806, 207.0721, 163.0699, 145.0583, 123.0515 |
| M102 | 60.56 | C_11_H_14_O_8_S | 305.0316 | −6.88 | trihydroxy benzenepentanoic acid sulfate isomer 2 | 225.0597, 207.0616, 163.0535 |
| M103 | 79.45 | C_11_H_12_O_7_S | 287.0251 | 2.44 | 5-(3,4-dihydroxyphenyl)-γ-valerolactone sulfate isomer 1 | 207.0597 |
| M104 | 72.89 | C_11_H_12_O_7_S | 287.0243 | 4.18 | 5-(3,4-dihydroxyphenyl)-γ-valerolactone sulfate isomer 2 | 207.0779 |
| M105 | 77.34 | C_11_H_12_O_7_S | 287.0227 | −1.39 | 5-(3,4-dihydroxyphenyl)-γ-valerolactone sulfate isomer 3 | 207.0593, 163.0717 |
| M106 | 76.08 | C_11_H_12_O_7_S | 287.0219 | −4.18 | 5-(3,4-dihydroxyphenyl)-γ-valerolactone sulfate isomer 4 | 207.0609, 163.0717 |
| M107 | 46.82 | C_17_H_20_O_10_ | 383.1002 | 4.70 | 5-(3,4-dihydroxyphenyl)-γ-valerolactone glucuronide isomer 1 | 207.0643 |
| M108 | 47.38 | C_17_H_20_O_10_ | 383.1005 | 5.48 | 5-(3,4-dihydroxyphenyl)-γ-valerolactone glucuronide isomer 2 | 207.0629 |
| M109 | 76.97 | C_10_H_10_O_7_S | 273.0072 | −0.73 | ferulic acid sulfate | 193.0434 |
| M110 | 87.39 | C_11_H_12_O_6_S | 271.0286 | 1.48 | 5-(3-hydroxyphenyl)-γ-valerolactone sulfate isomer 1 | 191.0726, 147.1075 |
| M111 | 82.81 | C_11_H_12_O_6_S | 271.0284 | 0.74 | 5-(3-hydroxyphenyl)-γ-valerolactone sulfate isomer 2 | 191.0552 |
| M112 | 78.53 | C_23_H_20_O_10_ | 455.0985 | 0.22 | methyl catechin gallate | 303.0829, 285.0791, 169.0139 |
| M113 | 99.94 | C_15_H_16_O_6_ | 291.0891 | 5.84 | 3,4-diHPP-2-ol | 247.0872 |
| M114 | 95.61 | C_15_H_16_O_6_ | 291.0866 | −2.75 | 3,4-diHPP-2-ol isomer 1 | 247.0889, 211.0404 |
| M115 | 98.62 | C_15_H_16_O_6_ | 291.0875 | 0.34 | 3,4-diHPP-2-ol isomer 2 | 247.0889 |
| M116 | 102.43 | C_15_H_16_O_6_ | 291.0863 | −3.78 | 3,4-diHPP-2-ol isomer 3 | 247.0889 |
| M117 | 108.16 | C_15_H_16_O_6_ | 291.0885 | 3.78 | 3,4-diHPP-2-ol isomer 4 | 291.0898 |
| M118 | 110.64 | C_15_H_16_O_6_ | 291.0880 | 2.06 | 3,4-diHPP-2-ol isomer 5 | 275.0938 |
| M119 | 125.68 | C_15_H_16_O_6_ | 291.0882 | 2.75 | 3,4-diHPP-2-ol isomer 6 | 247.1023, 211.0345, 191.0801 |
| M120 | 95.25 | C_21_H_24_O_15_S | 547.1477 | 3.66 | 3,4-diHPP-2-ol glucuronide sulfate | 371.1193, 233.0744 |
| M121 | 67.91 | C_21_H_24_O_14_S | 531.0796 | −3.39 | 3-HPP-2-ol glucuronide sulfate | 451.1194, 355.0521, 275.0851, 218.0249 |
| M122 | 85.09 | C_16_H_16_O_9_S | 383.0449 | 1.83 | methyl catechin sulfate isomer 1 | 303.0763, 217.0740, 137.0582 |
| M123 | 82.21 | C_16_H_16_O_9_S | 383.0456 | 3.65 | methyl catechin sulfate isomer 2 | 303.0912, 285.0722, 219.0618, 216.9640, 137.0149 |
| M124 | 81.83 | C_16_H_16_O_9_S | 383.0454 | 3.13 | methyl catechin sulfate isomer 3 | 303.0888 |
| M125 | 75.73 | C_16_H_16_O_9_S | 383.0458 | 4.18 | methyl catechin sulfate isomer 4 | 303.0909, 216.9713, 137.0255 |
| M126 | 77.29 | C_16_H_16_O_9_S | 383.0424 | −4.70 | methyl catechin sulfate isomer 5 | 303.0763, 216.9781, 137.0255 |
| M127 | 42.29 | C_22_H_24_O_12_ | 479.1165 | −6.26 | methyl catechin glucuronide isomer 1 | 303.0878, 161.0219 |
| M128 | 51.77 | C_22_H_24_O_12_ | 479.1171 | −5.01 | methyl catechin glucuronide isomer 2 | 303.0991, 285.0713 |
| M129 | 40.51 | C_22_H_24_O_12_ | 479.1198 | 0.63 | methyl catechin glucuronide isomer 3 | 313.0573, 303.0846, 201.0408 |
| M130 | 55.14 | C_22_H_24_O_15_S | 559.0763 | 0.00 | methyl catechin glucuronide sulfate isomer 1 | 383.0448, 303.0855, 219.0671, 216.9837, 137.0293 |
| M131 | 53.57 | C_22_H_24_O_15_S | 559.0735 | −5.01 | methyl catechin glucuronide sulfate isomer 2 | 383.0473, 216.9763, 175.0452 |
| M132 | 65.48 | C_22_H_24_O_15_S | 559.0752 | −1.97 | methyl catechin glucuronide sulfate isomer 3 | 383.0486, 303.0992 |
| M133 | 81.08 | C_9_H_8_O_6_S | 242.9965 | −1.65 | *m*-coumaric acid sulfate | 163.0445, 119.0537 |
| M134 | 56.30 | C_7_H_6_O_6_S | 216.9820 | 3.69 | 3/4-hydroxy benzonic acid sulfate isomer 1 | 137.0278, 93.0130 |
| M135 | 40.62 | C_7_H_6_O_6_S | 216.9809 | −1.38 | 3/4-hydroxy benzonic acid sulfate isomer 2 | 137.0265 |
| M136 | 126.14 | C_15_H_8_O_11_S | 394.9707 | −2.03 | methyl ellagic acid sulfate isomer 1 | 315.0128, 299.9861, 172.0347 |
| M137 | 127.57 | C_15_H_8_O_11_S | 394.9710 | −1.27 | methyl ellagic acid sulfate isomer 2 | 315.0118, 299.9885 |
| M138 | 127.83 | C_13_H_8_O_7_S | 306.9927 | 2.93 | urolithin A sulfate | 227.0364 |
| M139 | 125.56 | C_13_H_8_O_6_S | 290.9960 | −3.09 | urolithin B sulfate isomer 1 | 211.0373, 167.0476 |
| M140 | 129.13 | C_13_H_8_O_6_S | 290.9959 | −3.44 | urolithin B sulfate isomer 2 | 211.0355 |
| M141 | 116.13 | C_13_H_8_O_6_S | 290.9947 | −7.65 | urolithin B sulfate isomer 3 | 211.0401 |
| M142^a^ | 126.77 | C_16_H_10_O_8_ | 329.0300 | −0.91 | 3,3'-di*-O-*methylellagic acid isomer | 314.0013, 298.9746, 285.0071, 270.9796 |
| M143 | 128.12 | C_16_H_10_O_11_S | 408.9866 | −1.22 | 3,3'-di*-O-*methylellagic acid sulfate isomer 1 | 329.0251, 314.0020, 298.9808, 270.9776 |
| M144 | 126.52 | C_16_H_10_O_11_S | 408.9867 | −0.98 | 3,3'-di*-O-*methylellagic acid sulfate isomer 2 | 329.0272, 314.0026, 298.9800, 270.9877 |
| M145 | 73.88 | C_22_H_18_O_14_ | 505.0627 | −2.18 | 3,3'-di*-O-*methylellagic acid glucuronide isomer 1 | 329.0313, 313.9887, 298.9740 |
| M146 | 74.73 | C_22_H_18_O_14_ | 505.0634 | 1.98 | 3,3'-di*-O-*methylellagic acid glucuronide isomer 2 | 329.0248, 314.0031, 298.9795 |
| M147 | 43.79 | C_8_H_8_O_8_S | 262.9867 | 0.00 | methyl gallate sulfate | 183.0258, 168.0094, 124.0129 |
| M148 | 45.96 | C_8_H_8_O_8_S | 262.9872 | 1.90 | methyl gallate sulfate isomer 1 | 183.0252, 168.0078 |
| M149 | 59.29 | C_8_H_8_O_8_S | 262.9850 | −6.46 | methyl gallate sulfate isomer 2 | 183.0230, 124.0070 |
| M150 | 61.06 | C_8_H_8_O_8_S | 262.9867 | 0.00 | methyl gallate sulfate isomer 3 | 183.0292, 124.0164 |
| M151 | 64.01 | C_8_H_8_O_8_S | 262.9852 | −5.70 | methyl gallate sulfate | 183.0283, 168.0155, 124.0023 |
| M152 | 84.88 | C_8_H_8_O_8_S | 262.9862 | −1.90 | methyl gallate sulfate isomer 4 | 183.0284, 124.0094 |
| M153 | 88.43 | C_8_H_8_O_8_S | 262.9854 | −4.94 | methyl gallate sulfate isomer 5 | 183.0306, 124.0287 |
| M154 | 125.33 | C_8_H_8_O_8_S | 262.9864 | −1.14 | methyl gallate sulfate isomer 6 | 183.0251 |
| M155 | 127.18 | C_8_H_8_O_8_S | 262.9887 | 7.60 | methyl gallate sulfate isomer 7 | 183.0405 |
| M156 | 36.25 | C_14_H_16_O_11_ | 359.0622 | 0.56 | methyl gallate glucuronide isomer 1 | 183.0316, 124.0119 |
| M157 | 37.21 | C_14_H_16_O_11_ | 359.0620 | 0.00 | methyl gallate glucuronide | 183.0297, 168.0085, 124.0199 |
| M158 | 50.09 | C_14_H_16_O_11_ | 359.0597 | −6.41 | methyl gallate glucuronide isomer 2 | 183.0204 |
| M159 | 26.75 | C_14_H_16_O_11_ | 359.0618 | −0.56 | methyl gallate glucuronide isomer 3 | 315.0613, 245.0123 |
| M160 | 40.24 | C_20_H_24_O_17_ | 535.0949 | 1.50 | methyl gallate diglucuronide isomer 1 | 359.0601, 327.0216, 209.0170, 183.0302 |
| M161 | 38.77 | C_20_H_24_O_17_ | 535.0943 | 0.37 | methyl gallate diglucuronide isomer 2 | 359.0574, 183.0241, 153.0124 |
| M162 | 58.58 | C_14_H_16_O_14_S | 439.0199 | 2.51 | methyl gallate sulfate glucuronide | 359.0642, 262.9797, 183.0185 |
| M163 | 42.90 | C_15_H_18_O_11_ | 373.0765 | −2.95 | methylated methyl gallate glucuronide isomer 1 | 183.0130, 175.0168 |
| M164 | 44.57 | C_15_H_18_O_11_ | 373.0773 | −0.80 | methylated methyl gallate glucuronide isomer 2 | 183.0198, 175.0294 |
| M165 | 43.71 | C_15_H_18_O_11_ | 373.0763 | −3.48 | methylated methyl gallate glucuronide isomer 3 | 183.0194, 175.0293 |
| M166 | 76.78 | C_9_H_10_O_8_S | 277.0014 | −3.61 | methylated methyl gallate sulfate isomer 1 | 197.0439, 182.0236 |
| M167 | 96.53 | C_9_H_10_O_8_S | 277.0015 | −3.25 | methylated methyl gallate sulfate isomer 2 | 197.0419, 182.0188 |
| M168 | 126.77 | C_9_H_10_O_8_S | 277.0018 | −2.17 | methylated methyl gallate sulfate isomer 3 | 197.0439, 169.0136 |
| M169 | 126.98 | C_10_H_12_O_8_S | 291.0165 | −5.15 | dimethylated methyl gallate sulfate | 211.0653, 196.0382 |
| M170 | 81.24 | C_7_H_8_O_4_S | 187.0064 | −3.74 | benzyl alcohol sulfate | 107.0511 |
| M171 | 50.12 | C_8_H_8_O_6_S | 230.9969 | 0.00 | 3/4-hydroxy phenylacetic acid sulfate isomer 1 | 151.0452, 107.0421 |
| M172 | 45.80 | C_8_H_8_O_6_S | 230.9952 | −5.19 | 3/4-hydroxy phenylacetic acid sulfate isomer 2 | 151.0413, 107.0421 |
| M173 | 46.27 | C_8_H_8_O_6_S | 230.9953 | −6.93 | 3/4-hydroxy phenylacetic acid sulfate isomer 3 | 151.0367, 107.0518 |
| M174 | 53.30 | C_8_H_8_O_6_S | 230.9952 | −7.36 | 3/4-hydroxy phenylacetic acid sulfate isomer 4 | 151.0395, 107.0479 |
| M175 | 48.15 | C_8_H_8_O_6_S | 230.9962 | −3.03 | 3/4-hydroxy phenylacetic acid sulfate isomer 5 | 151.0373, 107.0570 |
| M176 | 43.92 | C_8_H_8_O_6_S | 230.9962 | −3.03 | 3/4-hydroxy phenylacetic acid sulfate isomer 6 | 151.0439, 107.0486 |
| M177 | 127.45 | C_9_H_10_O_6_S | 245.0129 | 1.63 | 3/4-hydroxy phenylpropionic acid sulfate isomer 1 | 165.0441 |
| M178 | 74.60 | C_9_H_10_O_6_S | 245.0126 | 0.41 | 3/4-hydroxy phenylpropionic acid sulfate isomer 2 | 165.0569, 121.0687 |
| M179 | 70.97 | C_9_H_10_O_6_S | 245.0117 | −3.27 | 3/4-hydroxy phenylpropionic acid sulfate isomer 3 | 165.0492, 121.0618 |
| M180 | 69.45 | C_9_H_10_O_6_S | 245.0109 | −6.53 | 3/4-hydroxy phenylpropionic acid sulfate isomer 4 | 165.0492, 121.1177 |
| M181 | 55.54 | C_9_H_10_O_7_S | 261.0059 | −5.75 | 3,4-dihydroxy phenylpropionic acid sulfate isomer 1 | 217.0100, 181.0509, 137.0478 |
| M182 | 58.99 | C_9_H_10_O_7_S | 261.0083 | 3.45 | 3,4-dihydroxy phenylpropionic acid sulfate isomer 2 | 217.0219, 181.0562, 137.0661 |
| M183 | 67.81 | C_9_H_10_O_7_S | 261.0079 | 1.92 | 3,4-dihydroxy phenylpropionic acid sulfate isomer 3 | 181.0554, 137.0606 |
| M184 | 64.31 | C_9_H_10_O_7_S | 261.0088 | −5.36 | 3,4-dihydroxy phenylpropionic acid sulfate isomer 4 | 181.0434 |
| M185 | 40.17 | C_9_H_10_O_7_S | 261.0082 | 3.07 | 3,4-dihydroxy phenylpropionic acid sulfate isomer 5 | 199.0060, 181.0494 |
| M186 | 53.09 | C_9_H_10_O_7_S | 261.0062 | −4.60 | 3,4-dihydroxy phenylpropionic acid sulfate isomer 6 | 217.0316, 181.0482 |
| M187 | 44.94 | C_8_H_8_O_7_S | 246.9912 | −2.43 | 3,4-dihydroxy phenylacetic acid sulfate isomer 1 | 203.0185, 167.0327, 123.0494 |
| M188 | 49.91 | C_8_H_8_O_7_S | 246.9918 | 0.00 | 3,4-dihydroxy phenylacetic acid sulfate isomer 2 | 203.0070, 123.0389 |
| M189 | 44.24 | C_8_H_8_O_7_S | 246.9924 | 2.43 | 3,4-dihydroxy phenylacetic acid sulfate isomer 3 | 202.9952, 123.0412, 95.0412 |
| M190 | 51.43 | C_8_H_8_O_7_S | 246.9925 | 2.83 | 3,4-dihydroxy phenylacetic acid sulfate isomer 4 | 203.0154, 123.0484 |
| M191 | 41.72 | C_8_H_8_O_7_S | 246.9910 | −3.24 | 3,4-dihydroxy phenylacetic acid sulfate isomer 5 | 203.0033, 167.0398, 123.0442 |
| M192 | 125.83 | C_12_H_16_O_8_S | 319.0488 | −1.57 | dihydroxylated methoxylated benzenepentanoic acid sulfate isomer 1 | 239.0870, 221.0785, 193.0833, 177.0890 |
| M193 | 90.36 | C_12_H_16_O_8_S | 319.0507 | 4.39 | dihydroxylated methoxylated benzenepentanoic acid sulfate isomer 2 | 239.0881, 123.0459 |
| M194 | 126.80 | C_12_H_16_O_8_S | 319.0490 | −0.94 | dihydroxylated methoxylated benzenepentanoic acid sulfate isomer 3 | 239.0959, 221.0832, 193.0767, 177.0944 |
| M195 | 124.59 | C_12_H_16_O_8_S | 319.0495 | 0.63 | dihydroxylated methoxylated benzenepentanoic acid sulfate isomer 4 | 221.0806, 193.0906, 177.0913, 137.0265 |
