# Supplementary material for: Metabolism of Paeoniae Radix Rubra and its 14 constituents in mice
Source: Front Pharmacol. 2022 Oct 4;13:995641. doi: 10.3389/fphar.2022.995641 (PMC9577399; doi:10.3389/fphar.2022.995641)
Supplement: Supplementary file 2 [file Table2.DOCX]

**Supplementary material**

**Table S2.** Retention time (t_R_), molecular formula, and identification of the original constituents and metabolites of Paeonia Radix Rubra and its 14 constituents in mice by HPLC-ESI-IT-TOF-MS^n^. P, paeoniflorin; A, albiflorin; O, oxypaeoniflorin; B, benzoylpaeoniflorin; OB, hydroxybenzoylpaeoniflorin; BO, benzoyloxypaeoniflorin; G, galloylpaeoniflorin; L, lactiflorin; ECG, epicatechin gallate; CG, catechin gallate; C, catechin; EA, ellagic acid; DEA, 3,3'-di-*O*-methylellagic acid; MG, methyl gallate; PRR, Paeoniae Radix Rubra; t_R_, retention time; Meas., measured; Err., error.; U, urine; P, plasma; F, faeces; +, detected; −, undetected.

| No | t_R_(min) | Formula | Meas. (Da) | Err.  (ppm) | Identification result | U | P | F |
| --- | --- | --- | --- | --- | --- | --- | --- | --- |
| paeoniflorin | | | | | | | | |
| P0 | 56.58 | C_23_H_28_O_11_ | 525.1590 | −4.57 | paeoniflorin |  | **+** | − |
| P1 | 42.53 | C_23_H_28_O_12_ | 495.1502 | −1.21 | oxypaeoniflorin | − | **+** | − |
| P2 | 51.34 | C_16_H_24_O_10_ | 421.1333 | −4.51 | desbenzoyl paeoniflorin isomer 1 | **+** | − | − |
| P3 | 52.97 | C_16_H_24_O_10_ | 421.1346 | −1.42 | desbenzoyl paeoniflorin isomer 2 | − | **+** | − |
| P4 | 29.71 | C_17_H_26_O_10_ | 435.1485 | −5.29 | methyl debenzoyl paeoniflorin isomer 1 | − | **+** | − |
| P5 | 32.07 | C_16_H_22_O_10_ | 373.1146 | 1.61 | paeonimetabolin I glucuronide isomer 1 | **+** | − | − |
| P6 | 24.88 | C_10_H_14_O_6_S | 261.0437 | −0.38 | C_10_H_14_O_3_ sulfate isomer 1 | − | **+** | − |
| P7 | 26.01 | C_10_H_14_O_6_S | 261.0438 | 0.00 | C_10_H_14_O_3_ sulfate isomer 2 | − | **+** | − |
| P8 | 29.29 | C_10_H_14_O_6_S | 261.0423 | −5.75 | C_10_H_14_O_3_ sulfate isomer 4 | − | **+** | − |
| P9 | 34.56 | C_10_H_14_O_6_S | 261.0429 | −3.45 | C_10_H_14_O_3_ sulfate isomer 5 | − | **+** | − |
| P10 | 26.00 | C_16_H_26_O_10_ | 377.1475 | 5.83 | C_10_H_18_O_4_ glucuronide isomer 3 | **+** | − | − |
| P11 | 31.30 | C_16_H_26_O_10_ | 377.1445 | −2.12 | C_10_H_18_O_4_ glucuronide isomer 4 | **+** | − | − |
| P12 | 37.55 | C_16_H_24_O_9_ | 359.1341 | −1.95 | dehydrogenated 2,6-dihydroxycineol glucuronide isomer 1 | **+** | − | − |
| P13 | 38.25 | C_16_H_24_O_9_ | 359.1318 | −8.35 | dehydrogenated 2,6-dihydroxycineol glucuronide isomer 2 | **+** | − | − |
| P14 | 40.96 | C_16_H_24_O_9_ | 359.1319 | −8.08 | dehydrogenated 2,6-dihydroxycineol glucuronide isomer 3 | **+** | − | − |
| P15 | 47.52 | C_16_H_24_O_9_ | 359.1344 | −1.11 | dehydrogenated 2,6-dihydroxycineol glucuronide | **+** | − | − |
| P16 | 47.90 | C_10_H_18_O_6_S | 265.0732 | −7.17 | 2,6-dihydroxycineol sulfate isomer 1 | − | **+** | − |
| P17 | 51.20 | C_10_H_18_O_6_S | 265.0736 | −5.66 | 2,6-dihydroxycineol sulfate isomer 2 | − | **+** | − |
| P18 | 54.17 | C_10_H_18_O_6_S | 265.0745 | −2.26 | 2,6-dihydroxycineol sulfate isomer 3 | − | **+** | − |
| P19 | 63.27 | C_10_H_18_O_6_S | 265.0729 | −8.30 | 2,6-dihydroxycineol sulfate isomer 4 | − | **+** | − |
| P20 | 83.48 | C_10_H_18_O_6_S | 265.0739 | −4.53 | 2,6-dihydroxycineol sulfate isomer 6 | − | **+** | − |
| P21 | 85.97 | C_10_H_18_O_6_S | 265.0733 | −6.79 | 2,6-dihydroxycineol sulfate isomer 7 | − | **+** | − |
| P22 | 70.28 | C_16_H_26_O_9_ | 361.1520 | 4.43 | paeonimetabolin II glucoside isomer 3 | **+** | − | − |
| P23 | 62.24 | C_10_H_20_O_6_S | 267.0936 | 0.00 | hydrogenated 2,6-dihydroxycineol sulfate isomer 1 | − | **+** | − |
| albiflorin | | | | | | | | |
| A0 | 51.95 | C_23_H_28_O_11_ | 525.1597 | −3.24 | albiflorin | − | **+** | − |
| A1 | 8.92 | C_16_H_24_O_10_ | 421.1352 | 0.00 | desbenzoyl albiflorin isomer 1 | **+** | − | − |
| A2 | 10.08 | C_16_H_24_O_10_ | 421.1332 | −4.75 | desbenzoyl albiflorin isomer 3 | − | **+** | − |
| A3 | 31.28 | C_16_H_24_O_10_ | 375.1266 | −8.26 | desbenzoyl albiflorin isomer 3 | **+** | − | − |
| A4 | 51.42 | C_16_H_24_O_10_ | 421.1333 | −4.51 | desbenzoyl paeoniflorin isomer 1 | **+** | − | − |
| A5 | 52.92 | C_16_H_24_O_10_ | 421.1354 | 0.47 | desbenzoyl paeoniflorin isomer 2 | − | **+** | − |
| A6 | 23.69 | C_10_H_16_O_7_S | 279.0532 | −4.30 | paeonimetabolin II sulfate isomer 1 | − | **+** | − |
| A7 | 43.84 | C_10_H_16_O_7_S | 279.0524 | −7.17 | paeonimetabolin II sulfate isomer 3 | **+** | − | − |
| A8 | 49.22 | C_10_H_16_O_7_S | 279.0523 | −7.53 | paeonimetabolin II sulfate isomer 4 | − | **+** | − |
| A9 | 64.27 | C_10_H_18_O_6_S | 265.0738 | −4.90 | 2,6-dihydroxycineol sulfate isomer 5 | − | **+** | − |
| A10 | 87.16 | C_10_H_18_O_6_S | 265.0735 | −6.04 | 2,6-dihydroxycineol sulfate isomer 7 | − | **+** | − |
| A11 | 62.84 | C_10_H_20_O_6_S | 267.0885 | −8.61 | hydrogenated 2,6-dihydroxycineol sulfate isomer 1 | − | **+** | − |
| A12 | 23.65 | C_14_H_16_O_9_ | 327.0731 | 2.75 | C_8_H_8_O_3_ glucuronide isomer 1 | − | **+** | − |
| A13 | 31.11 | C_14_H_16_O_9_ | 327.0712 | −3.06 | C_8_H_8_O_3_ glucuronide isomer 2 | − | **+** | − |
| A14 | 32.32 | C_14_H_16_O_9_ | 327.0684 | 0.00 | C_8_H_8_O_3_ glucuronide isomer 3 | − | **+** | − |
| A15 | 35.98 | C_14_H_16_O_9_ | 327.0722 | 0.00 | C_8_H_8_O_3_ glucuronide isomer 4 | − | **+** | − |
| A16 | 35.70 | C_9_H_9_NO_3_ | 178.0510 | 1.12 | hippuric acid | **+** | − | − |
| oxypaeoniflorin | | | | | | | | |
| O0 | 42.99 | C_23_H_28_O_12_ | 495.1501 | −3.24 | oxypaeoniflorin | **−** | **+** | **−** |
| O1 | 65.31 | C_23_H_28_O_11_ | 525.1596 | 0.00 | paeoniflorin | **−** | **+** | **−** |
| O2 | 51.52 | C_16_H_24_O_10_ | 375.1277 | −4.75 | desbenzoyl paeoniflorin isomer 1 | **+** | **−** | **−** |
| O3 | 52.87 | C_16_H_24_O_10_ | 421.1354 | −8.26 | desbenzoyl paeoniflorin isomer 2 | **−** | **+** | **−** |
| O4 | 31.91 | C_16_H_22_O_10_ | 373.1146 | 1.61 | paeonimetabolin I glucuronide isomer 1 | **+** | **−** | **−** |
| O5 | 33.01 | C_16_H_22_O_10_ | 373.1145 | 1.34 | paeonimetabolin I glucuronide isomer 2 | **+** | **−** | **−** |
| O6 | 55.09 | C_16_H_22_O_10_ | 373.1133 | −4.30 | paeonimetabolin I glucuronide isomer 5 | **−** | **+** | **−** |
| O7 | 28.75 | C_10_H_14_O_6_S | 261.0423 | −7.17 | C_10_H_14_O_3_ sulfate isomer 3 | **−** | **+** | **−** |
| O8 | 29.81 | C_10_H_14_O_6_S | 261.0432 | −7.53 | C_10_H_14_O_3_ sulfate isomer 4 | **−** | **+** | **−** |
| O9 | 37.82 | C_10_H_14_O_6_S | 261.0432 | −4.90 | C_10_H_14_O_3_ sulfate isomer 6 | **−** | **+** | **−** |
| O10 | 23.70 | C_16_H_26_O_10_ | 377.1420 | −6.04 | C_10_H_18_O_4_ glucuronide isomer 1 | **+** | **−** | **−** |
| O11 | 25.75 | C_16_H_26_O_10_ | 377.1459 | −8.61 | C_10_H_18_O_4_ glucuronide isomer 2 | **+** | **−** | **−** |
| O12 | 31.29 | C_16_H_26_O_10_ | 377.1430 | −3.24 | C_10_H_18_O_4_ glucuronide isomer 5 | **+** | **−** | **−** |
| O13 | 37.75 | C_16_H_24_O_9_ | 359.1337 | 0.00 | dehydrogenated 2,6-dihydroxycineol glucuronide isomer 1 | **+** | **−** | **−** |
| O14 | 38.18 | C_16_H_24_O_9_ | 359.1363 | −4.75 | dehydrogenated 2,6-dihydroxycineol glucuronide isomer 2 | **+** | **−** | **−** |
| O15 | 40.77 | C_16_H_24_O_9_ | 359.1342 | −3.24 | dehydrogenated 2,6-dihydroxycineol glucuronide isomer 3 | **+** | **−** | **−** |
| O16 | 47.41 | C_16_H_24_O_9_ | 359.1309 | 0.00 | dehydrogenated 2,6-dihydroxycineol glucuronide | **+** | **−** | **−** |
| O17 | 34.25 | C_10_H_18_O_6_S | 265.0730 | −4.75 | 2,6-dihydroxycineol sulfate isomer 1 | **−** | **+** | **−** |
| O18 | 47.15 | C_10_H_18_O_6_S | 265.0747 | −8.26 | 2,6-dihydroxycineol sulfate isomer 2 | **−** | **+** | **−** |
| O19 | 51.50 | C_10_H_18_O_6_S | 265.0736 | −5.66 | 2,6-dihydroxycineol sulfate isomer 3 | **−** | **+** | **−** |
| O20 | 54.32 | C_10_H_18_O_6_S | 265.0730 | 0.47 | 2,6-dihydroxycineol sulfate isomer 4 | **−** | **+** | **−** |
| O21 | 56.90 | C_10_H_18_O_6_S | 265.0722 | −4.30 | 2,6-dihydroxycineol sulfate isomer 5 | **−** | **+** | **−** |
| O22 | 66.21 | C_10_H_18_O_6_S | 265.0729 | −7.17 | 2,6-dihydroxycineol sulfate isomer 6 | **−** | **+** | **−** |
| O23 | 88.81 | C_10_H_18_O_6_S | 265.0750 | −7.53 | 2,6-dihydroxycineol sulfate isomer 7 | **−** | **+** | **−** |
| O24 | 64.35 | C_10_H_20_O_6_S | 267.0909 | −4.90 | hydrogenated 2,6-dihydroxycineol sulfate isomer 1 | **−** | **+** | **−** |
| benzoylpaeoniflorin | | | | | | | | |
| B1 | 56.21 | C_23_H_28_O_11_ | 525.1582 | −6.09 | paeoniflorin | − | **+** | − |
| B2 | 51.43 | C_16_H_24_O_10_ | 375.1265 | −8.53 | desbenzoyl paeoniflorin isomer 1 | **+** | − | − |
| B3 | 52.77 | C_16_H_24_O_10_ | 421.1323 | −6.89 | desbenzoyl paeoniflorin isomer 2 | − | **+** | − |
| B4 | 32.00 | C_16_H_22_O_10_ | 373.1146 | 1.61 | paeonimetabolin I glucuronide isomer 1 | **+** | − | − |
| B5 | 33.42 | C_16_H_22_O_10_ | 373.1163 | 6.16 | paeonimetabolin I glucuronide isomer 2 | **+** | − | − |
| B6 | 24.34 | C_10_H_14_O_6_S | 261.0438 | 0.00 | C_10_H_14_O_3_ sulfate isomer 1 | − | **+** | − |
| B7 | 25.57 | C_10_H_14_O_6_S | 261.0437 | −0.38 | C_10_H_14_O_3_ sulfate isomer 2 | − | **+** | − |
| B8 | 33.92 | C_10_H_14_O_6_S | 261.0429 | −3.45 | C_10_H_14_O_3_ sulfate isomer 3 | − | **+** | − |
| B9 | 26.08 | C_16_H_26_O_10_ | 377.1461 | 2.12 | C_10_H_18_O_4_ glucuronide isomer 3 | **+** | − | − |
| B10 | 31.30 | C_16_H_26_O_10_ | 377.1457 | 1.06 | C_10_H_18_O_4_ glucuronide isomer 5 | **+** | − | − |
| B11 | 37.70 | C_16_H_24_O_9_ | 359.1364 | 4.46 | dehydrogenated 2,6-dihydroxycineol glucuronide isomer 1 | **+** | − | − |
| B12 | 38.22 | C_16_H_24_O_9_ | 359.1381 | 9.19 | dehydrogenated 2,6-dihydroxycineol glucuronide isomer 2 | **+** | − | − |
| B13 | 47.48 | C_16_H_24_O_9_ | 359.1322 | −7.24 | dehydrogenated 2,6-dihydroxycineol glucuronide | **+** | − | − |
| B14 | 50.38 | C_10_H_18_O_6_S | 265.0736 | −5.66 | 2,6-dihydroxycineol sulfate isomer 3 | − | **+** | − |
| B15 | 53.46 | C_10_H_18_O_6_S | 265.0733 | −6.79 | 2,6-dihydroxycineol sulfate isomer 4 | − | **+** | − |
| B16 | 62.91 | C_10_H_18_O_6_S | 265.0750 | −0.38 | 2,6-dihydroxycineol sulfate isomer 5 | − | **+** | − |
| B17 | 84.99 | C_10_H_18_O_6_S | 265.0760 | 3.40 | 2,6-dihydroxycineol sulfate isomer 7 | − | **+** | − |
| hydroxybenzoylpaeoniflorin | | | | | | | | |
| OB0 | 84.65 | C_30_H_32_O_13_ | 599.1726 | −7.34 | hydroxybenzoylpaeoniflorin | − | **+** | − |
| OB1 | 57.28 | C_23_H_28_O_12_ | 495.1467 | −8.28 | oxypaeoniflorin | − | **+** | − |
| OB2 | 56.27 | C_23_H_28_O_11_ | 525.1588 | −4.95 | paeoniflorin | − | **+** | − |
| OB3 | 51.56 | C_16_H_24_O_10_ | 375.1322 | 6.66 | desbenzoyl paeoniflorin isomer 1 | **+** | − | − |
| OB4 | 52.87 | C_16_H_24_O_10_ | 421.1351 | −0.24 | desbenzoyl paeoniflorin isomer 2 | − | **+** | − |
| OB5 | 31.85 | C_16_H_22_O_10_ | 373.1146 | 1.61 | paeonimetabolin I glucuronide isomer 1 | **+** | − | − |
| OB6 | 33.33 | C_16_H_22_O_10_ | 373.1124 | −4.29 | paeonimetabolin I glucuronide isomer 2 | **+** | − | − |
| OB7 | 24.04 | C_10_H_14_O_6_S | 261.0421 | −6.51 | C_10_H_14_O_3_ sulfate isomer 1 | − | **+** | − |
| OB8 | 25.14 | C_10_H_14_O_6_S | 261.0415 | −8.81 | C_10_H_14_O_3_ sulfate isomer 2 | − | **+** | − |
| OB9 | 28.42 | C_10_H_14_O_6_S | 261.0417 | −8.04 | C_10_H_14_O_3_ sulfate isomer 4 | − | **+** | − |
| OB10 | 32.78 | C_10_H_14_O_6_S | 261.0421 | −6.51 | C_10_H_14_O_3_ sulfate isomer 5 | − | **+** | − |
| OB11 | 24.06 | C_16_H_26_O_10_ | 377.1433 | −5.30 | C_10_H_18_O_4_ glucuronide isomer 1 | **+** | − | − |
| OB12 | 26.08 | C_16_H_26_O_10_ | 377.1451 | −0.53 | C_10_H_18_O_4_ glucuronide isomer 3 | **+** | − | − |
| OB13 | 47.45 | C_16_H_24_O_9_ | 359.1373 | 6.96 | dehydrogenated 2,6-dihydroxycineol glucuronide | **+** | − | − |
| OB14 | 31.89 | C_10_H_16_O_7_S | 279.0525 | −6.81 | paeonimetabolin II sulfate isomer 2 | − | **+** | − |
| OB15 | 46.35 | C_10_H_18_O_6_S | 265.0739 | −4.53 | 2,6-dihydroxycineol sulfate isomer 2 | − | **+** | − |
| OB16 | 49.50 | C_10_H_18_O_6_S | 265.0739 | −4.53 | 2,6-dihydroxycineol sulfate isomer 3 | − | **+** | − |
| OB17 | 62.10 | C_10_H_18_O_6_S | 265.0734 | −6.41 | 2,6-dihydroxycineol sulfate isomer 5 | − | **+** | − |
| OB18 | 60.40 | C_10_H_20_O_6_S | 267.0882 | −9.73 | hydrogenated 2,6-dihydroxycineol sulfate isomer 1 | − | **+** | − |
| benzoyloxypaeoniflorin | | | | | | | | |
| BO0 | 87.12 | C_30_H_32_O_13_ | 599.1755 | −2.50 | benzoyloxypaeoniflorin | − | **+** | − |
| BO1 | 42.57 | C_23_H_28_O_12_ | 495.1491 | −3.43 | oxypaeoniflorin | − | **+** | − |
| BO2 | 52.89 | C_16_H_24_O_10_ | 375.1300 | 0.80 | desbenzoyl paeoniflorin isomer 2 | − | **+** | − |
| BO3 | 32.69 | C_16_H_22_O_10_ | 373.1137 | −0.80 | paeonimetabolin I glucuronide isomer 1 | **+** | − | − |
| BO4 | 34.07 | C_16_H_22_O_10_ | 373.1142 | 0.54 | paeonimetabolin I glucuronide isomer 2 | **+** | − | − |
| BO5 | 77.90 | C_10_H_16_O_4_ | 199.0987 | 5.52 | paeonimetabolin II isomer 1 | **+** | − | − |
| BO6 | 80.24 | C_10_H_16_O_4_ | 199.0970 | −3.01 | paeonimetabolin II isomer 4 | **+** | − | − |
| BO7 | 24.32 | C_10_H_14_O_6_S | 261.0449 | 4.21 | C_10_H_14_O_3_ sulfate isomer 1 | − | **+** | − |
| BO8 | 25.15 | C_10_H_14_O_6_S | 261.0441 | 1.15 | C_10_H_14_O_3_ sulfate isomer 2 | − | **+** | − |
| BO9 | 24.33 | C_16_H_26_O_10_ | 377.1475 | 5.83 | C_10_H_18_O_4_ glucuronide isomer 1 | **+** | − | − |
| BO10 | 24.90 | C_16_H_26_O_10_ | 377.1443 | −2.65 | C_10_H_18_O_4_ glucuronide isomer 2 | **+** | − | − |
| BO11 | 26.71 | C_16_H_26_O_10_ | 377.1463 | 2.65 | C_10_H_18_O_4_ glucuronide isomer 3 | **+** | − | − |
| BO12 | 30.81 | C_16_H_26_O_10_ | 377.1453 | 0.00 | C_10_H_18_O_4_ glucuronide isomer 4 | **+** | − | − |
| BO13 | 31.97 | C_16_H_26_O_10_ | 377.1437 | −4.24 | C_10_H_18_O_4_ glucuronide isomer 5 | **+** | − | − |
| BO14 | 56.96 | C_16_H_28_O_10_ | 379.1585 | −6.59 | C_10_H_20_O_4_ glucuronide | **+** | − | − |
| BO15 | 38.30 | C_16_H_24_O_9_ | 359.1318 | −8.35 | dehydrogenated 2,6-dihydroxycineol glucuronide isomer 1 | **+** | − | − |
| BO16 | 39.01 | C_16_H_24_O_9_ | 359.1343 | −1.39 | dehydrogenated 2,6-dihydroxycineol glucuronide isomer 2 | **+** | − | − |
| BO17 | 39.61 | C_16_H_24_O_9_ | 359.1342 | −1.67 | dehydrogenated 2,6-dihydroxycineol glucuronide isomer 3 | **+** | − | − |
| BO18 | 41.87 | C_16_H_24_O_9_ | 359.1322 | −7.24 | dehydrogenated 2,6-dihydroxycineol glucuronide isomer 4 | **+** | − | − |
| BO19 | 48.65 | C_16_H_24_O_9_ | 359.1333 | −4.18 | dehydrogenated 2,6-dihydroxycineol glucuronide | **+** | − | − |
| BO20 | 49.63 | C_16_H_24_O_9_ | 359.1353 | 1.39 | dehydrogenated 2,6-dihydroxycineol glucuronide isomer 5 | **+** | − | − |
| BO21 | 61.97 | C_10_H_18_O_6_S | 265.0727 | −9.05 | 2,6-dihydroxycineol sulfate isomer 5 | − | **+** | − |
| BO22 | 84.40 | C_10_H_18_O_6_S | 265.0727 | −9.05 | 2,6-dihydroxycineol sulfate isomer 7 | − | **+** | − |
| BO23 | 73.23 | C_16_H_26_O_9_ | 361.1497 | −1.94 | paeonimetabolin II glucoside isomer 4 | **+** | − | − |
| BO24 | 61.02 | C_10_H_20_O_6_S | 267.0921 | 4.87 | hydrogenated 2,6-dihydroxycineol sulfate isomer 1 | − | **+** | − |
| BO25 | 30.75 | C_14_H_16_O_9_ | 327.0692 | −9.17 | C_8_H_8_O_3_ glucuronide isomer 2 | **+** | − | − |
| BO26 | 41.65 | C_14_H_16_O_9_ | 327.0696 | −7.95 | C_8_H_8_O_3_ glucuronide isomer 5 | **+** | − | − |
| BO27 | 28.31 | C_13_H_14_O_9_ | 313.0547 | −5.75 | salicylic acid glucuronide | **+** | − | − |
| BO28 | 27.64 | C_9_H_9_NO_4_ | 194.0469 | 5.15 | hydroxyhippuric acid | **+** | − | − |
| BO29 | 29.07 | C_9_H_9_NO_4_ | 194.0459 | 0.00 | hydroxyhippuric acid | **+** | − | − |
| BO30 | 36.71 | C_9_H_9_NO_3_ | 178.0510 | 0.00 | hippuric acid | **+** | − | − |
| galloylpaeoniflorin | | | | | | | | |
| G0 | 70.17 | C_30_H_32_O_15_ | 631.1633 | −5.55 | galloylpaeoniflorin |  |  |  |
| G1 | 56.43 | C_23_H_28_O_11_ | 525.1598 | −3.05 | paeoniflorin | − | **+** | − |
| G2 | 52.78 | C_16_H_24_O_10_ | 375.1290 | −1.87 | desbenzoyl paeoniflorin isomer 2 | − | **+** | − |
| G3 | 32.73 | C_16_H_22_O_10_ | 373.1155 | 4.02 | paeonimetabolin I glucuronide isomer 1 | − | **+** | − |
| G4 | 33.71 | C_16_H_22_O_10_ | 373.1142 | 0.54 | paeonimetabolin I glucuronide isomer 2 | **+** | − | − |
| G5 | 77.85 | C_10_H_16_O_4_ | 199.0971 | −2.51 | paeonimetabolin II isomer 1 | **+** | − | − |
| G6 | 80.14 | C_10_H_16_O_4_ | 199.0958 | −9.04 | paeonimetabolin II isomer 4 | **+** | − | − |
| G7 | 24.14 | C_10_H_14_O_6_S | 261.0451 | 4.98 | C_10_H_14_O_3_ sulfate isomer 1 | **+** | − | − |
| G8 | 24.81 | C_10_H_14_O_6_S | 261.0430 | −3.06 | C_10_H_14_O_3_ sulfate isomer 2 | − | **+** | − |
| G9 | 45.96 | C_10_H_16_O_7_S | 279.0546 | 0.72 | paeonimetabolin II sulfate isomer 4 | − | **+** | − |
| G10 | 24.69 | C_16_H_26_O_10_ | 377.1445 | −2.12 | C_10_H_18_O_4_ glucuronide isomer 2 | **+** | − | − |
| G11 | 26.66 | C_16_H_26_O_10_ | 377.1435 | −4.77 | C_10_H_18_O_4_ glucuronide isomer 3 | − | **+** | − |
| G12 | 30.68 | C_16_H_26_O_10_ | 377.1452 | −0.27 | C_10_H_18_O_4_ glucuronide isomer 4 | **+** | − | − |
| G13 | 32.25 | C_16_H_26_O_10_ | 377.1435 | −4.77 | C_10_H_18_O_4_ glucuronide isomer 5 | **+** | − | − |
| G14 | 56.90 | C_16_H_28_O_10_ | 379.1614 | 1.05 | C_10_H_20_O_4_ glucuronide | **+** | − | − |
| G15 | 38.39 | C_16_H_24_O_9_ | 359.1320 | −7.80 | dehydrogenated 2,6-dihydroxycineol glucuronide isomer 1 | **+** | − | − |
| G16 | 39.03 | C_16_H_24_O_9_ | 359.1321 | −7.52 | dehydrogenated 2,6-dihydroxycineol glucuronide isomer 2 | **+** | − | − |
| G17 | 42.18 | C_16_H_24_O_9_ | 359.1347 | −0.28 | dehydrogenated 2,6-dihydroxycineol glucuronide isomer 4 | **+** | − | − |
| G18 | 48.42 | C_16_H_24_O_9_ | 359.1330 | −5.01 | dehydrogenated 2,6-dihydroxycineol glucuronide | **+** | − | − |
| G19 | 61.86 | C_10_H_18_O_6_S | 265.0739 | −4.53 | 2,6-dihydroxycineol sulfate isomer 5 | **+** | − | − |
| G20 | 83.70 | C_10_H_18_O_6_S | 265.0757 | 2.26 | 2,6-dihydroxycineol sulfate isomer 6 | − | **+** | − |
| G21 | 73.25 | C_16_H_26_O_9_ | 361.1496 | −2.22 | paeonimetabolin II glucoside isomer 4 | − | **+** | − |
| G22 | 60.55 | C_10_H_20_O_6_S | 267.0890 | −6.74 | hydrogenated 2,6-dihydroxycineol sulfate isomer 1 | **+** | − | − |
| G23 | 30.74 | C_14_H_16_O_9_ | 327.0711 | −3.36 | C_8_H_8_O_3_ glucuronide isomer 2 | − | **+** | − |
| G24 | 41.70 | C_14_H_16_O_9_ | 327.0696 | −7.95 | C_8_H_8_O_3_ glucuronide isomer 5 | **+** | − | − |
| G25 | 33.80 | C_7_H_6_O_8_S | 248.9691 | 5.62 | gallic acid sulfate | **+** | − | − |
| G26 | 42.07 | C_7_H_6_O_8_S | 248.9719 | 3.21 | gallic acid sulfate | **+** | − | − |
| G27 | 37.17 | C_9_H_9_NO_3_ | 178.0516 | 3.37 | hippuric acid | **+** | − | − |
| lactiflorin | | | | | | | | |
| L0 | 78.93 | C_23_H_26_O_10_ | 507.1493 | −2.96 | lactiflorin | − | − | **+** |
| L1 | 71.03 | C_23_H_28_O_10_ | 509.1654 | −2.16 | hydrogenated lactiflorin | − | − | **+** |
| L2 | 63.56 | C_23_H_28_O_10_ | 509.1652 | −2.55 | hydrogenated lactiflorin isomer | − | − | **+** |
| L3 | 71.64 | C_23_H_28_O_11_ | 525.1598 | −3.05 | hydrogenated hydroxylated lactiflorin | − | − | **+** |
| L4 | 53.48 | C_16_H_24_O_10_ | 421.1350 | −0.47 | desbenzoyl paeoniflorin isomer 2 | **+** | − | − |
| L5 | 29.78 | C_16_H_22_O_10_ | 373.1123 | −4.56 | paeonimetabolin I glucuronide isomer 3 | **+** | − | − |
| L6 | 28.88 | C_16_H_22_O_10_ | 373.1135 | −1.34 | paeonimetabolin I glucuronide isomer 4 | **+** | − | − |
| L7 | 53.55 | C_16_H_26_O_10_ | 377.1451 | −0.53 | C_10_H_18_O_4_ glucuronide isomer 6 | **+** | − | − |
| L8 | 85.18 | C_17_H_18_O_8_S | 381.0612 | −1.05 | hydrogenated deglycosylated lactiflorin sulfate isomer 1 | **+** | − | − |
| L9 | 66.53 | C_17_H_18_O_8_S | 381.0625 | 2.36 | hydrogenated deglycosylated lactiflorin sulfate isomer 2 | − | − | **+** |
| L10 | 84.31 | C_17_H_18_O_8_S | 381.0622 | 1.57 | hydrogenated deglycosylated lactiflorin sulfate isomer 3 | − | − | **+** |
| L11 | 100.37 | C_17_H_18_O_8_S | 381.0629 | 3.41 | hydrogenated deglycosylated lactiflorin sulfate isomer 4 | − | − | **+** |
| L12 | 125.83 | C_12_H_16_O_8_S | 319.0490 | −0.94 | dihydroxylated methoxylated benzenepentanoic acid sulfate isomer 1 | **+** | − | − |
| L13 | 50.12 | C_8_H_8_O_6_S | 230.9969 | 0.00 | 3/4-hydroxy phenylacetic acid sulfate isomer 1 | **+** | − | − |
| L14 | 45.80 | C_8_H_8_O_6_S | 230.9952 | −5.19 | 3/4-hydroxy phenylacetic acid sulfate isomer 2 | **+** | − | − |
| L15 | 127.45 | C_9_H_10_O_6_S | 245.0129 | 1.63 | 3/4-hydroxy phenylpropionic acid sulfate isomer 1 | **+** | − | − |
| L16 | 44.94 | C_8_H_8_O_7_S | 246.9912 | −2.43 | 3,4-dihydroxy phenylacetic acid sulfate isomer 1 | **+** | − | − |
| L17 | 55.54 | C_9_H_10_O_7_S | 261.0059 | −5.75 | 3,4-dihydroxy phenylpropionic acid sulfate isomer 1 | **+** | − | − |
| epicatechin gallate | | | | | | | | |
| ECG0 | 67.80 | C_22_H_18_O_10_ | 441.0827 | 0.00 | epicatechin gallate | − | − | **+** |
| ECG1 | 40.29 | C_12_H_14_O_12_S | 381.0166 | 8.66 | pyrogallol-*O*-glucuronide sulfate isomer 1 | **+** | − | − |
| ECG2 | 71.09 | C_15_H_14_O_9_S | 369.0295 | 2.44 | epicatechin sulfate | − | − | **+** |
| ECG3 | 83.80 | C_15_H_16_O_8_S | 355.0485 | −2.25 | 3-HPP-2-ol sulfate isomer 1 | − | − | **+** |
| ECG4 | 85.03 | C_15_H_16_O_8_S | 355.0485 | −2.25 | 3-HPP-2-ol sulfate isomer 2 | − | − | **+** |
| ECG5 | 85.09 | C_16_H_16_O_9_S | 383.0449 | 1.83 | methyl catechin sulfate isomer 1 | − | − | **+** |
| ECG6 | 50.13 | C_11_H_14_O_4_ | 209.0812 | −3.35 | 5-(3,4-dihydroxyphenyl)-valeric acid | − | − | **+** |
| ECG7 | 76.97 | **C_10_H_10_O_7_S** | 273.0072 | −0.73 | **ferulic acid sulfate** | **+** | − | − |
| ECG8 | 69.82 | C_11_H_14_O_7_S | 289.0386 | −0.35 | 5-(3,4-dihydroxyphenyl)-valeric acid sulfate isomer 1 | **+** | − | − |
| ECG9 | 64.15 | C_11_H_14_O_8_S | 305.0348 | 3.61 | trihydroxy benzenepentanoic acid sulfate isomer 1 | **+** | − | − |
| ECG10 | 90.36 | C_12_H_16_O_8_S | 319.0507 | 4.39 | dihydroxylated methoxylated benzenepentanoic acid sulfate isomer 2 | **+** | − | − |
| ECG11 | 87.39 | C_11_H_12_O_6_S | 271.0286 | 1.48 | 5-(3-hydroxyphenyl)-γ-valerolactone sulfate isomer 1 | **+** | − | − |
| ECG12 | 79.45 | C_11_H_12_O_7_S | 287.0251 | 2.44 | 5-(3,4-dihydroxyphenyl)-γ-valerolactone sulfate isomer 1 | **+** | − | − |
| ECG13 | 72.89 | C_11_H_12_O_7_S | 287.0243 | 4.18 | 5-(3,4-dihydroxyphenyl)-γ-valerolactone sulfate isomer 2 | **+** | − | − |
| ECG14 | 46.82 | C_17_H_20_O_10_ | 383.1002 | 4.70 | 5-(3,4-dihydroxyphenyl)-γ-valerolactone glucuronide isomer 1 | **+** | − | − |
| ECG15 | 58.99 | C_9_H_10_O_7_S | 261.0083 | 3.45 | 3,4-dihydroxy phenylpropionic acid sulfate isomer 2 | **+** | − | − |
| ECG16 | 67.81 | C_9_H_10_O_7_S | 261.0079 | 1.92 | 3,4-dihydroxy phenylpropionic acid sulfate isomer 3 | **+** | − | − |
| ECG17 | 74.60 | C_9_H_10_O_6_S | 245.0126 | 0.41 | 3/4-hydroxy phenylpropionic acid sulfate isomer 2 | **+** | − | − |
| ECG18 | 52.55 | C_8_H_8_O_6_S | 230.9976 | 3.03 | 3/4-hydroxy phenylacetic acid sulfate isomer 4 | **+** | − | − |
| ECG19 | 56.30 | C_7_H_6_O_6_S | 216.9820 | 3.69 | 3/4-hydroxy benzonic acid sulfate isomer 1 | **+** | − | − |
| ECG20 | 49.91 | C_8_H_8_O_7_S | 246.9918 | 0.00 | 3,4-dihydroxy phenylacetic acid sulfate isomer 2 | **+** | − | − |
| ECG21 | 51.43 | C_8_H_8_O_7_S | 246.9925 | 2.83 | 3,4-dihydroxy phenylacetic acid sulfate isomer 4 | **+** | − | − |
| ECG22 | 81.08 | C_9_H_8_O_6_S | 242.9965 | −1.65 | *m*-coumaric acid sulfate | **+** | − | − |
| catechin gallate | | | | | | | | |
| CG0 | 70.36 | C_22_H_18_O_10_ | 441.0828 | 0.23 | catechin gallate | − | − | **+** |
| CG1 | 78.53 | C_23_H_20_O_10_ | 455.0985 | 0.22 | methyl catechin gallate | − | − | **+** |
| CG2 | 40.07 | C_15_H_14_O_6_ | 289.0706 | −4.15 | catechin | − | − | **+** |
| CG3 | 95.25 | C_21_H_24_O_15_S | 547.1477 | 3.66 | 3,4-diHPP-2-ol glucuronide sulfate | **+** | − | − |
| CG4 | 66.43 | C_21_H_22_O_15_S | 545.0599 | −1.47 | catechin glucuronide sulfate isomer 1 | **+** | − | − |
| CG5 | 66.12 | C_15_H_14_O_9_S | 369.0285 | −0.27 | catechin 5/7-*O*-sulfate isomer 1 | − | − | **+** |
| CG6 | 67.48 | C_15_H_14_O_9_S | 369.0268 | −4.88 | catechin sulfate isomer 2 | − | − | **+** |
| CG7 | 72.48 | C_15_H_14_O_9_S | 369.0280 | −1.63 | catechin sulfate isomer 3 | − | − | **+** |
| CG8 | 82.21 | C_16_H_16_O_9_S | 383.0456 | 3.65 | methyl catechin sulfate isomer 2 | − | − | **+** |
| CG9 | 53.30 | C_8_H_8_O_6_S | 230.9952 | −7.36 | 3/4-hydroxy phenylacetic acid sulfate isomer 4 | **+** | − | − |
| CG10 | 75.08 | C_9_H_10_O_6_S | 245.0110 | −6.12 | 3/4-hydroxy phenylpropionic acid sulfate isomer 2 | **+** | − | − |
| CG11 | 50.11 | C_8_H_8_O_7_S | 246.9915 | −1.21 | 3,4-dihydroxy phenylacetic acid sulfate isomer 2 | **+** | − | − |
| CG12 | 77.30 | C_10_H_10_O_7_S | 273.0068 | −2.20 | ferulic acid sulfate | **+** | − | − |
| CG13 | 82.65 | C_11_H_12_O_7_S | 287.0236 | 1.74 | 5-(3,4-dihydroxyphenyl)-γ-valerolactone sulfate isomer 1 | **+** | − | − |
| CG14 | 80.43 | C_11_H_12_O_7_S | 287.0225 | −2.09 | 5-(3,4-dihydroxyphenyl)-γ-valerolactone sulfate isomer 3 | **+** | − | − |
| CG15 | 47.38 | C_17_H_20_O_10_ | 383.1005 | 5.48 | 5-(3,4-dihydroxyphenyl)-γ-valerolactone glucuronide isomer 2 | **+** | − | − |
| CG16 | 40.82 | C_12_H_14_O_12_S | 381.0138 | 1.31 | pyrogallol-*O*-glucuronide sulfate isomer 1 | **+** | − | − |
| CG17 | 41.84 | C_12_H_14_O_12_S | 381.0149 | 4.20 | pyrogallol-*O*-glucuronide sulfate isomer 2 | **+** | − | − |
| catechin | | | | | | | | |
| C1 | 37.64 | C_21_H_22_O_12_ | 465.1038 | 0.00 | catechin glucuronide isomer 1 | **+** | − | − |
| C2 | 36.14 | C_21_H_22_O_12_ | 465.1008 | −6.45 | catechin glucuronide isomer 2 | **+** | − | − |
| C3 | 32.94 | C_21_H_22_O_12_ | 465.1053 | 3.23 | catechin glucuronide isomer 3 | **+** | − | − |
| C4 | 61.79 | C_15_H_14_O_9_S | 369.0283 | −0.81 | catechin 5/7-*O*-sulfate isomer 2 | **+** | − | − |
| C5 | 68.11 | C_15_H_14_O_9_S | 369.0283 | −0.81 | catechin 3'/4'-*O*-sulfate isomer | **+** | − | − |
| C6 | 61.43 | C_21_H_22_O_15_S | 545.0576 | −5.69 | catechin glucuronide sulfate isomer 2 | **+** | − | − |
| C7 | 54.85 | C_21_H_22_O_15_S | 545.0621 | 2.57 | catechin glucuronide sulfate isomer 3 | **+** | − | − |
| C8 | 42.29 | C_22_H_24_O_12_ | 479.1165 | −6.26 | methyl catechin glucuronide isomer 1 | **+** | − | − |
| C9 | 51.77 | C_22_H_24_O_12_ | 479.1171 | −5.01 | methyl catechin glucuronide isomer 2 | **+** | − | − |
| C10 | 40.51 | C_22_H_24_O_12_ | 479.1198 | 0.63 | methyl catechin glucuronide isomer 3 | **+** | − | − |
| C11 | 81.83 | C_16_H_16_O_9_S | 383.0454 | 3.13 | methyl catechin sulfate isomer 3 | **+** | − | − |
| C12 | 75.73 | C_16_H_16_O_9_S | 383.0458 | 4.18 | methyl catechin sulfate isomer 4 | **+** | − | − |
| C13 | 77.29 | C_16_H_16_O_9_S | 383.0424 | −4.70 | methyl catechin sulfate isomer 5 | **+** | − | **+** |
| C14 | 55.14 | C_22_H_24_O_15_S | 559.0763 | 0.00 | methyl catechin glucuronide sulfate isomer 1 | − | **+** | − |
| C15 | 53.57 | C_22_H_24_O_15_S | 559.0735 | −5.01 | methyl catechin glucuronide sulfate isomer 2 | − | **+** | − |
| C16 | 65.48 | C_22_H_24_O_15_S | 559.0752 | −1.97 | methyl catechin glucuronide sulfate isomer 3 | − | **+** | − |
| C17 | 99.94 | C_15_H_16_O_6_ | 291.0891 | 5.84 | **3,4-diHPP-2-ol** | **+** | − | − |
| C18 | 79.52 | C_15_H_16_O_8_S | 355.0473 | −5.63 | 3-HPP-2-ol sulfate isomer 3 | − | − | **+** |
| C19 | 67.91 | C_21_H_24_O_14_S | 531.0796 | −3.39 | **3-HPP-2-ol glucuronide sulfate** | **+** | − | − |
| C20 | 125.97 | C_11_H_14_O_7_S | 289.0400 | 4.50 | 5-(3,4-dihydroxyphenyl)-valeric acid sulfate isomer 2 | **+** | − | − |
| C21 | 66.80 | C_11_H_14_O_7_S | 289.0368 | −6.57 | 5-(3,4-dihydroxyphenyl)-valeric acid sulfate isomer 3 | **+** | − | − |
| C22 | 77.34 | C_11_H_12_O_7_S | 287.0227 | −1.39 | 5-(3,4-dihydroxyphenyl)-γ-valerolactone sulfate isomer 3 | **+** | − | − |
| C23 | 76.08 | C_11_H_12_O_7_S | 287.0219 | −4.18 | 5-(3,4-dihydroxyphenyl)-γ-valerolactone sulfate isomer 4 | **+** | − | − |
| C24 | 82.81 | C_11_H_12_O_6_S | 271.0284 | 0.74 | 5-(3-hydroxyphenyl)-γ-valerolactone sulfate isomer 2 | **+** | − | − |
| C25 | 60.56 | C_11_H_14_O_8_S | 305.0316 | −6.88 | trihydroxy benzenepentanoic acid sulfate isomer 2 | **+** | − | − |
| C26 | 125.52 | C_12_H_16_O_8_S | 319.0493 | 0.00 | dihydroxylated methoxylated benzenepentanoic acid sulfate isomer 1 | − | **+** | − |
| C27 | 126.80 | C_12_H_16_O_8_S | 319.0490 | −0.94 | dihydroxylated methoxylated benzenepentanoic acid sulfate isomer 3 | **+** | − | − |
| C28 | 44.07 | C_8_H_8_O_7_S | 246.9901 | −6.88 | 3,4-dihydroxy phenylacetic acid sulfate isomer 3 | **+** | − | − |
| C29 | 50.04 | C_8_H_8_O_6_S | 230.9958 | −4.76 | 3/4-hydroxy phenylacetic acid sulfate isomer 1 | **+** | − | − |
| C30 | 46.27 | C_8_H_8_O_6_S | 230.9953 | −6.93 | 3/4-hydroxy phenylacetic acid sulfate isomer 3 | **+** | − | − |
| C31 | 70.97 | C_9_H_10_O_6_S | 245.0117 | −3.27 | 3/4-hydroxy phenylpropionic acid sulfate isomer 3 | **+** | − | − |
| C32 | 69.45 | C_9_H_10_O_6_S | 245.0109 | −6.53 | 3/4-hydroxy phenylpropionic acid sulfate isomer 4 | **+** | − | − |
| C33 | 64.31 | C_9_H_10_O_7_S | 261.0088 | −5.36 | 3,4-dihydroxy phenylpropionic acid sulfate isomer 4 | **+** | − | − |
| ellagic acid | | | | | | | | |
| EA1 | 126.14 | C_15_H_8_O_11_S | 394.9707 | −2.03 | methyl ellagic acid sulfate isomer 1 | **+** | − | − |
| EA2 | 127.57 | C_15_H_8_O_11_S | 394.9710 | −1.27 | methyl ellagic acid sulfate isomer 2 | **+** | − | − |
| EA3 | 125.56 | C_13_H_8_O_6_S | 290.9960 | −3.09 | urolithin B sulfate isomer 1 | − | − | **+** |
| 3,3'-di-*O*-methyl ellagic acid | | | | | | | | |
| DEA0 | 90.97 | C_16_H_10_O_8_ | 329.0315 | 3.65 | 3,3'-di-*O*-methyl ellagic acid | **+** | − | − |
| DEA1 | 126.77 | C_16_H_10_O_8_ | 329.0300 | −0.91 | 3,3'-di-*O*-methyl ellagic acid isomer | **+** | − | − |
| DEA2 | 128.12 | C_16_H_10_O_11_S | 408.9866 | −1.22 | 3,3'-di-*O*-methyl ellagic acid sulfate isomer 1 | **+** | − | − |
| DEA3 | 126.52 | C_16_H_10_O_11_S | 408.9867 | −0.98 | 3,3'-di-*O*-methyl ellagic acid sulfate isomer 2 | − | − | **+** |
| DEA4 | 73.88 | C_22_H_18_O_14_ | 505.0627 | −2.18 | 3,3'-di-*O*-methyl ellagic acid glucuronide isomer 1 | **+** | − | − |
| DEA5 | 74.73 | C_22_H_18_O_14_ | 505.0634 | 1.98 | 3,3'-di-*O*-methyl ellagic acid glucuronide isomer 2 | **+** | − | − |
| DEA6 | 127.83 | C_13_H_8_O_7_S | 306.9927 | 2.93 | urolithin A sulfate | **+** | − | − |
| DEA7 | 125.68 | C_13_H_8_O_6_S | 290.9975 | 2.06 | urolithin B sulfate isomer 1 | − | − | **+** |
| DEA8 | 129.13 | C_13_H_8_O_6_S | 290.9959 | −3.44 | urolithin B sulfate isomer 2 | **+** | − | − |
| methyl gallate | | | | | | | | |
| MG1 | 43.79 | C_8_H_8_O_8_S | 262.9867 | 0.00 | methyl gallate sulfate | **+** | − | − |
| MG 2 | 45.96 | C_8_H_8_O_8_S | 262.9872 | 1.90 | methyl gallate sulfate isomer 1 | **+** | − | − |
| MG3 | 59.29 | C_8_H_8_O_8_S | 262.9850 | −6.46 | methyl gallate sulfate isomer 2 | **+** | − | − |
| MG4 | 61.06 | C_8_H_8_O_8_S | 262.9867 | 0.00 | methyl gallate sulfate isomer 3 | **+** | − | − |
| MG5 | 64.01 | C_8_H_8_O_8_S | 262.9852 | −5.70 | methyl gallate sulfate | **+** | − | − |
| MG6 | 84.88 | C_8_H_8_O_8_S | 262.9862 | −1.90 | methyl gallate sulfate isomer 4 | **+** | − | − |
| MG7 | 88.43 | C_8_H_8_O_8_S | 262.9854 | −4.94 | methyl gallate sulfate isomer 5 | **+** | − | − |
| MG8 | 125.33 | C_8_H_8_O_8_S | 262.9864 | −1.14 | methyl gallate sulfate isomer 6 | **+** | − | − |
| MG9 | 127.18 | C_8_H_8_O_8_S | 262.9887 | 7.60 | methyl gallate sulfate isomer 7 | − | **+** | − |
| MG10 | 36.25 | C_14_H_16_O_11_ | 359.0622 | 0.56 | methyl gallate glucuronide isomer 1 | **+** | − | − |
| MG11 | 37.21 | C_14_H_16_O_11_ | 359.0620 | 0.00 | methyl gallate glucuronide | **+** | − | − |
| MG12 | 50.09 | C_14_H_16_O_11_ | 359.0597 | −6.41 | methyl gallate glucuronide isomer 2 | − | **+** | − |
| MG13 | 26.75 | C_14_H_16_O_11_ | 359.0618 | −0.56 | methyl gallate glucuronide isomer 3 | − | **+** | − |
| MG14 | 40.24 | C_20_H_24_O_17_ | 535.0949 | 1.50 | methyl gallate diglucuronide isomer 1 | **+** | − | − |
| MG15 | 38.77 | C_20_H_24_O_17_ | 535.0943 | 0.37 | methyl gallate diglucuronide isomer 2 | **+** | − | − |
| MG16 | 58.58 | C_14_H_16_O_14_S | 439.0199 | 2.51 | methyl gallate sulfate glucuronide | **+** | − | − |
| MG17 | 42.90 | C_15_H_18_O_11_ | 373.0765 | −2.95 | methylated methyl gallate glucuronide isomer 1 | **+** | − | − |
| MG18 | 44.57 | C_15_H_18_O_11_ | 373.0773 | −0.80 | methylated methyl gallate glucuronide isomer 2 | **+** | − | − |
| MG19 | 43.71 | C_15_H_18_O_11_ | 373.0763 | −3.48 | methylated methyl gallate glucuronide isomer 3 | − | **+** | − |
| MG20 | 76.78 | C_9_H_10_O_8_S | 277.0014 | −3.61 | methylated methyl gallate sulfate isomer 1 | **+** | − | **+** |
| MG21 | 96.53 | C_9_H_10_O_8_S | 277.0015 | −3.25 | methylated methyl gallate sulfate isomer 2 | **+** | − | − |
| MG22 | 126.77 | C_9_H_10_O_8_S | 277.0018 | −2.17 | methylated methyl gallate sulfate isomer 3 | **+** | − | − |
| MG23 | 126.98 | C_10_H_12_O_8_S | 291.0165 | −5.15 | dimethylated methyl gallate sulfate | **+** | − | − |
| MG24 | 44.24 | C_8_H_8_O_7_S | 246.9924 | 2.43 | 3,4-dihydroxy phenylacetic acid sulfate isomer 3 | **+** | − | − |
| Paeoniae Radix Rubra | | | | | | | | |
| PRR1 | 78.72 | C_10_H_16_O_4_ | 199.0978 | 1.00 | paeonimetabolin II isomer 2 | **+** | − | − |
| PRR2 | 80.91 | C_10_H_16_O_4_ | 199.0980 | 2.01 | paeonimetabolin II isomer 3 | **+** | − | − |
| PRR3 | 53.31 | C_16_H_26_O_10_ | 377.1443 | −2.65 | C_10_H_18_O_4_ glucuronide isomer 6 | **+** | − | − |
| PRR4 | 111.38 | C_10_H_18_O_6_S | 265.0740 | −4.15 | 2,6-dihydroxycineol sulfate isomer 8 | − | − | **+** |
| PRR5 | 128.78 | C_10_H_18_O_6_S | 265.0753 | 0.75 | 2,6-dihydroxycineol sulfate isomer 9 | **+** | − | − |
| PRR6 | 57.88 | C_10_H_20_O_6_S | 267.0901 | −2.62 | hydrogenated 2,6-dihydroxycineol sulfate isomer 2 | − | − | **+** |
| PRR7 | 69.08 | C_16_H_26_O_9_ | 361.1522 | 4.98 | paeonimetabolin II glucoside isomer 1 | **+** | − | − |
| PRR8 | 71.20 | C_16_H_26_O_9_ | 361.1486 | −4.98 | paeonimetabolin II glucoside isomer 2 | **+** | − | − |
| PRR9 | 72.81 | C_16_H_26_O_9_ | 361.1508 | 1.11 | paeonimetabolin II glucoside isomer 4 | **+** | − | − |
| PRR10 | 26.15 | C_17_H_26_O_10_ | 435.1477 | −7.12 | methyl debenzoyl paeoniflorin isomer 2 | − | **+** | − |
| PRR11 | 70.89 | C_23_H_28_O_10_ | 509.1679 | 2.75 | hydrogenated lactiflorin | **+** | − | − |
| PRR12 | 35.33 | C_23_H_28_O_11_ | 525.1635 | 4.00 | hydrogenated hydroxylated lactiflorin isomer 1 | − | − | **+** |
| PRR13 | 33.67 | C_23_H_28_O_11_ | 525.1624 | 1.90 | hydrogenated hydroxylated lactiflorin isomer 2 | − | − | **+** |
| PRR14 | 37.32 | C_23_H_28_O_11_ | 525.1624 | 1.90 | hydrogenated hydroxylated lactiflorin isomer 3 | − | − | **+** |
| PRR15 | 95.61 | C_15_H_16_O_6_ | 291.0866 | −2.75 | 3,4-diHPP-2-ol isomer 1 | **+** | − | − |
| PRR16 | 98.63 | C_15_H_16_O_6_ | 291.0875 | 0.34 | 3,4-diHPP-2-ol isomer 2 | **+** | − | − |
| PRR17 | 102.43 | C_15_H_16_O_6_ | 291.0863 | −3.78 | 3,4-diHPP-2-ol isomer 3 | **+** | − | − |
| PRR18 | 108.17 | C_15_H_16_O_6_ | 291.0885 | 3.78 | 3,4-diHPP-2-ol isomer 4 | **+** | − | − |
| PRR19 | 110.64 | C_15_H_16_O_6_ | 291.0880 | 2.06 | 3,4-diHPP-2-ol isomer 5 | **+** | − | − |
| PRR20 | 125.63 | C_15_H_16_O_6_ | 291.0875 | 0.34 | 3,4-diHPP-2-ol isomer 6 | **+** | − | − |
| PRR21 | 124.59 | C_12_H_16_O_8_S | 319.0488 | −1.57 | dihydroxylated methoxylated benzenepentanoic acid sulfate isomer 4 | **+** | − | − |
| PRR22 | 48.00 | C_8_H_8_O_6_S | 230.9960 | −3.90 | 3/4-hydroxy phenylacetic acid sulfate isomer 5 | **+** | − | − |
| PRR23 | 43.92 | C_8_H_8_O_6_S | 230.9962 | −3.03 | 3/4-hydroxy phenylacetic acid sulfate isomer 6 | **+** | − | − |
| PRR24 | 40.17 | C_9_H_10_O_7_S | 261.0082 | 3.07 | 3,4-dihydroxy phenylpropionic acid sulfate isomer 5 | **+** | − | − |
| PRR25 | 53.09 | C_9_H_10_O_7_S | 261.0062 | −4.60 | 3,4-dihydroxy phenylpropionic acid sulfate isomer 6 | **+** | − | − |
| PRR26 | 41.72 | C_8_H_8_O_7_S | 246.9910 | −3.24 | 3,4-dihydroxy phenylacetic acid sulfate isomer 5 | **+** | − | − |
| PRR27 | 40.62 | C_7_H_6_O_6_S | 216.9809 | −1.38 | 3/4-hydroxy benzonic acid sulfate isomer 2 | **+** | − | − |
| PRR28 | 129.02 | C_13_H_8_O_6_S | 290.9963 | −2.06 | urolithin B sulfate isomer 2 | − | − | **+** |
| PRR29 | 116.13 | C_13_H_8_O_6_S | 290.9947 | −7.65 | urolithin B sulfate isomer 3 | − | − | **+** |
| PRR30 | 37.30 | C_9_H_9_NO_3_ | 178.0500 | −5.62 | hippuric acid | **+** | − | − |
| PRR31 | 81.24 | C_7_H_8_O_4_S | 187.0064 | −3.74 | benzyl alcohol sulfate | **+** | − | − |
