# Supplementary material for: Metabolism of Paeoniae Radix Rubra and its 14 constituents in mice
Source: Front Pharmacol. 2022 Oct 4;13:995641. doi: 10.3389/fphar.2022.995641 (PMC9577399; doi:10.3389/fphar.2022.995641)
Supplement: Supplementary file 3 [file DataSheet1.PDF]

**Supplementary material**

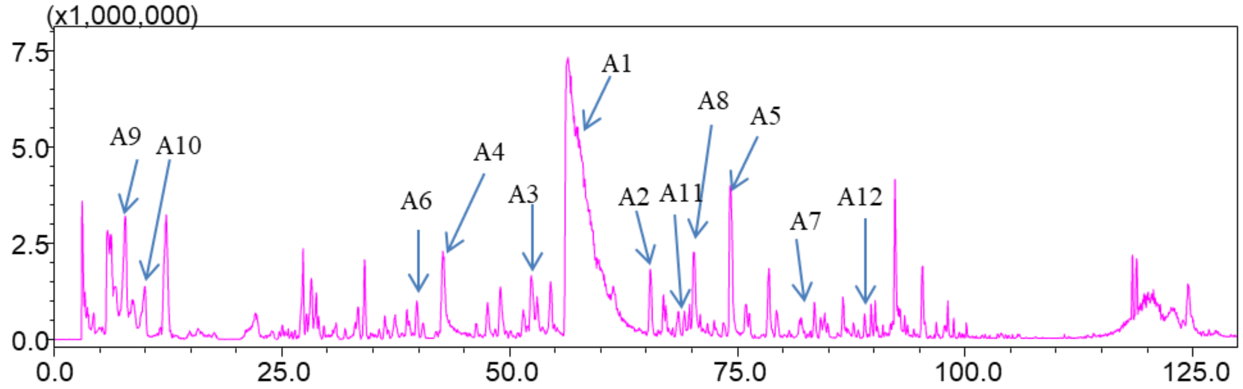


Figure S1. Base peak chromatogram (BPC) of Paeonia Radix Rubra decoction dried powder detected in negative ion detection mode. A1, paeoniflorin; A2, paeoniflorin isomer; A3, albiflorin; A4, oxypaeoniflorin; A5, benzoylpaeoniflorin; A6, catechin; A7, benzoyloxypaeoniflorin; A8, galloylpaeoniflorin; A9 and A10, desbenzoylpaeoniflin and isomer; A11, ellagic acid; A12, dimethyl ellagic acid.

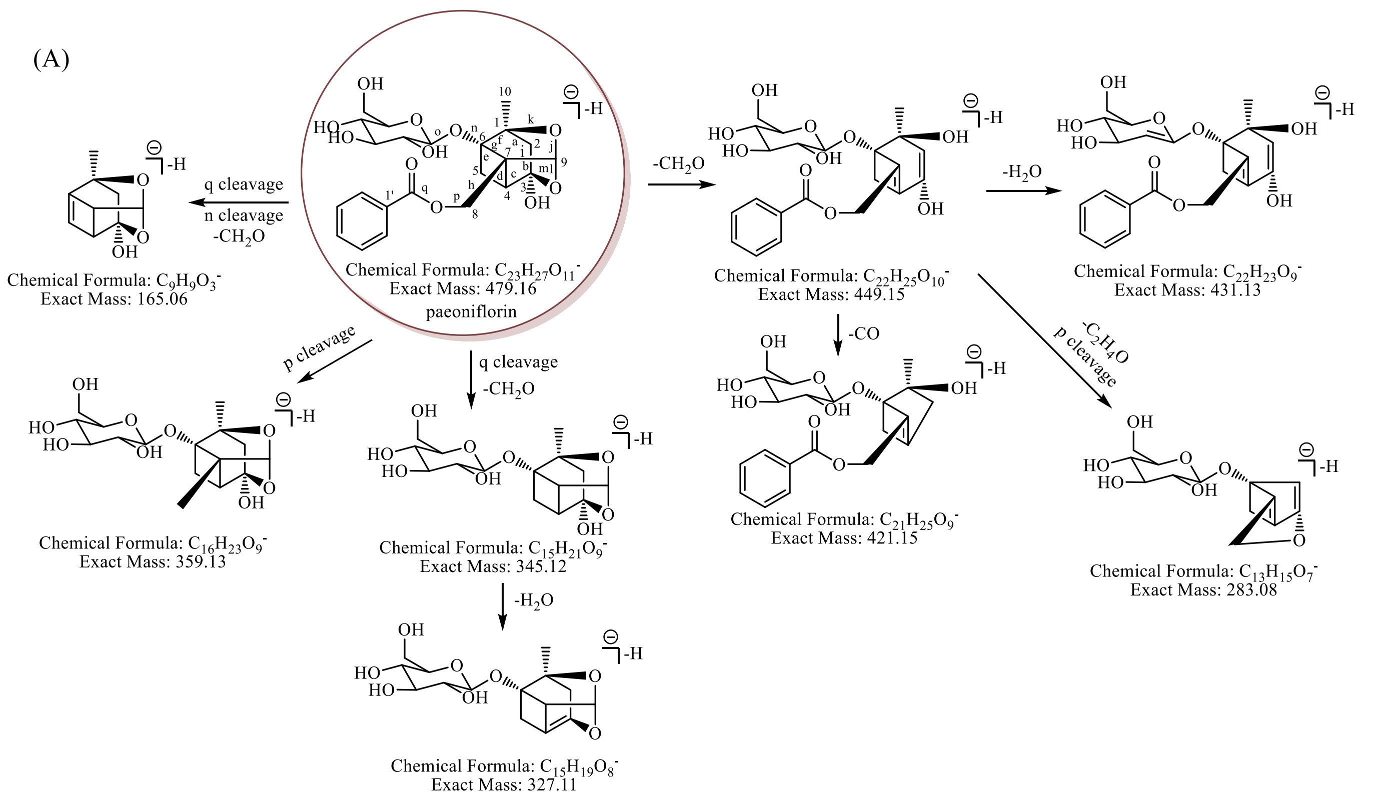

(A)
q cleavage
n cleavage
-CH2O
Chemical Formula: C9H9O3-
Exact Mass: 165.06
Chemical Formula: C23H27O11-
Exact Mass: 479.16
paeoniflorin
-CH2O
Chemical Formula: C22H25O10-
Exact Mass: 449.15
-H2O
Chemical Formula: C22H23O9-
Exact Mass: 431.13
p cleavage
Chemical Formula: C16H23O9-
Exact Mass: 359.13
q cleavage
-CH2O
Chemical Formula: C15H21O9-
Exact Mass: 345.12
-H2O
Chemical Formula: C15H19O8-
Exact Mass: 327.11
-CO
Chemical Formula: C21H25O9-
Exact Mass: 421.15
-C2H4O
p cleavage
Chemical Formula: C13H15O7-
Exact Mass: 283.08

(B)

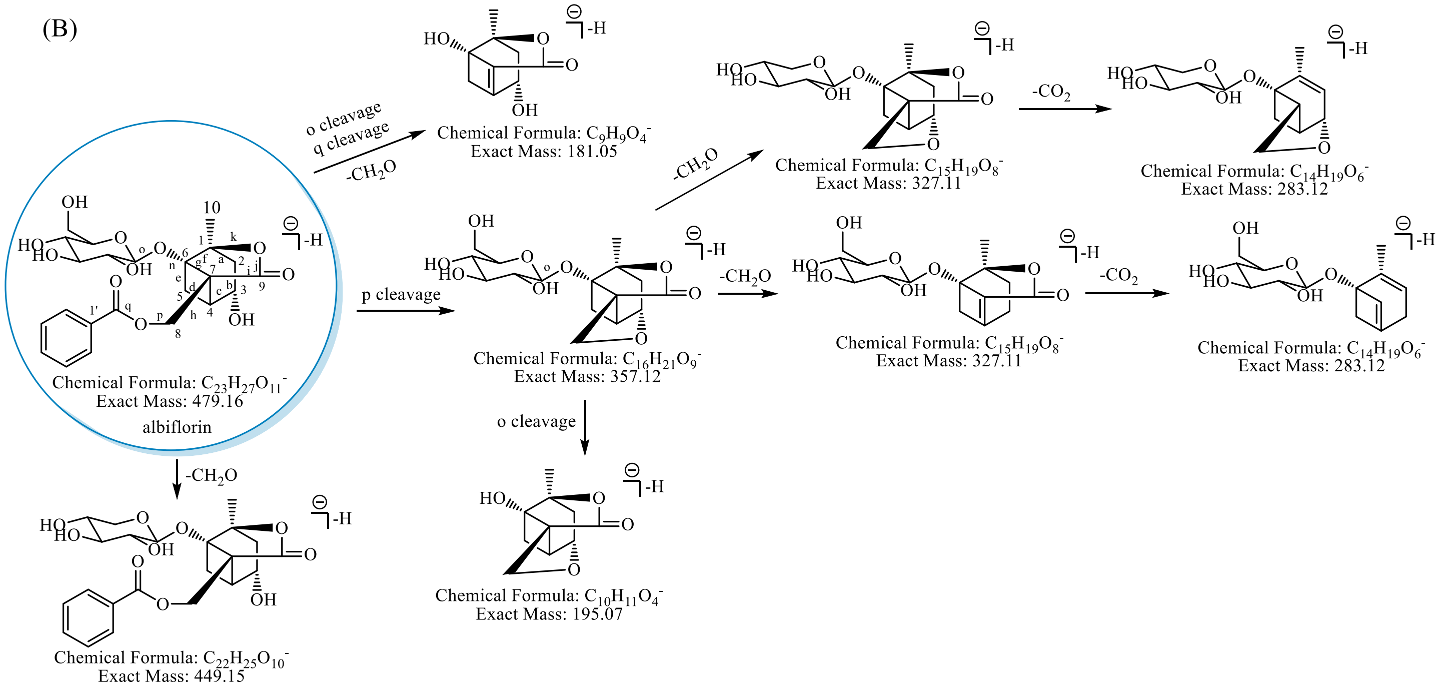
o cleavage
q cleavage
-CH2O
Chemical Formula: C9H9O4-
Exact Mass: 181.05
Chemical Formula: C23H27O11-
Exact Mass: 479.16
albiflorin
p cleavage
Chemical Formula: C16H21O9-
Exact Mass: 357.12
-CH2O
Chemical Formula: C15H19O8-
Exact Mass: 327.11
-CO2
Chemical Formula: C14H19O6-
Exact Mass: 283.12
-CH2O
Chemical Formula: C15H19O8-
Exact Mass: 327.11
-CO2
Chemical Formula: C14H19O6-
Exact Mass: 283.12
o cleavage
Chemical Formula: C10H11O4-
Exact Mass: 195.07
-CH2O
Chemical Formula: C22H25O10-
Exact Mass: 449.15

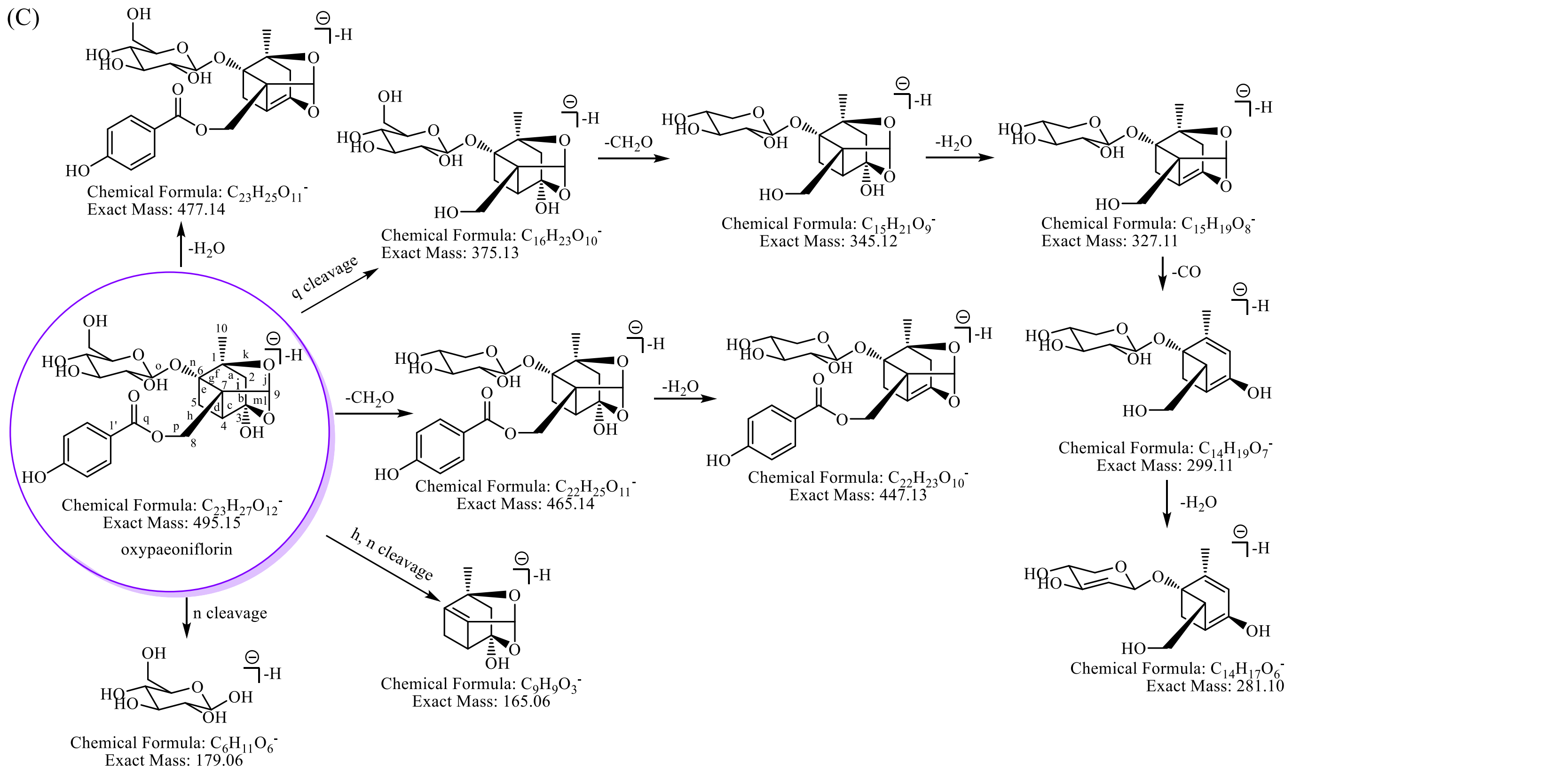
(C)
Chemical Formula: $C_{23}H_{25}O_{11}^-$
Exact Mass: 477.14
-H2O
q cleavage
Chemical Formula: $C_{16}H_{23}O_{10}^-$
Exact Mass: 375.13
-CH2O
Chemical Formula: $C_{15}H_{21}O_9^-$
Exact Mass: 345.12
-H2O
Chemical Formula: $C_{15}H_{19}O_8^-$
Exact Mass: 327.11
-CO
Chemical Formula: $C_{14}H_{19}O_7^-$
Exact Mass: 299.11
-H2O
Chemical Formula: $C_{14}H_{17}O_6^-$
Exact Mass: 281.10
Chemical Formula: $C_{23}H_{27}O_{12}^-$
Exact Mass: 495.15
oxypaeoniflorin
-CH2O
Chemical Formula: $C_{22}H_{25}O_{11}^-$
Exact Mass: 465.14
-H2O
Chemical Formula: $C_{22}H_{23}O_{10}^-$
Exact Mass: 447.13
h, n cleavage
Chemical Formula: $C_9H_9O_3^-$
Exact Mass: 165.06
n cleavage
Chemical Formula: $C_6H_{11}O_6^-$
Exact Mass: 179.06

(D)

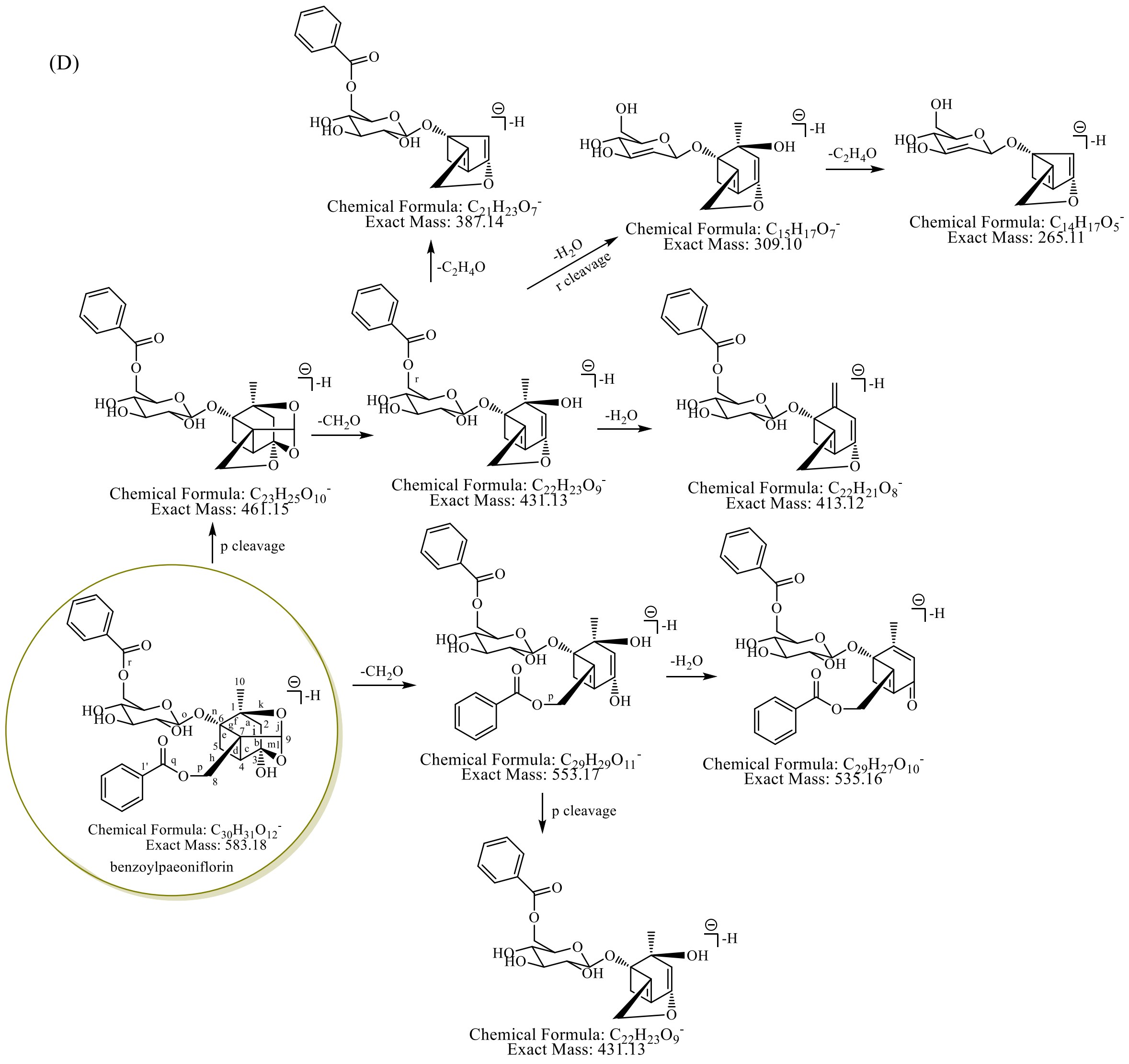

Chemical Formula: $C_{21}H_{23}O_7^-$
Exact Mass: 387.14
Chemical Formula: $C_{15}H_{17}O_7^-$
Exact Mass: 309.10
Chemical Formula: $C_{14}H_{17}O_5^-$
Exact Mass: 265.11
-$C_2H_4O$
-$H_2O$
r cleavage
-$C_2H_4O$
Chemical Formula: $C_{23}H_{25}O_{10}^-$
Exact Mass: 461.15
-$CH_2O$
Chemical Formula: $C_{22}H_{23}O_9^-$
Exact Mass: 431.13
-$H_2O$
Chemical Formula: $C_{22}H_{21}O_8^-$
Exact Mass: 413.12
p cleavage
-$CH_2O$
Chemical Formula: $C_{29}H_{29}O_{11}^-$
Exact Mass: 553.17
-$H_2O$
Chemical Formula: $C_{29}H_{27}O_{10}^-$
Exact Mass: 535.16
Chemical Formula: $C_{30}H_{31}O_{12}^-$
Exact Mass: 583.18
benzoylpaeoniflorin
p cleavage
Chemical Formula: $C_{22}H_{23}O_9^-$
Exact Mass: 431.13

(E)

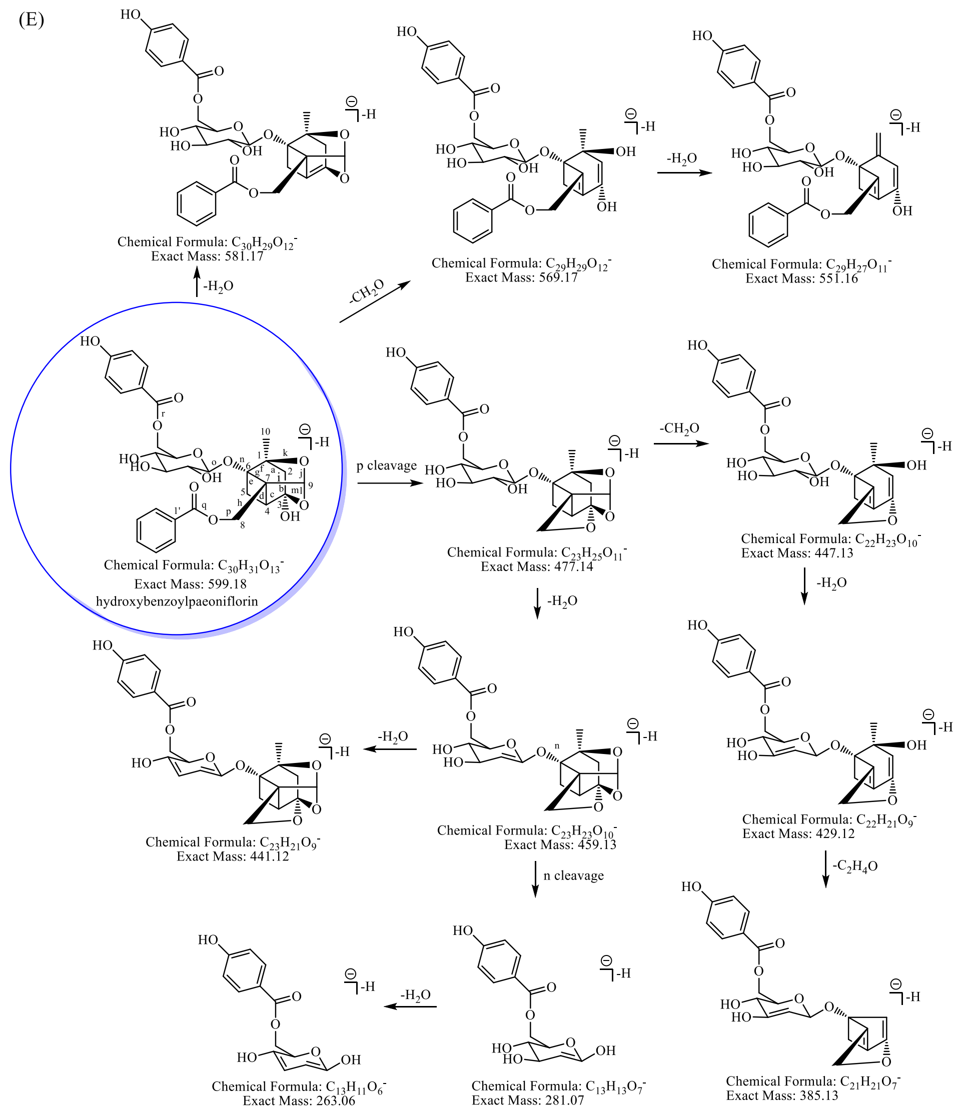

Chemical Formula: $C_{30}H_{29}O_{12}^-$
Exact Mass: 581.17
-H2O
-CH2O
Chemical Formula: $C_{29}H_{29}O_{12}^-$
Exact Mass: 569.17
-H2O
Chemical Formula: $C_{29}H_{27}O_{11}^-$
Exact Mass: 551.16
Chemical Formula: $C_{30}H_{31}O_{13}^-$
Exact Mass: 599.18
hydroxybenzoylpaeoniflorin
p cleavage
Chemical Formula: $C_{23}H_{25}O_{11}^-$
Exact Mass: 477.14
-CH2O
Chemical Formula: $C_{22}H_{23}O_{10}^-$
Exact Mass: 447.13
-H2O
-H2O
Chemical Formula: $C_{23}H_{21}O_{9}^-$
Exact Mass: 441.12
-H2O
Chemical Formula: $C_{23}H_{23}O_{10}^-$
Exact Mass: 459.13
Chemical Formula: $C_{22}H_{21}O_{9}^-$
Exact Mass: 429.12
n cleavage
-C2H4O
Chemical Formula: $C_{13}H_{11}O_{6}^-$
Exact Mass: 263.06
-H2O
Chemical Formula: $C_{13}H_{13}O_{7}^-$
Exact Mass: 281.07
Chemical Formula: $C_{21}H_{21}O_{7}^-$
Exact Mass: 385.13

(F)

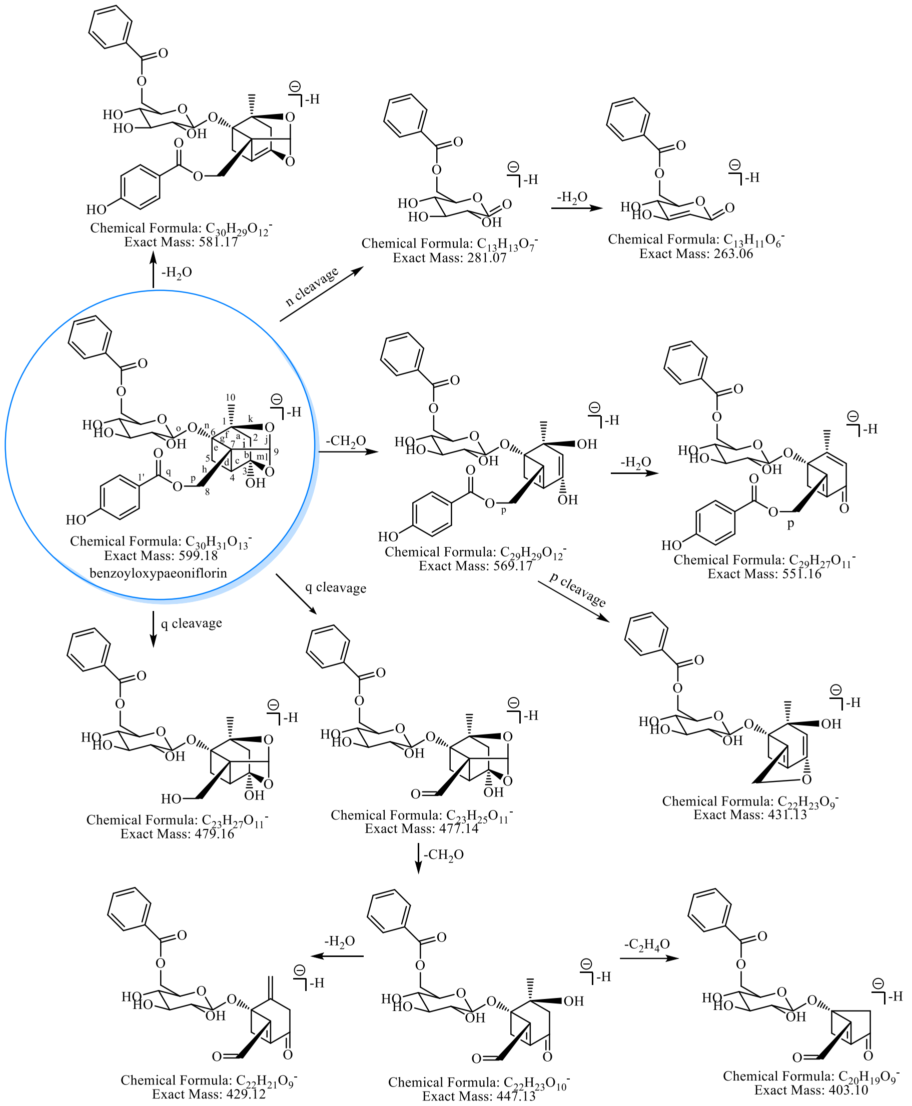
Chemical Formula: C30H29O12-
Exact Mass: 581.17
-H2O
n cleavage
Chemical Formula: C13H13O7-
Exact Mass: 281.07
-H2O
Chemical Formula: C13H11O6-
Exact Mass: 263.06
Chemical Formula: C30H31O13-
Exact Mass: 599.18
benzoyloxypaeoniflorin
-CH2O
Chemical Formula: C29H29O12-
Exact Mass: 569.17
-H2O
Chemical Formula: C29H27O11-
Exact Mass: 551.16
q cleavage
q cleavage
p cleavage
Chemical Formula: C23H27O11-
Exact Mass: 479.16
Chemical Formula: C23H25O11-
Exact Mass: 477.14
Chemical Formula: C22H23O9-
Exact Mass: 431.13
-CH2O
-H2O
-C2H4O
Chemical Formula: C22H21O9-
Exact Mass: 429.12
Chemical Formula: C22H23O10-
Exact Mass: 447.13
Chemical Formula: C20H19O9-
Exact Mass: 403.10

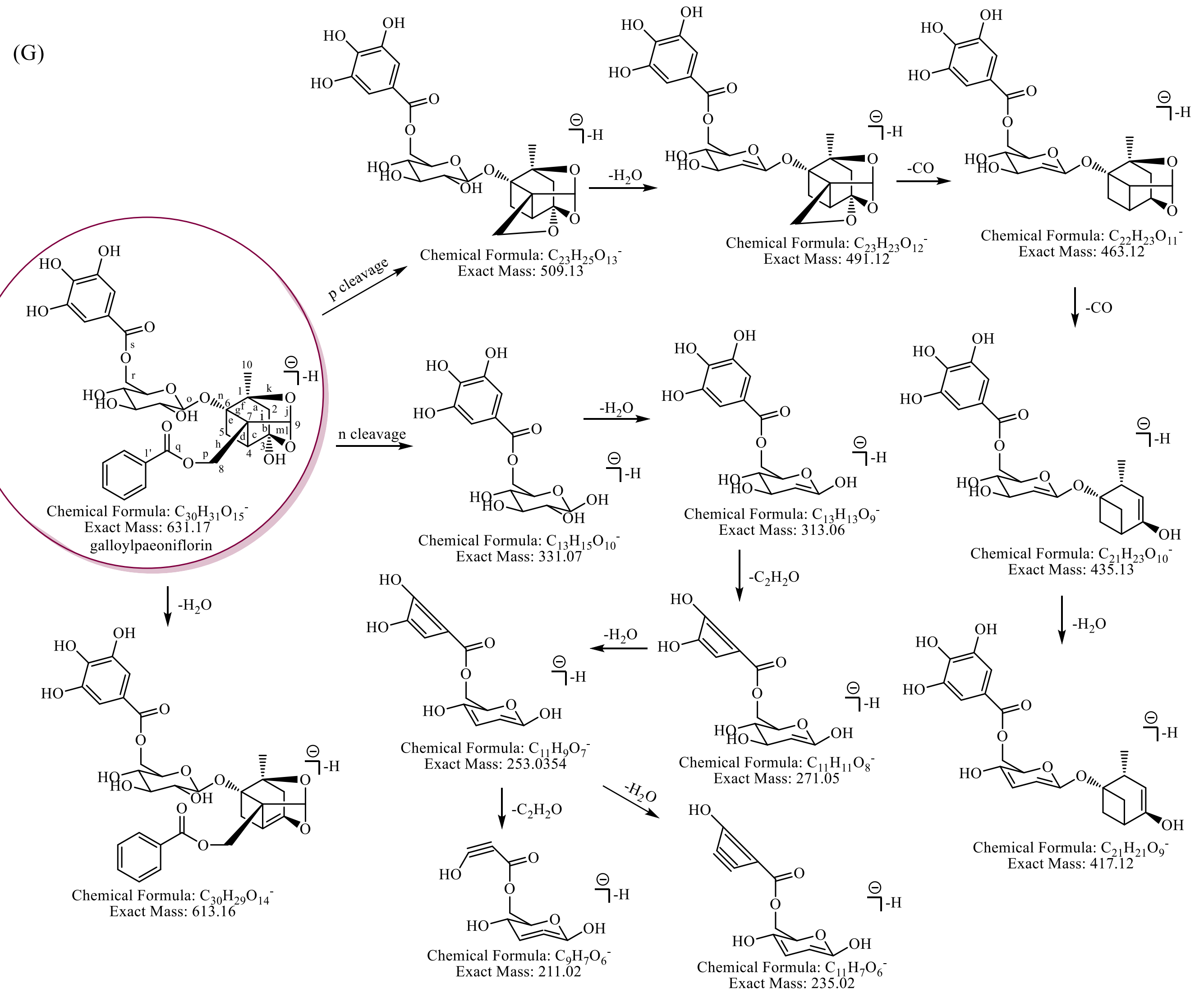
(G)
p cleavage
n cleavage
Chemical Formula: $C_{30}H_{31}O_{15}^-$
Exact Mass: 631.17
galloylpaeoniflorin
Chemical Formula: $C_{23}H_{25}O_{13}^-$
Exact Mass: 509.13
-$H_2O$
Chemical Formula: $C_{23}H_{23}O_{12}^-$
Exact Mass: 491.12
-CO
Chemical Formula: $C_{22}H_{23}O_{11}^-$
Exact Mass: 463.12
-CO
Chemical Formula: $C_{21}H_{23}O_{10}^-$
Exact Mass: 435.13
-$H_2O$
Chemical Formula: $C_{21}H_{21}O_9^-$
Exact Mass: 417.12
Chemical Formula: $C_{13}H_{15}O_{10}^-$
Exact Mass: 331.07
-$H_2O$
Chemical Formula: $C_{13}H_{13}O_9^-$
Exact Mass: 313.06
-$C_2H_2O$
Chemical Formula: $C_{11}H_{11}O_8^-$
Exact Mass: 271.05
-$H_2O$
Chemical Formula: $C_{11}H_9O_7^-$
Exact Mass: 253.0354
-$C_2H_2O$
Chemical Formula: $C_9H_7O_6^-$
Exact Mass: 211.02
-$H_2O$
Chemical Formula: $C_{11}H_7O_6^-$
Exact Mass: 235.02
-$H_2O$
Chemical Formula: $C_{30}H_{29}O_{14}^-$
Exact Mass: 613.16

(H)

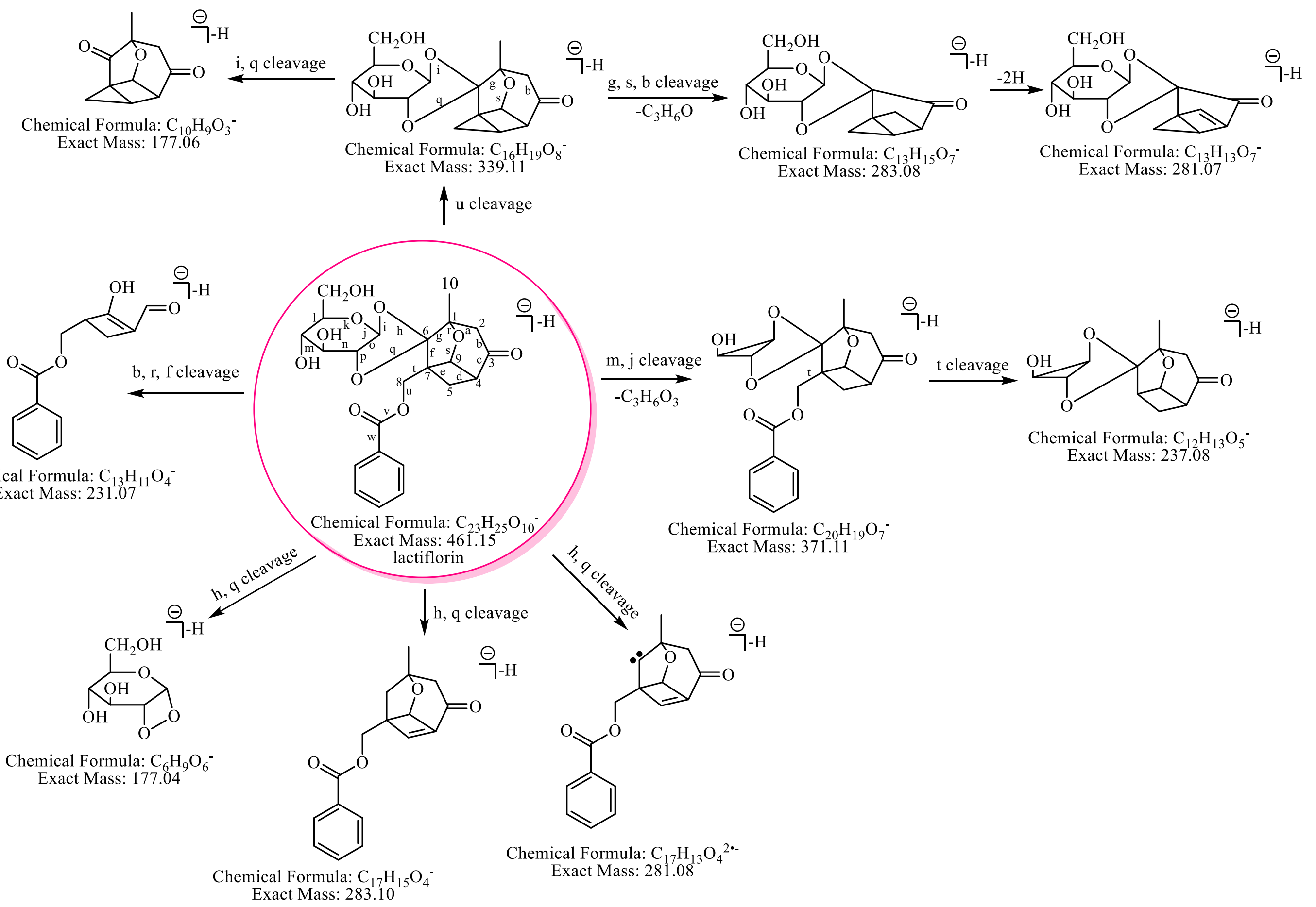

i, q cleavage
Chemical Formula: C10H9O3-
Exact Mass: 177.06
Chemical Formula: C16H19O8-
Exact Mass: 339.11
g, s, b cleavage
-C3H6O
Chemical Formula: C13H15O7-
Exact Mass: 283.08
-2H
Chemical Formula: C13H13O7-
Exact Mass: 281.07
u cleavage
b, r, f cleavage
Chemical Formula: C13H11O4-
Exact Mass: 231.07
Chemical Formula: C23H25O10-
Exact Mass: 461.15
lactiflorin
m, j cleavage
-C3H6O3
Chemical Formula: C20H19O7-
Exact Mass: 371.11
t cleavage
Chemical Formula: C12H13O5-
Exact Mass: 237.08
h, q cleavage
Chemical Formula: C6H9O6-
Exact Mass: 177.04
h, q cleavage
Chemical Formula: C17H15O4-
Exact Mass: 283.10
h, q cleavage
Chemical Formula: C17H13O42•-
Exact Mass: 281.08

(I)

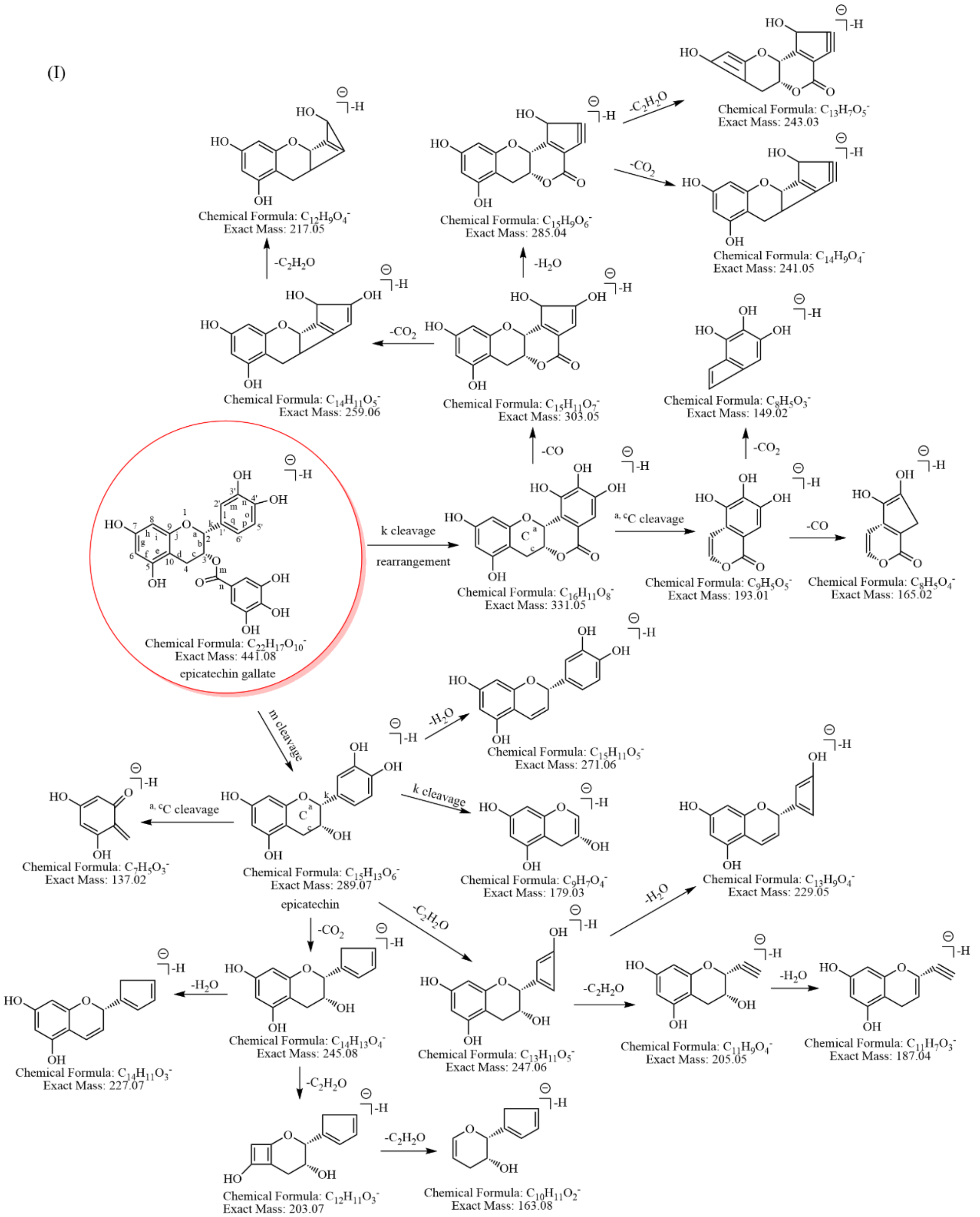

Chemical Formula: C13H7O5-
Exact Mass: 243.03
-C2H2O
Chemical Formula: C12H9O4-
Exact Mass: 217.05
Chemical Formula: C15H9O6-
Exact Mass: 285.04
-CO2
Chemical Formula: C14H9O4-
Exact Mass: 241.05
-C2H2O
-H2O
-CO2
Chemical Formula: C14H11O5-
Exact Mass: 259.06
Chemical Formula: C15H11O7-
Exact Mass: 303.05
Chemical Formula: C8H5O3-
Exact Mass: 149.02
-CO
-CO2
k cleavage
rearrangement
Chemical Formula: C22H17O10-
Exact Mass: 441.08
epicatechin gallate
Chemical Formula: C16H11O8-
Exact Mass: 331.05
a, cC cleavage
Chemical Formula: C9H5O5-
Exact Mass: 193.01
-CO
Chemical Formula: C8H5O4-
Exact Mass: 165.02
m cleavage
-H2O
Chemical Formula: C15H11O5-
Exact Mass: 271.06
a, cC cleavage
Chemical Formula: C7H5O3-
Exact Mass: 137.02
Chemical Formula: C15H13O6-
Exact Mass: 289.07
epicatechin
k cleavage
Chemical Formula: C9H7O4-
Exact Mass: 179.03
-H2O
Chemical Formula: C13H9O4-
Exact Mass: 229.05
-CO2
-C2H2O
-H2O
Chemical Formula: C14H11O3-
Exact Mass: 227.07
Chemical Formula: C14H13O4-
Exact Mass: 245.08
Chemical Formula: C13H11O5-
Exact Mass: 247.06
-C2H2O
Chemical Formula: C11H9O4-
Exact Mass: 205.05
-H2O
Chemical Formula: C11H7O3-
Exact Mass: 187.04
-C2H2O
Chemical Formula: C12H11O3-
Exact Mass: 203.07
-C2H2O
Chemical Formula: C10H11O2-
Exact Mass: 163.08

(J)

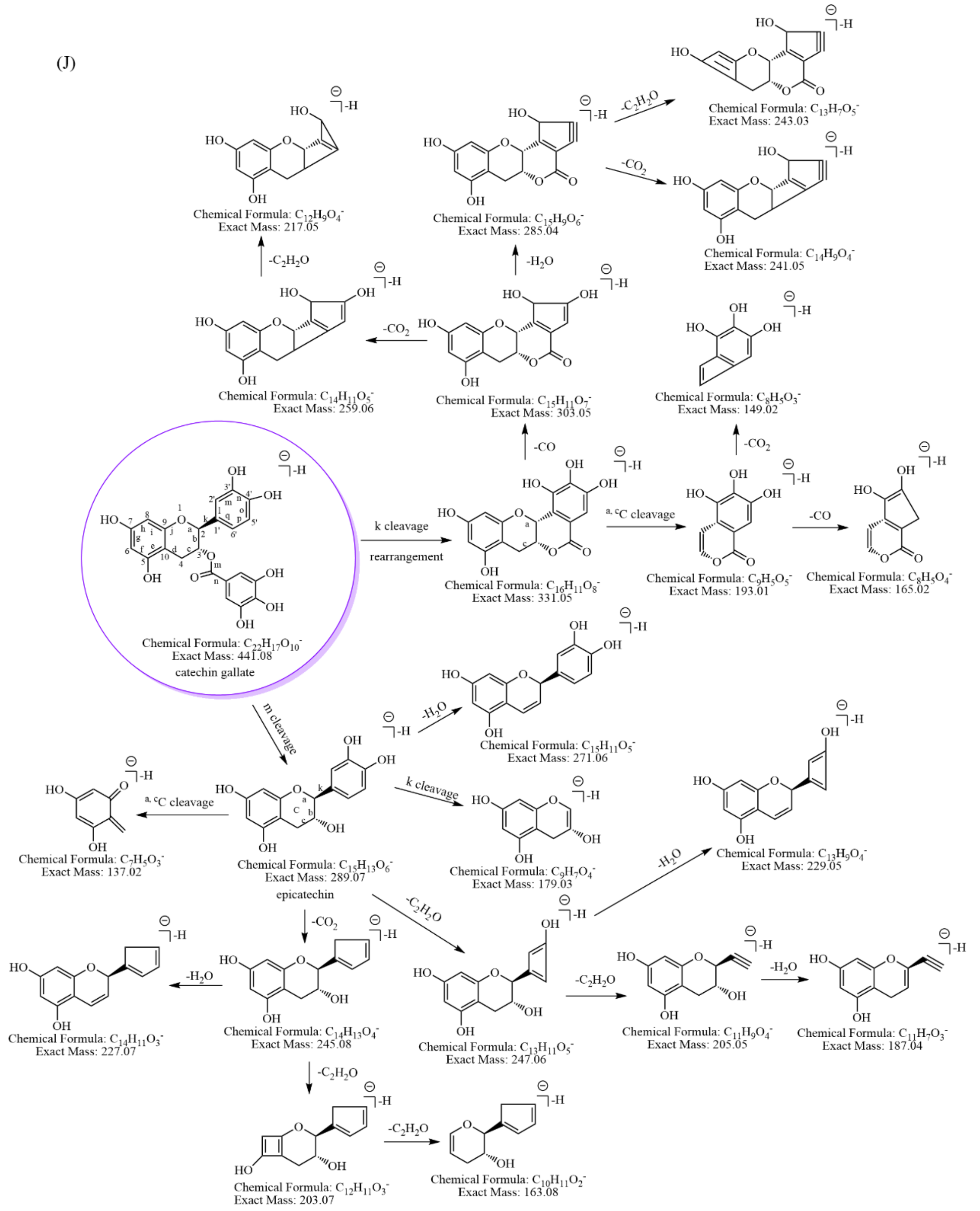

Chemical Formula: C22H17O10-
Exact Mass: 441.08
catechin gallate
k cleavage
rearrangement
Chemical Formula: C16H11O8-
Exact Mass: 331.05
-CO
Chemical Formula: C15H11O7-
Exact Mass: 303.05
-H2O
Chemical Formula: C15H9O6-
Exact Mass: 285.04
-C2H2O
Chemical Formula: C13H7O5-
Exact Mass: 243.03
-CO2
Chemical Formula: C14H9O4-
Exact Mass: 241.05
-CO2
Chemical Formula: C14H11O5-
Exact Mass: 259.06
-C2H2O
Chemical Formula: C12H9O4-
Exact Mass: 217.05
a, cC cleavage
Chemical Formula: C9H5O5-
Exact Mass: 193.01
-CO2
Chemical Formula: C8H5O3-
Exact Mass: 149.02
-CO
Chemical Formula: C8H5O4-
Exact Mass: 165.02
m cleavage
Chemical Formula: C15H13O6-
Exact Mass: 289.07
epicatechin
-H2O
Chemical Formula: C15H11O5-
Exact Mass: 271.06
k cleavage
Chemical Formula: C9H7O4-
Exact Mass: 179.03
a, cC cleavage
Chemical Formula: C7H5O3-
Exact Mass: 137.02
-CO2
Chemical Formula: C14H13O4-
Exact Mass: 245.08
-H2O
Chemical Formula: C14H11O3-
Exact Mass: 227.07
-C2H2O
Chemical Formula: C12H11O3-
Exact Mass: 203.07
-C2H2O
Chemical Formula: C10H11O2-
Exact Mass: 163.08
-C2H2O
Chemical Formula: C13H11O5-
Exact Mass: 247.06
-H2O
Chemical Formula: C13H9O4-
Exact Mass: 229.05
-C2H2O
Chemical Formula: C11H9O4-
Exact Mass: 205.05
-H2O
Chemical Formula: C11H7O3-
Exact Mass: 187.04

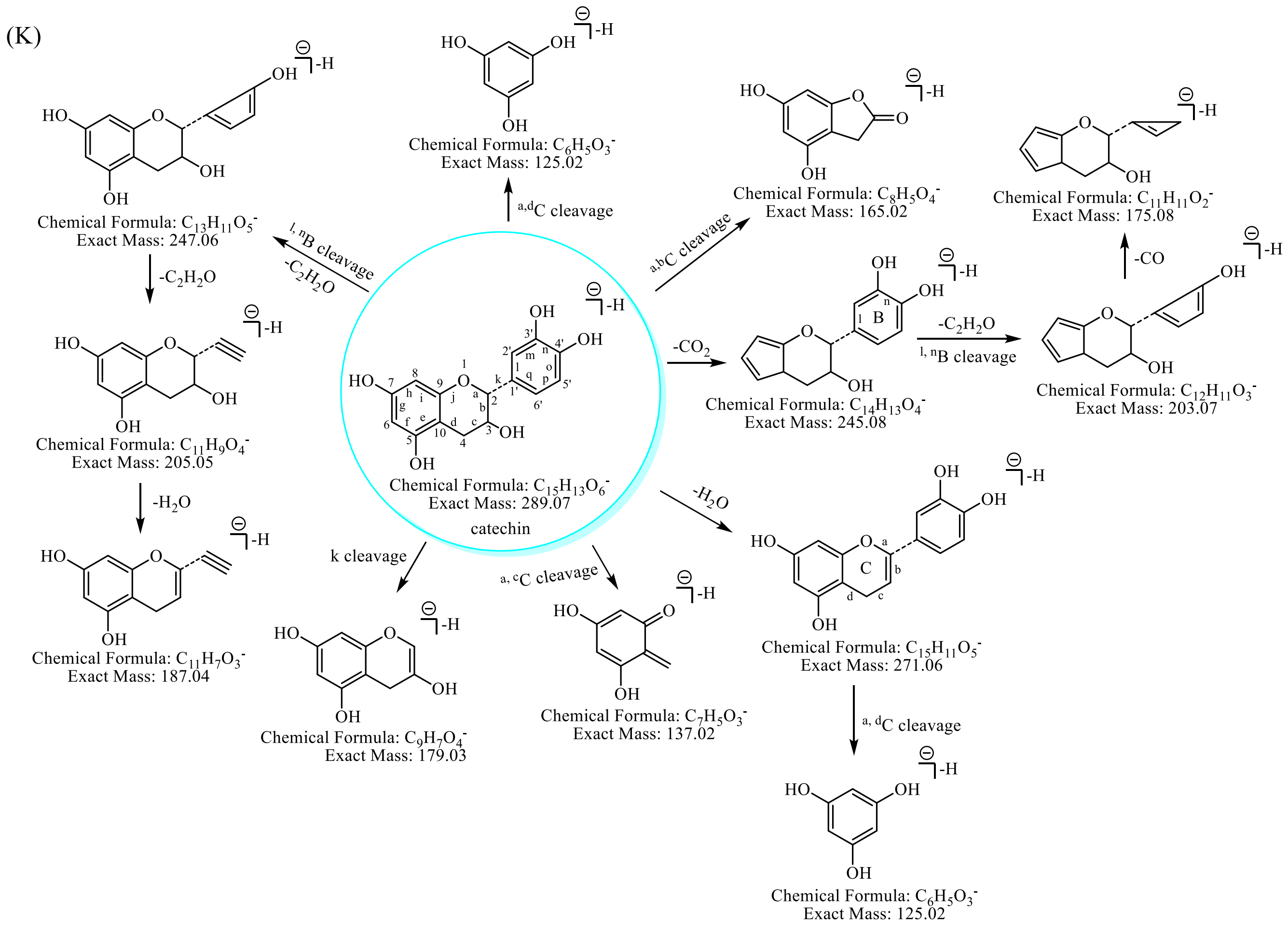

(K)
Chemical Formula: $C_{13}H_{11}O_5^-$
Exact Mass: 247.06
-$C_2H_2O$
Chemical Formula: $C_{11}H_9O_4^-$
Exact Mass: 205.05
-$H_2O$
Chemical Formula: $C_{11}H_7O_3^-$
Exact Mass: 187.04
$^{l,n}$B cleavage
-$C_2H_2O$
Chemical Formula: $C_6H_5O_3^-$
Exact Mass: 125.02
$^{a,d}$C cleavage
Chemical Formula: $C_8H_5O_4^-$
Exact Mass: 165.02
$^{a,b}$C cleavage
Chemical Formula: $C_{11}H_{11}O_2^-$
Exact Mass: 175.08
-CO
Chemical Formula: $C_{15}H_{13}O_6^-$
Exact Mass: 289.07
catechin
-$CO_2$
Chemical Formula: $C_{14}H_{13}O_4^-$
Exact Mass: 245.08
-$C_2H_2O$
$^{l,n}$B cleavage
Chemical Formula: $C_{12}H_{11}O_3^-$
Exact Mass: 203.07
-$H_2O$
Chemical Formula: $C_{15}H_{11}O_5^-$
Exact Mass: 271.06
$^{a,d}$C cleavage
Chemical Formula: $C_6H_5O_3^-$
Exact Mass: 125.02
k cleavage
Chemical Formula: $C_9H_7O_4^-$
Exact Mass: 179.03
$^{a,c}$C cleavage
Chemical Formula: $C_7H_5O_3^-$
Exact Mass: 137.02

(L)

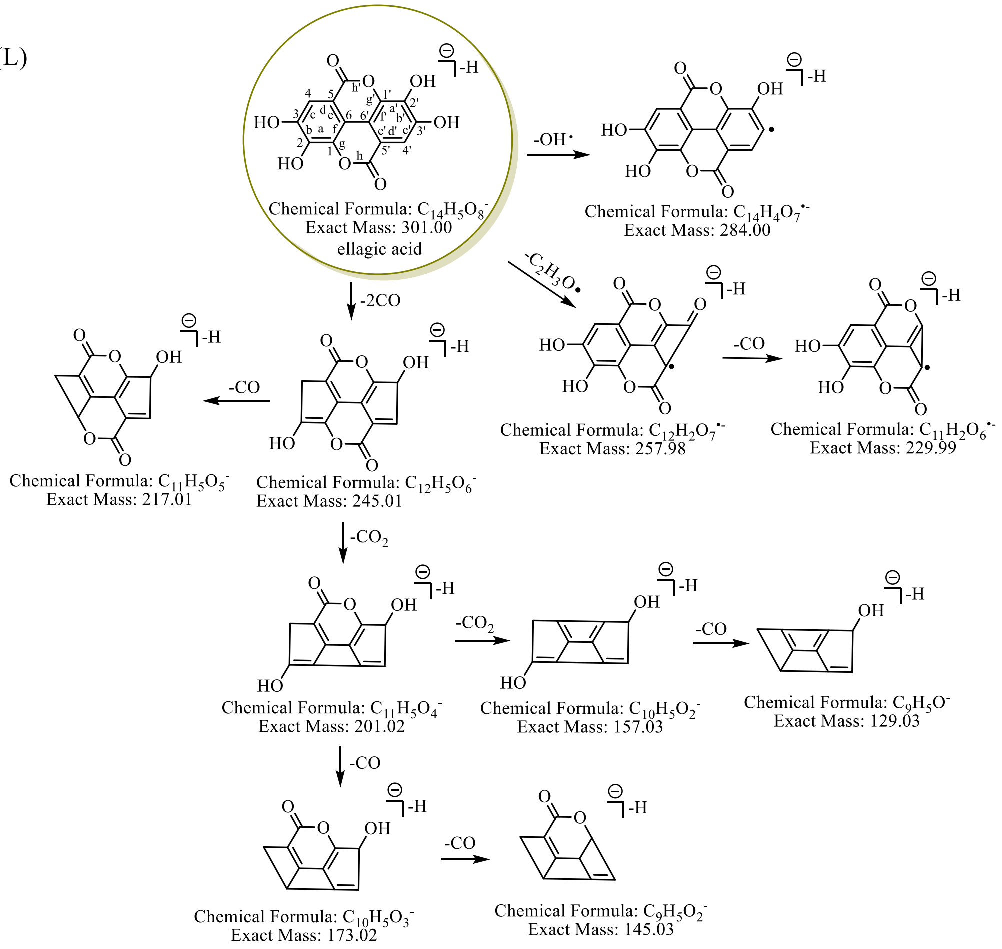


(M)

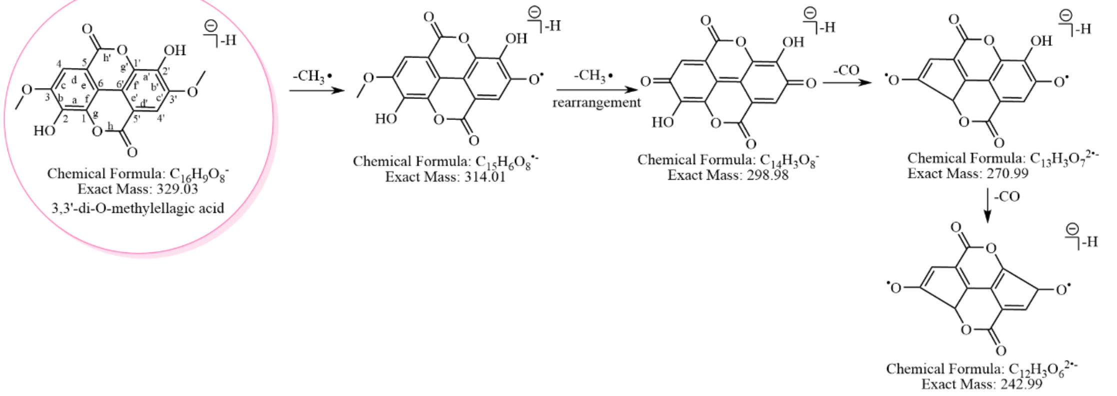


(N)

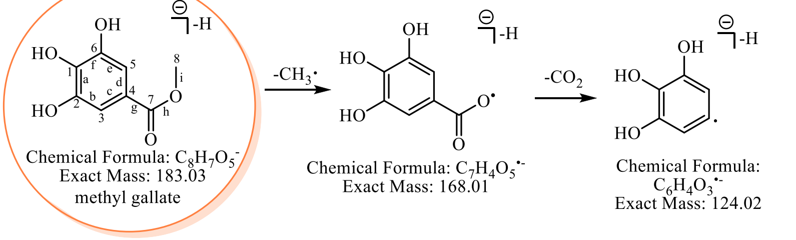


Figure S2. A, B, C, D, E, F, G, H, I, J, K, L, M, and N represent the proposed fragmentation pathways of paeoniflorin, albiflorin, oxypaeoniflorin, benzoylpaeoniflorin, hydroxybenzoylpaeoniflorin, benzoyloxypaeoniflorin, galloylpaeoniflorin, lactiflorin, epicatechin gallate, catechin gallate,

catechin, ellagic acid, 3,3'-di-*O*-methyl ellagic acid, methyl gallate.

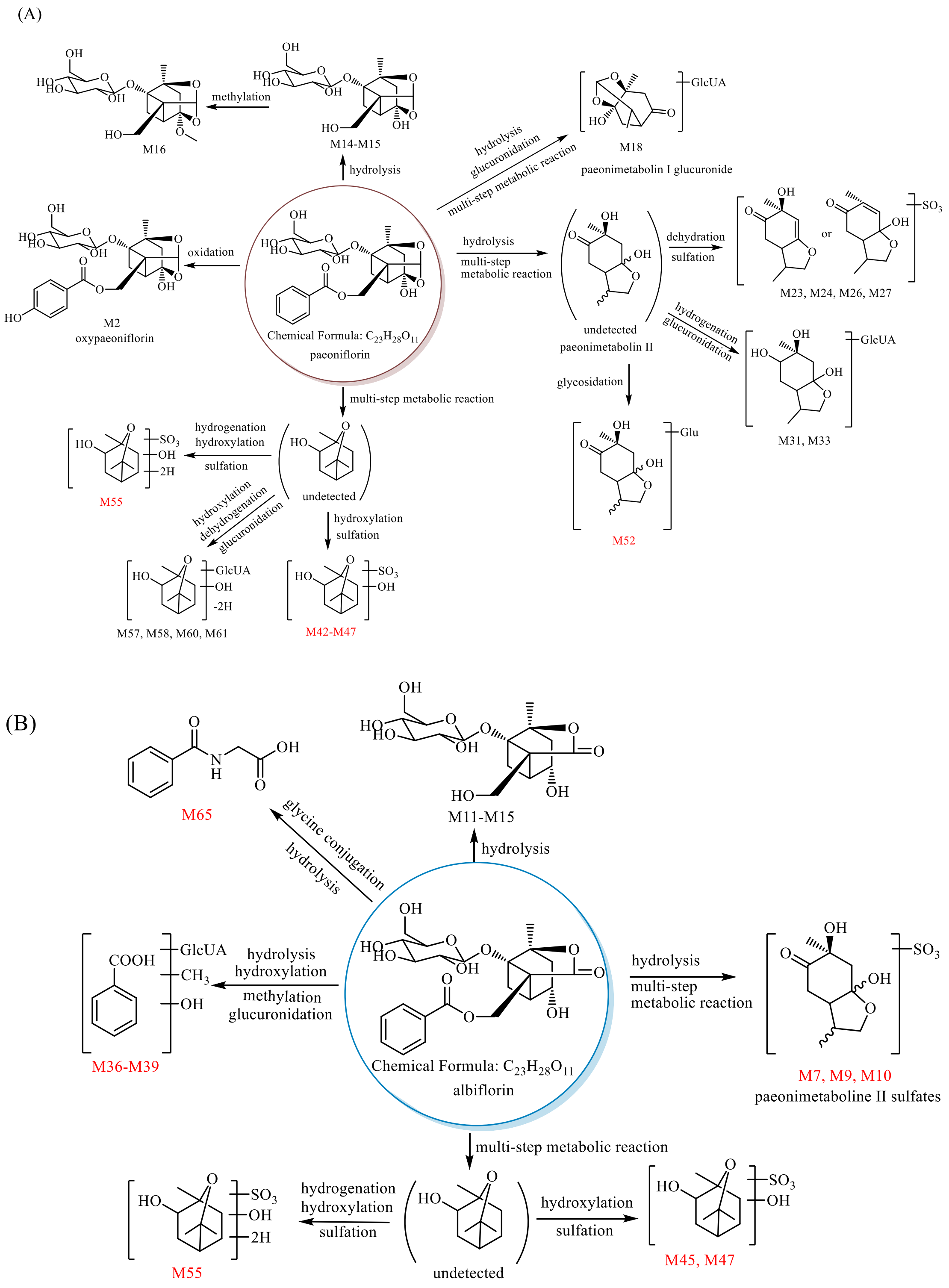

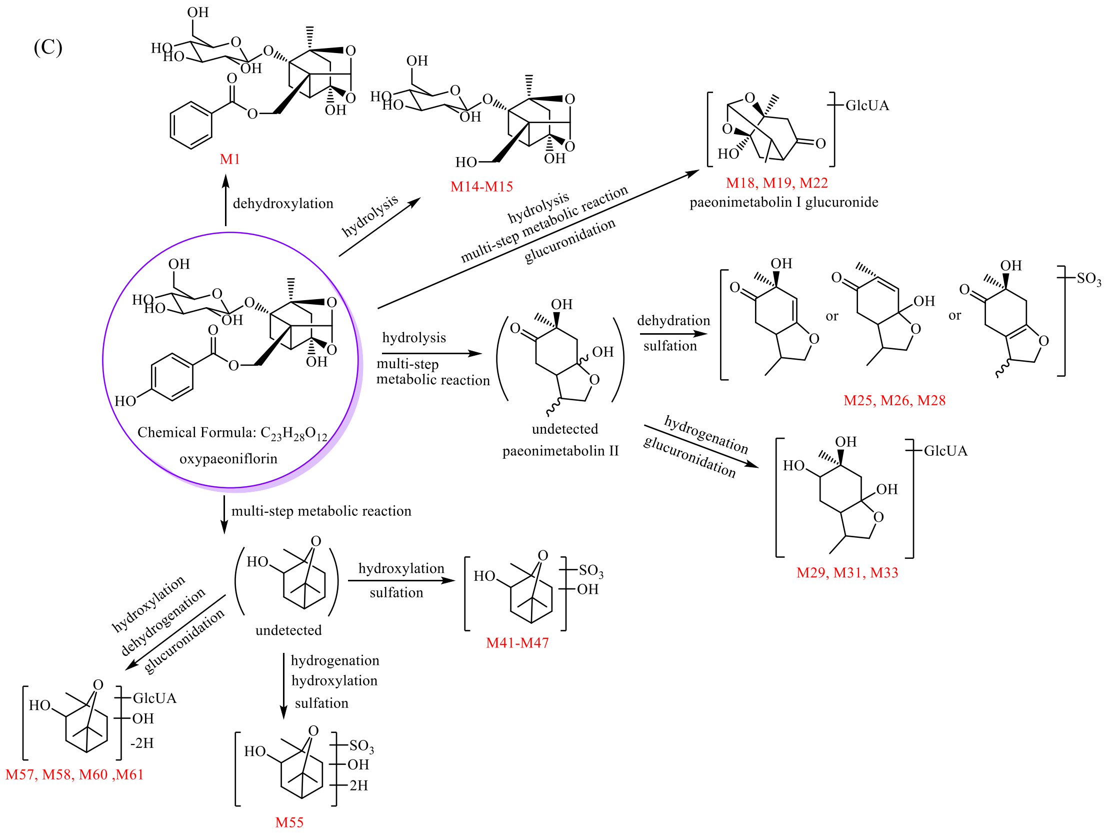

(C)
M1
dehydroxylation
hydrolysis
M14-M15
hydrolysis
multi-step metabolic reaction
glucuronidation
GlcUA
M18, M19, M22
paeonimetabolin I glucuronide
Chemical Formula: $C_{23}H_{28}O_{12}$
oxypaeoniflorin
hydrolysis
multi-step
metabolic reaction
undetected
paeonimetabolin II
dehydration
sulfation
or
or
$SO_3$
M25, M26, M28
hydrogenation
glucuronidation
GlcUA
M29, M31, M33
multi-step metabolic reaction
undetected
hydroxylation
sulfation
$SO_3$
OH
M41-M47
hydroxylation
dehydrogenation
glucuronidation
GlcUA
OH
-2H
M57, M58, M60 ,M61
hydrogenation
hydroxylation
sulfation
$SO_3$
OH
2H
M55

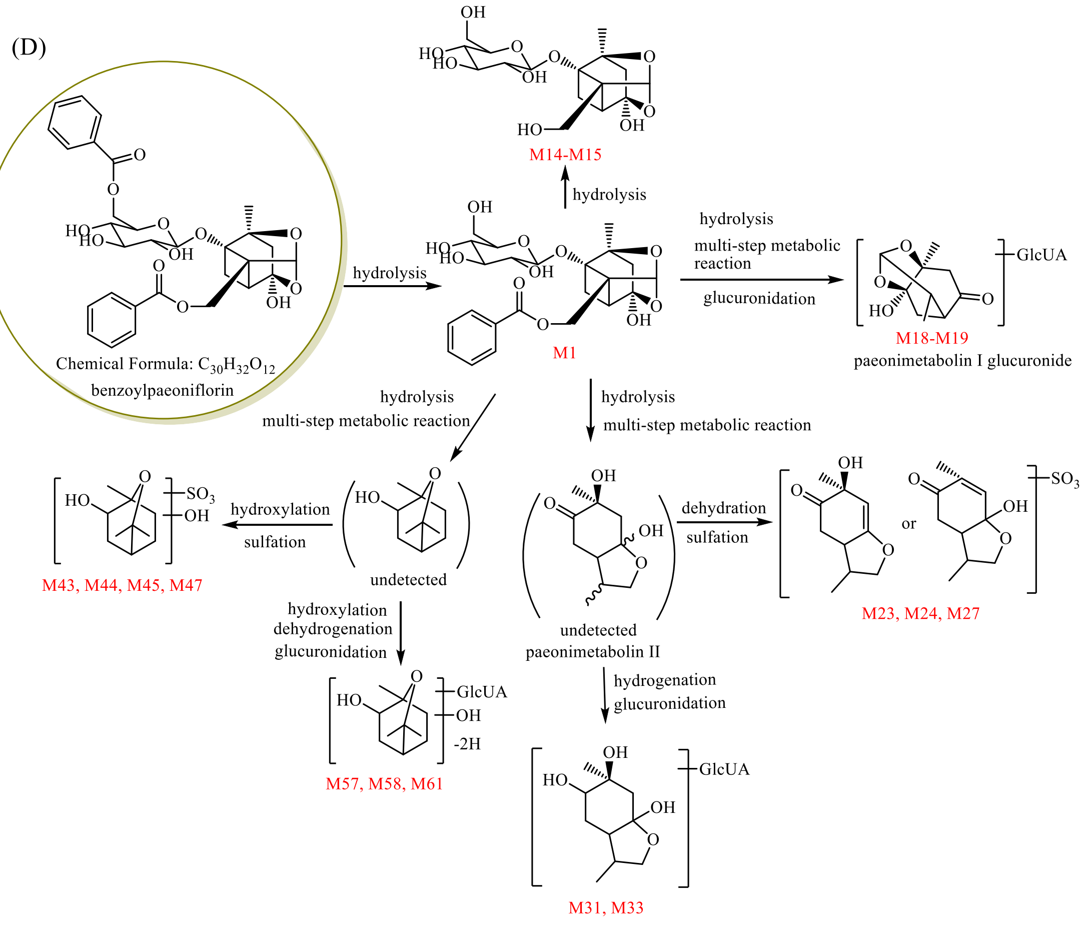
(D)
Chemical Formula: $C_{30}H_{32}O_{12}$
benzoylpaeoniflorin
hydrolysis
M1
hydrolysis
M14-M15
hydrolysis
multi-step metabolic reaction
glucuronidation
GlcUA
M18-M19
paeonimetabolin I glucuronide
hydrolysis
multi-step metabolic reaction
hydrolysis
multi-step metabolic reaction
SO3
OH
hydroxylation
sulfation
M43, M44, M45, M47
undetected
hydroxylation
dehydrogenation
glucuronidation
GlcUA
OH
-2H
M57, M58, M61
undetected
paeonimetabolin II
dehydration
sulfation
or
SO3
M23, M24, M27
hydrogenation
glucuronidation
GlcUA
M31, M33

(E)

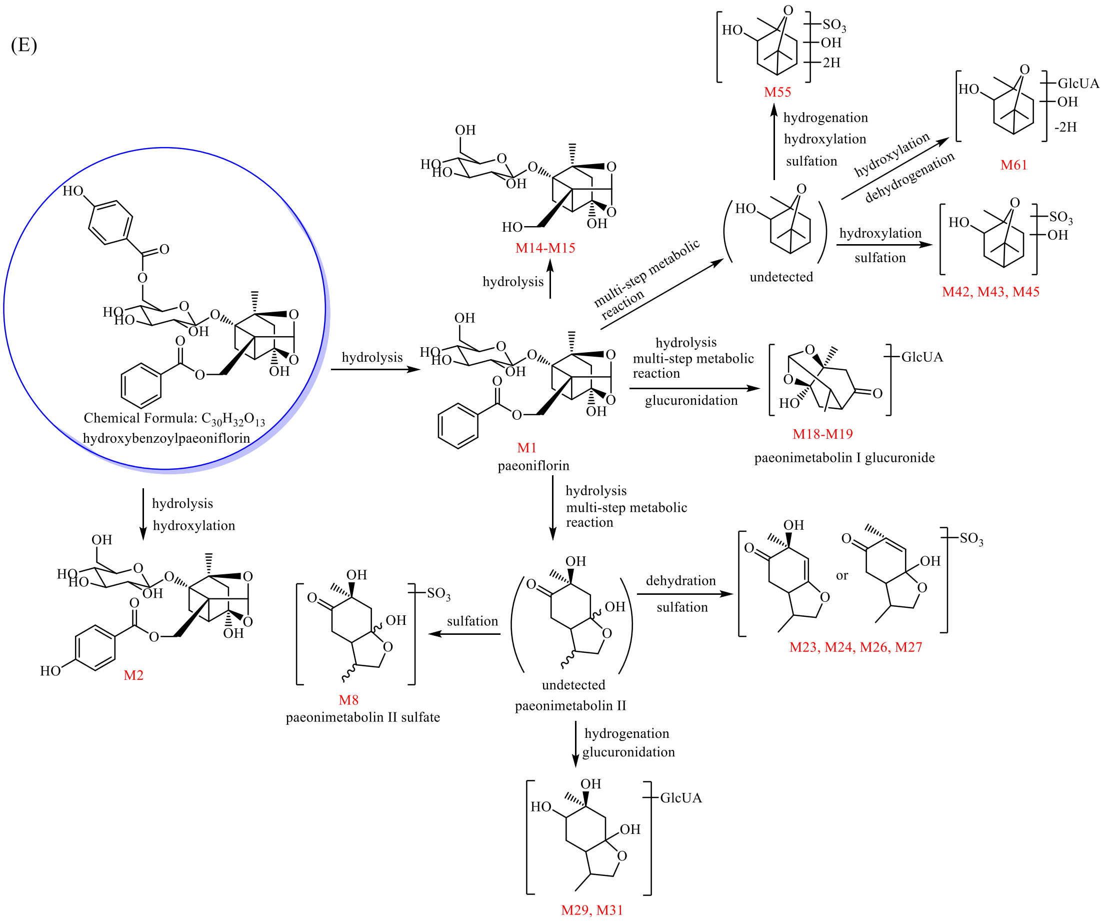

SO3
OH
2H
M55
hydrogenation
hydroxylation
sulfation
GlcUA
OH
-2H
M61
hydroxylation
dehydrogenation
M14-M15
hydrolysis
multi-step metabolic reaction
undetected
hydroxylation
sulfation
M42, M43, M45
Chemical Formula: C30H32O13
hydroxybenzoylpaeoniflorin
hydrolysis
hydrolysis
multi-step metabolic reaction
glucuronidation
M18-M19
paeonimetabolin I glucuronide
M1
paeoniflorin
hydrolysis
hydroxylation
hydrolysis
multi-step metabolic reaction
M2
sulfation
dehydration
sulfation
or
M23, M24, M26, M27
M8
paeonimetabolin II sulfate
undetected
paeonimetabolin II
hydrogenation
glucuronidation
M29, M31

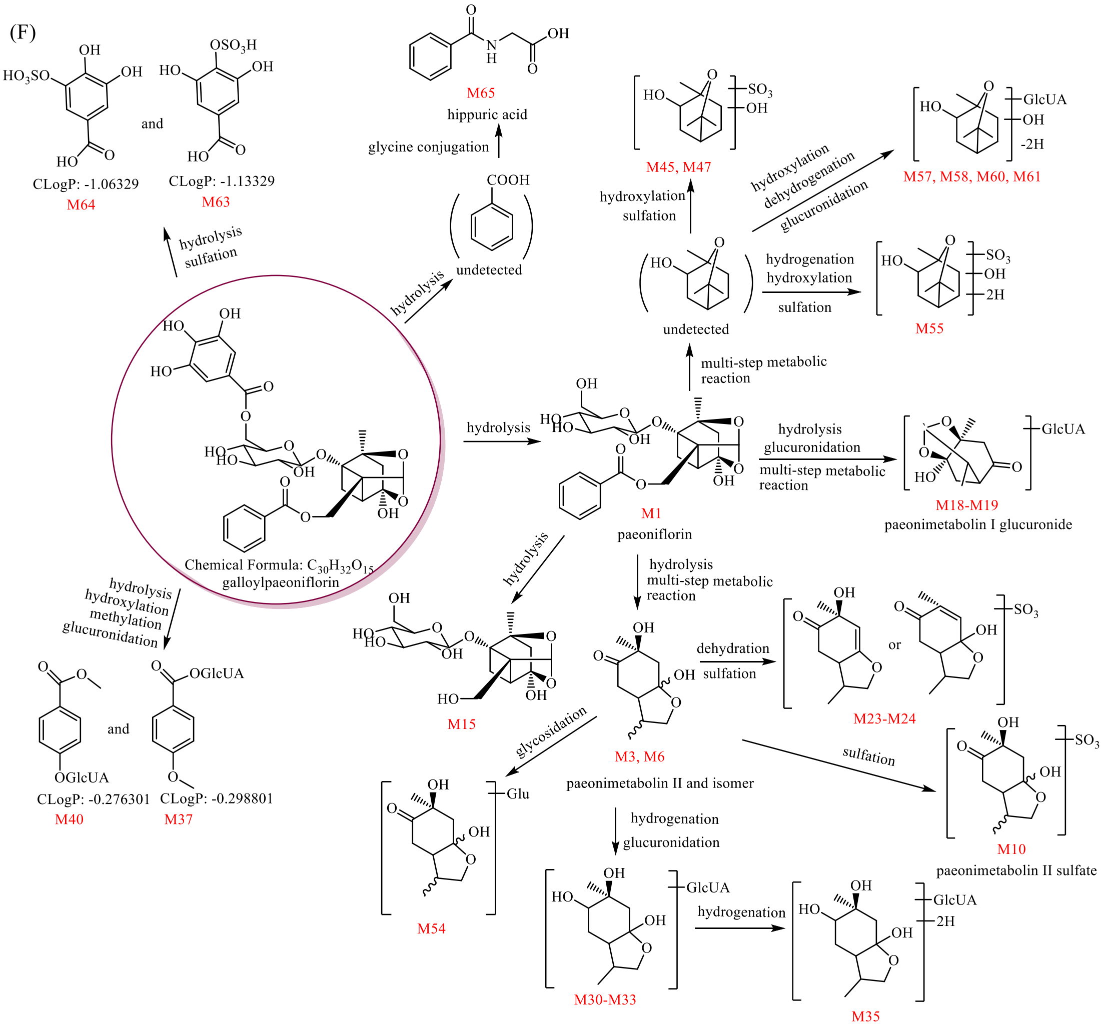
(F)
CLogP: -1.06329
M64
CLogP: -1.13329
M63
M65
hippuric acid
glycine conjugation
hydrolysis
sulfation
undetected
M45, M47
hydroxylation
sulfation
hydroxylation
dehydrogenation
glucuronidation
M57, M58, M60, M61
hydrogenation
hydroxylation
sulfation
M55
multi-step metabolic reaction
Chemical Formula: $C_{30}H_{32}O_{15}$
galloylpaeoniflorin
M1
paeoniflorin
hydrolysis
glucuronidation
multi-step metabolic reaction
M18-M19
paeonimetabolin I glucuronide
hydrolysis
hydroxylation
methylation
glucuronidation
CLogP: -0.276301
M40
CLogP: -0.298801
M37
M15
M3, M6
paeonimetabolin II and isomer
dehydration
sulfation
M23-M24
M10
paeonimetabolin II sulfate
glycosidation
M54
hydrogenation
glucuronidation
M30-M33
M35

(G)

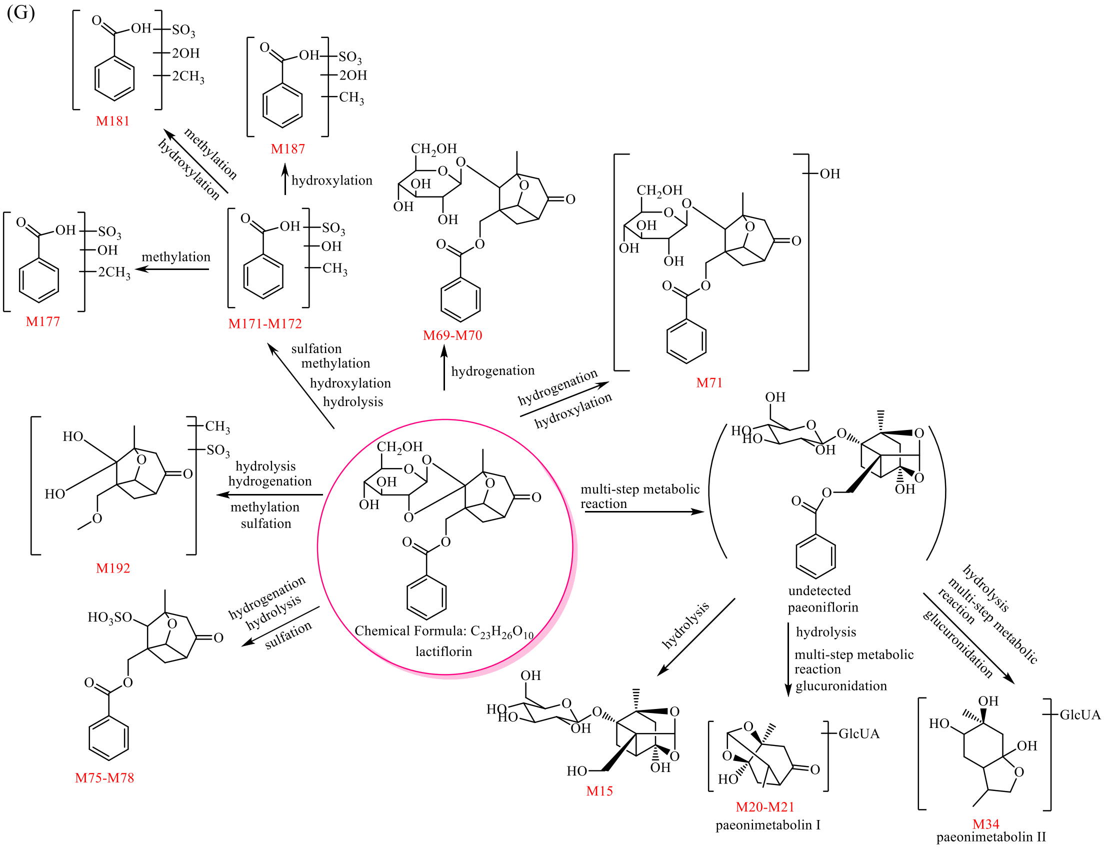


Figure S3. A, B, C, D, E, F, and G represent the proposed metabolic pathways of paeoniflorin, albiflorin, oxypaeoniflorin, benzoylpaeoniflorin, hydroxybenzoylpaeoniflorin, galloylpaeoniflorin, and lactiflorin in mice. The red numbers denote new metabolites.

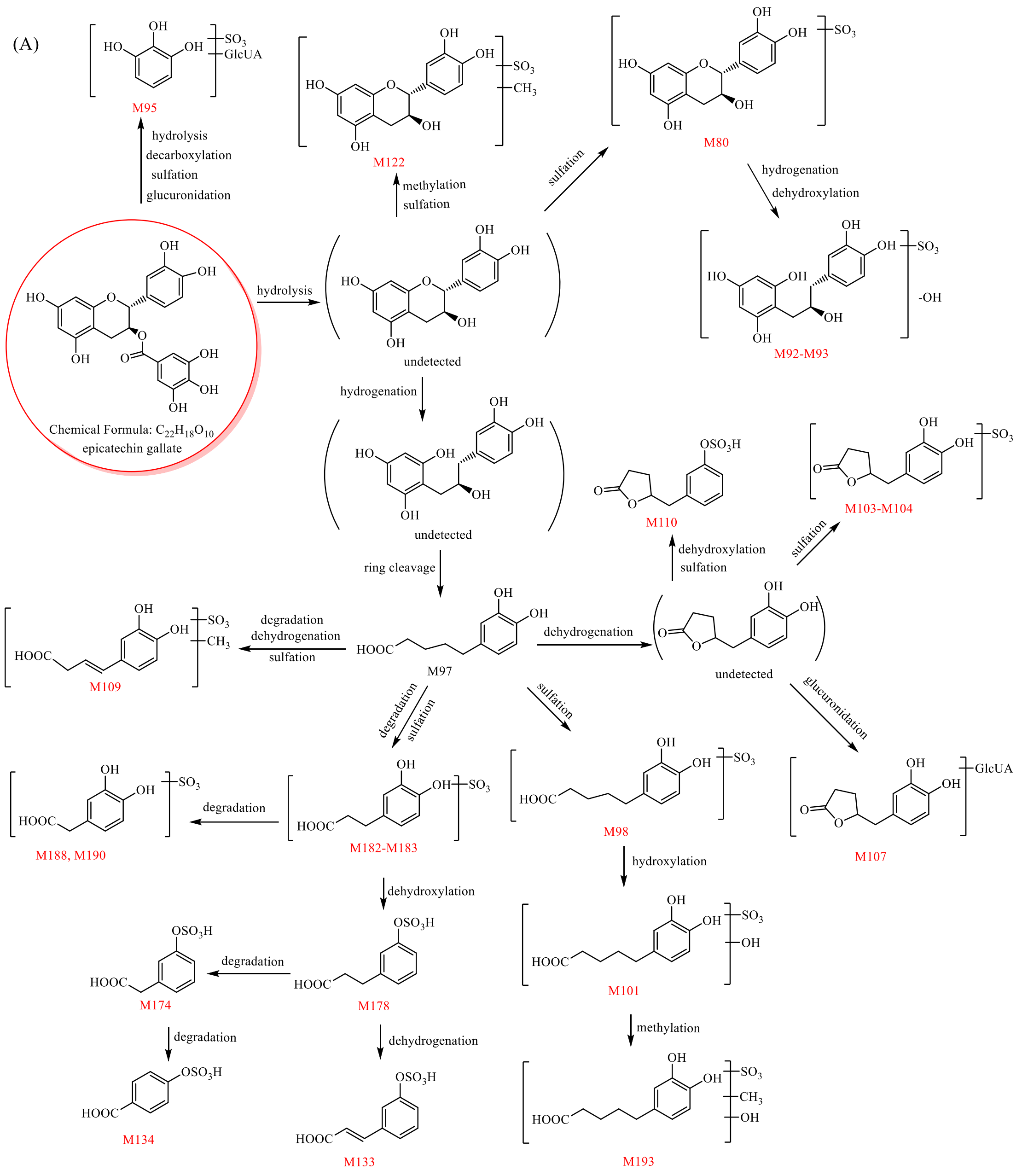
(A)
M95
hydrolysis
decarboxylation
sulfation
glucuronidation
M122
methylation
sulfation
sulfation
M80
hydrogenation
dehydroxylation
M92-M93
hydrolysis
undetected
Chemical Formula: C22H18O10
epicatechin gallate
hydrogenation
undetected
ring cleavage
M110
dehydroxylation
sulfation
sulfation
M103-M104
M109
degradation
dehydrogenation
sulfation
M97
dehydrogenation
undetected
degradation
sulfation
sulfation
glucuronidation
M188, M190
degradation
M182-M183
M98
M107
hydroxylation
dehydroxylation
M101
degradation
M174
M178
methylation
degradation
dehydrogenation
M134
M133
M193

(B)

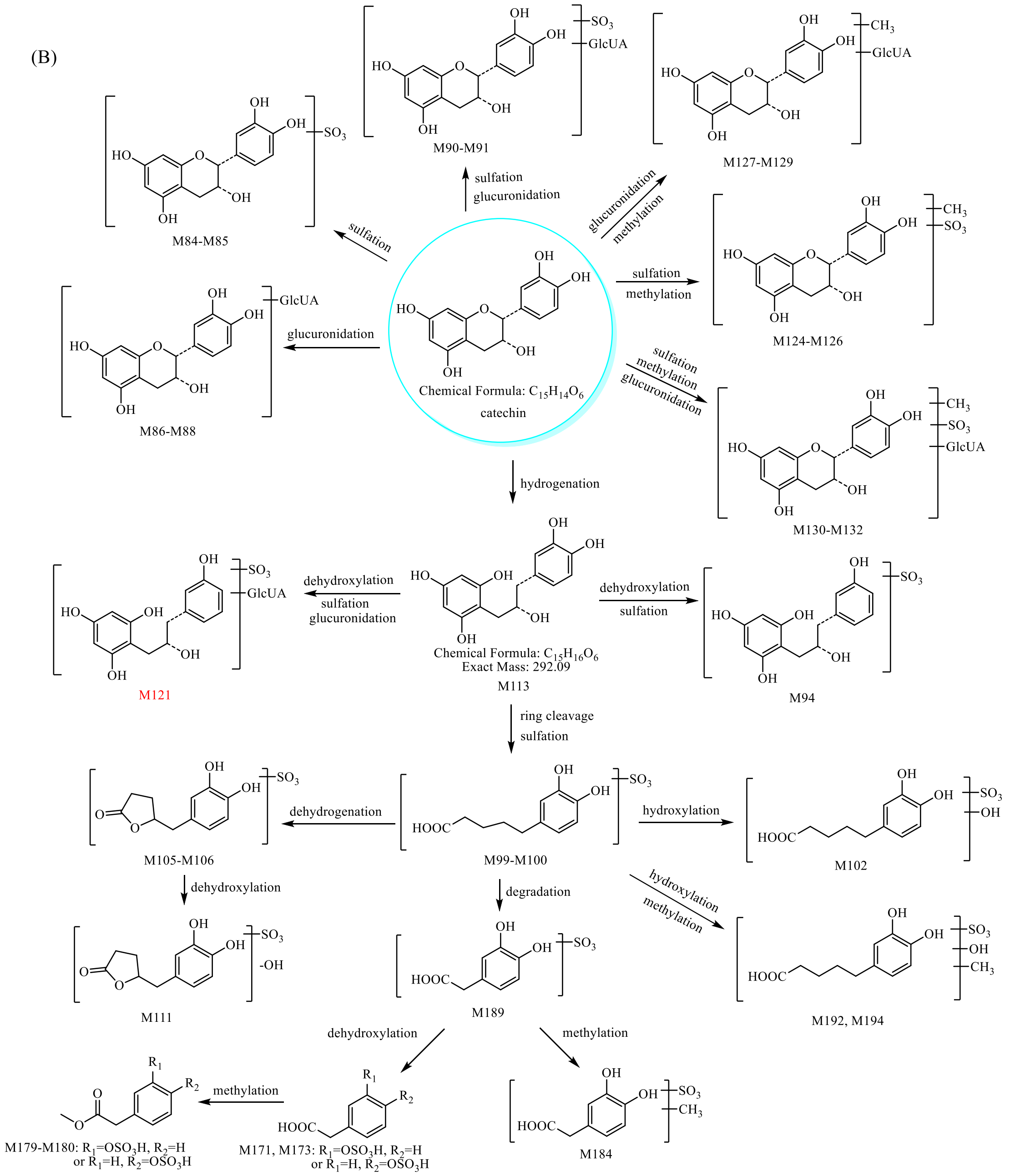


Figure S4. A and B represent the proposed metabolic pathways of epicatechin gallate and catechin in mice. The red numbers denote new metabolites.

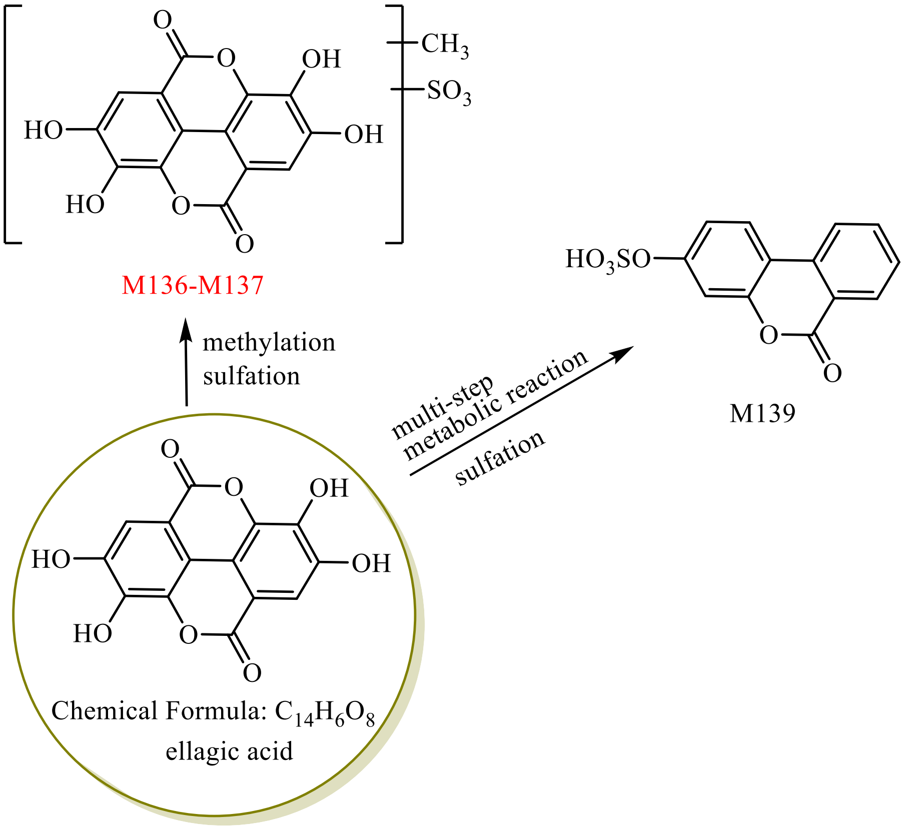


Figure S5. The proposed metabolic pathway of ellagic acid in mice. The red numbers denote new metabolites.

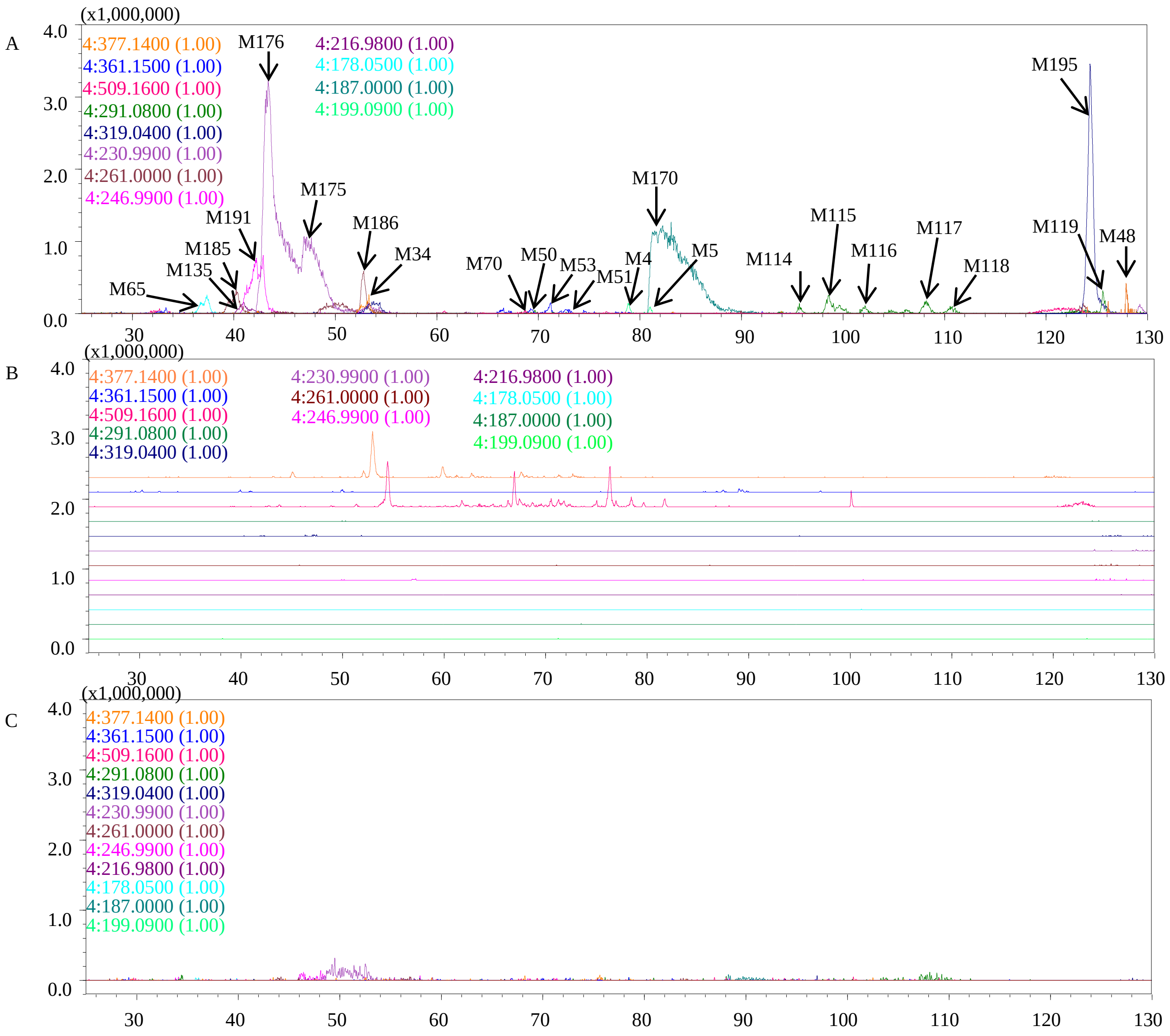
A
(x1,000,000)
4:377.1400 (1.00)
4:361.1500 (1.00)
4:509.1600 (1.00)
4:291.0800 (1.00)
4:319.0400 (1.00)
4:230.9900 (1.00)
4:261.0000 (1.00)
4:246.9900 (1.00)
4:216.9800 (1.00)
4:178.0500 (1.00)
4:187.0000 (1.00)
4:199.0900 (1.00)
M176
M175
M191
M185
M135
M65
M186
M34
M70
M50
M53
M51
M4
M170
M5
M114
M115
M116
M117
M118
M119
M195
M48
0.0
1.0
2.0
3.0
4.0
30
40
50
60
70
80
90
100
110
120
130
B
(x1,000,000)
4:377.1400 (1.00)
4:361.1500 (1.00)
4:509.1600 (1.00)
4:291.0800 (1.00)
4:319.0400 (1.00)
4:230.9900 (1.00)
4:261.0000 (1.00)
4:246.9900 (1.00)
4:216.9800 (1.00)
4:178.0500 (1.00)
4:187.0000 (1.00)
4:199.0900 (1.00)
0.0
1.0
2.0
3.0
4.0
30
40
50
60
70
80
90
100
110
120
130
C
(x1,000,000)
4:377.1400 (1.00)
4:361.1500 (1.00)
4:509.1600 (1.00)
4:291.0800 (1.00)
4:319.0400 (1.00)
4:230.9900 (1.00)
4:261.0000 (1.00)
4:246.9900 (1.00)
4:216.9800 (1.00)
4:178.0500 (1.00)
4:187.0000 (1.00)
4:199.0900 (1.00)
0.0
1.0
2.0
3.0
4.0
30
40
50
60
70
80
90
100
110
120
130

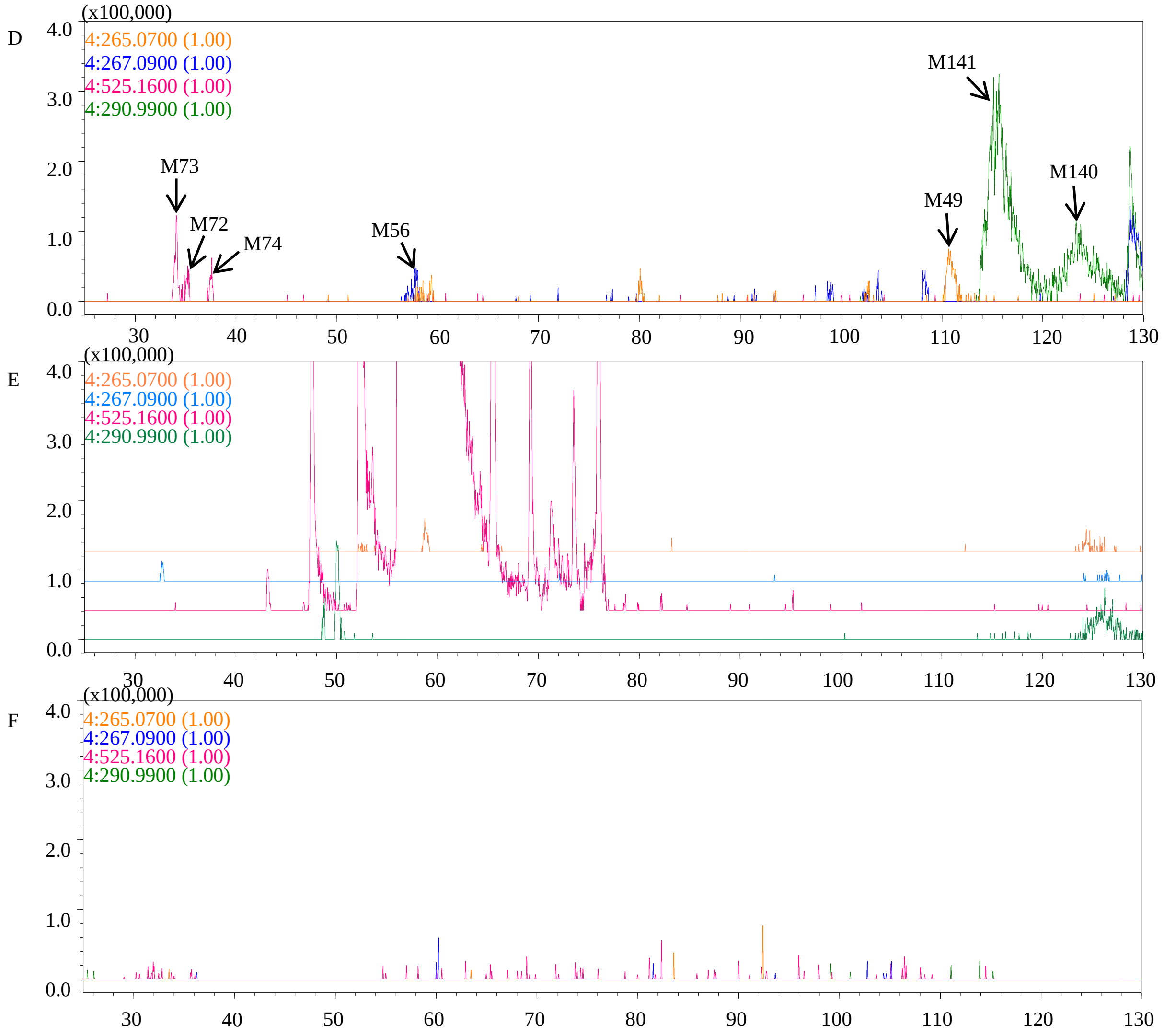

D
(x100,000)
4:265.0700 (1.00)
4:267.0900 (1.00)
4:525.1600 (1.00)
4:290.9900 (1.00)
M73
M72
M74
M56
M49
M141
M140
E
(x100,000)
4:265.0700 (1.00)
4:267.0900 (1.00)
4:525.1600 (1.00)
4:290.9900 (1.00)
F
(x100,000)
4:265.0700 (1.00)
4:267.0900 (1.00)
4:525.1600 (1.00)
4:290.9900 (1.00)

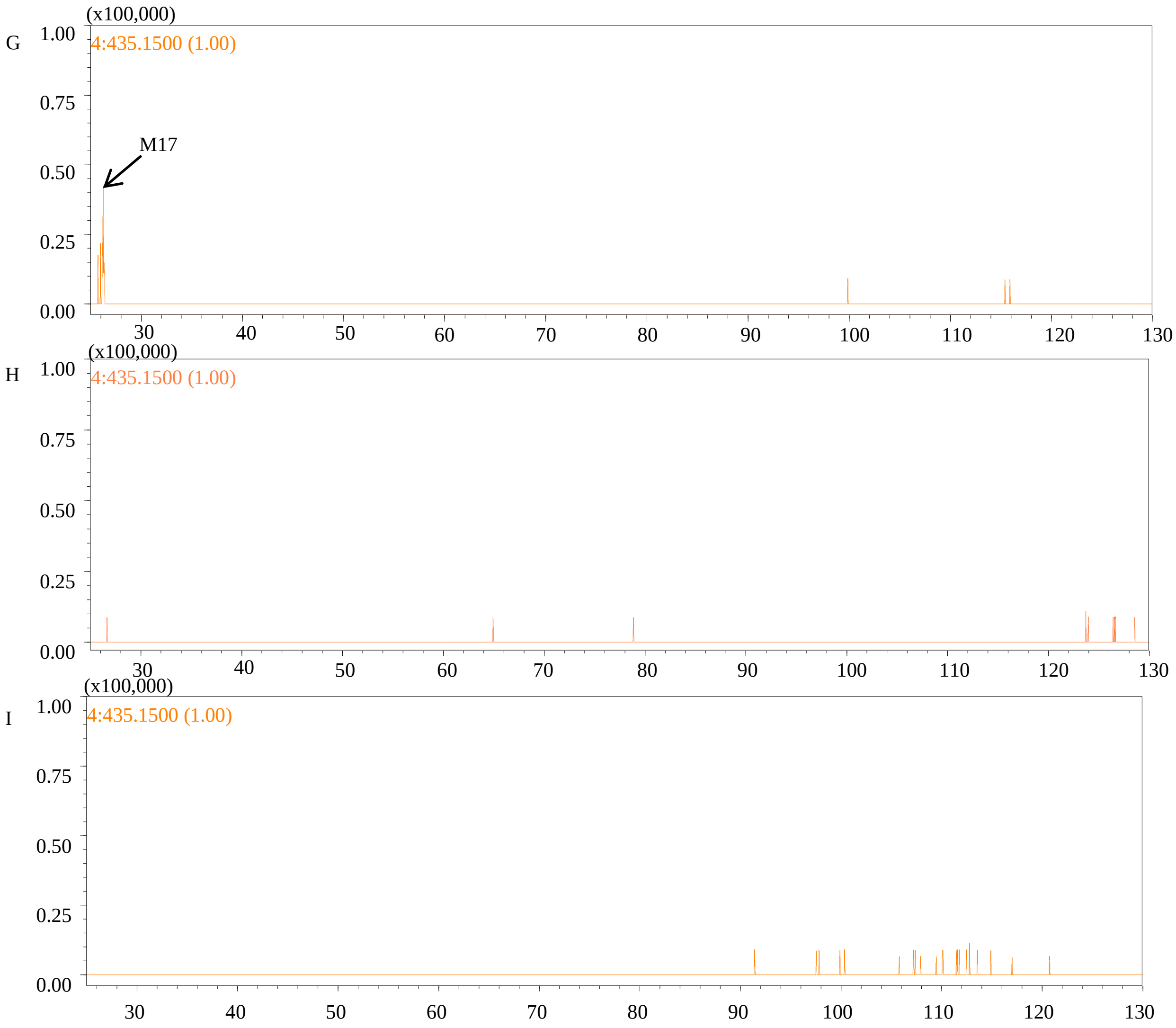


Figure S6. Extracted ion chromatograms (EICs) of 31 metabolites of Paeonia Radix Rubra from different samples. A, B, C represent the EICs of 23 metabolites of PRR group urine, PRR decoction dried powder, blank group urine, respectively; D, E, F represent the EICs of seven metabolites of PRR group feces, PRR decoction dried powder, blank group feces, respectively; G, H, I represent the EIC of one metabolite of PRR group plasma, PRR decoction dried powder, blank group plasma, respectively.
