# Supplementary material for: Metabolism of Paeoniae Radix Rubra and its 14 constituents in mice
Source: Front Pharmacol. 2022 Oct 4;13:995641. doi: 10.3389/fphar.2022.995641 (PMC9577399; doi:10.3389/fphar.2022.995641)
Supplement: Supplementary file 4 [file Table3.DOCX]

**Supplementary material**

**Table S3.** The formulae and fragment ions of 14 reference compounds of Paeonia Radix Rubra.

| Compound | Formula | Fragment ions (Negative Ion) |
| --- | --- | --- |
| paeoniflorin | C_23_H_28_O_11_ | 449.1476, 431.1346, 421.1468, 359.1336, 345.1202, 327.1092, 283.0802, 165.0598 |
| albiflorin | C_23_H_28_O_11_ | 449.1478, 357.1133, 327.1018, 283.1030, 195.0602, 181.0512 |
| oxypaeoniflorin | C_23_H_28_O_12_ | 477.1412, 465.1351, 447.1304, 375.1306, 345.1196, 327.1014, 299.0745, 281.0528, 179.0578, 165.0604 |
| benzoylpaeoniflorin | C_30_H_32_O_12_ | 553.1678, 535.1574, 461.1486, 431.1302, 413.1281, 387.1380, 309.1012, 265.1036 |
| hydroxybenzoylpaeoniflorin | C_30_H_32_O_13_ | 5581.1677, 569.1638, 551.1481, 477.1356, 459.1261, 441.1208, 429.1155, 385.1198, 281.0600, 263.0572 |
| benzoyloxypaeoniflorin | C_30_H_32_O_13_ | 581.1678, 569.1624, 551.1588, 479. 1568, 477.1348, 447.1246, 431.1302, 429.1218, 403.1016, 281.0688, 263.0636 |
| galloylpaeoniflorin | C_30_H_32_O_15_ | 613.1485, 509.1352, 491.1162, 463.1217, 435.1308, 417.1164, 331.0655, 313.0522, 271.0418, 253.0306, 235.0247, 211.0168 |
| lactiflorin | C_23_H_26_O_10_ | 371.1035, 339.1066, 283.0729, 281.0736, 237.0782, 231.0640, 177.0539 |
| epicatechin gallate | C_22_H_18_O_10_ | 331.0472, 303.0436, 289.0686, 285.0336, 259.0513, 247.0538, 245.0802, 243.0298, 241.0466, 229.0468 |
| catechin gallate | C_22_H_18_O_10_ | 331.0472, 289.0686, 285.0336, 247.0538, 245.0802, 241.0466, 229.0468, 205.0449, 187.0388, 163.0772 |
| catechin | C_15_H_14_O_6_ | 271.0573, 247.0562, 245.0765, 205.0483, 203.0633, 175.0702, 137.0206, 125.0186 |
| ellagic acid | C_14_H_6_O_8_ | 284.0013, 257.9804, 245.0113, 229.9937, 217.0121, 201.0232, 173.0198, 157.0285, 145.0313, 129.0331 |
| 3,3'-di-*O*-methyl ellagic acid | C_16_H_10_O_8_ | 314.0032, 298.9806, 270.9869, 242.9931 |
| methyl gallate | C_8_H_8_O_5_ | 168.0109, 124.0232 |
